# Understanding the impact of developmental coordination disorder on Belgian children and families: A national survey study

**DOI:** 10.1371/journal.pone.0320311

**Published:** 2025-04-22

**Authors:** Amy De Roubaix, Griet Warlop, Dorine Van Dyck, Delphine Van Crombrugge, Silke Van den Abbeele, Melissa Licari, Hilde Van Waelvelde, Lynn Bar-On

**Affiliations:** 1 Faculty of Medicine and Health Sciences. Department of Rehabilitation Sciences, Ghent University, Ghent, Belgium; 2 Faculty of Medicine and Health Sciences. Department of Movement and Sports Sciences, Ghent University, Ghent, Belgium; 3 Department of Neuropsychology and Speech Therapy, Queen Fabiola Children’s University Hospital (HUDERF) - Hôpital Universitaire de Bruxelles (H.U.B), Université libre de Bruxelles (ULB), Brussels, Belgium; 4 Department of Pediatric Neurology, Queen Fabiola Children’s University Hospital (HUDERF) - Hôpital Universitaire de Bruxelles (H.U.B), Université libre de Bruxelles (ULB), Brussels, Belgium; 5 Telethon Kids Institute, The University of Western Australia, Perth, Western Australia, Australia; Brunel University London, UNITED KINGDOM OF GREAT BRITAIN AND NORTHERN IRELAND

## Abstract

**Background:**

Developmental Coordination Disorder (DCD) is an under-recognized and often trivialized neurodevelopmental condition impacting five to six percent of children. This study aimed to map the impact of DCD on children and their families in Belgium.

**Methods:**

The Australian ‘Impact for DCD’ questionnaire was translated and adapted to suit the Belgian context. Parents of 4-to-18-year-old children living in Belgium with movement difficulties consistent with DCD were invited to complete the online survey covering diagnosis, activities, school, therapy, and social and emotional impact.

**Results:**

A total of 491 children were included in the analyses. First concerns emerged primarily at home (61.4%) at age 3.3 ± 2.25y, with help sought at age 4.7 ± 2.57y. Formal diagnosis occurred at age 6.9 ± 2.36y, with DCD (76.5%) and/or dyspraxia (38.4%) the most frequently received terms. DCD was generally unknown, especially within school settings. Nearly one in four children (23.2%) repeated a year of school. Reduced sleep quality (50.6%), fatigue after school (76.0%), difficulties with toilet training (47.9%) and speech articulation (52.3%), as well as elevated emotional problems (52.7%) and peer-related issues (46.4%), were prevalent. Most children received therapy (89.2%) but 59.1% of parents did not feel adequately supported to assist their child. Moreover, 37.5% of parents regularly took time off work to facilitate their child’s therapy attendance, while 49.1% had reduced their working hours or were contemplating doing so (16.7%). Parents expressed concerns about their child's future and well-being, with a prominent plea for guidance on supporting their child and increased awareness. Parents reported important strengths in their children, including empathy, creativity, cognitive abilities, perseverance, and good social and/or language skills.

**Conclusions:**

These results highlight the significant impact of DCD from parents’ perspectives. The next step is to explore ways to empower and support parents, raise awareness, and collaborate with policymakers to address these challenges.

## Introduction

Developmental Coordination Disorder (DCD) is a neurodevelopmental condition (NDC) impacting 5–6% of school-aged children [1]. Individuals with DCD experience significantly more difficulties with the acquisition and execution of coordinated motor skills relative to their peers and learning opportunities, which significantly impacts various aspects of their lives, such as school productivity, activities of daily living, and professional activities. The difficulties are present from an early age and cannot be explained by another medical condition (e.g., cerebral palsy, muscular dystrophy, intellectual disability). The challenges associated with DCD often extend beyond motor skills, affecting various domains of functioning. The difficulties evoke feelings of failure, resulting in short-term frustrations, diminished self-esteem, and long-term risks of depression and anxiety [2]. Consequently, individuals often avoid physical activity and reduce their engagement, potentially leading to diminished social skills, fewer friendships, and increased feelings of isolation [3]. DCD impacts not only the individual but also the entire family, with families engaging in fewer social activities and feeling restricted in their daily lives [4]. Parents may experience reduced well-being, heightened worries, and increased demands on their time and energy to meet their child's needs, resulting in significant parenting stress [5]. Importantly, parents often face the need to adapt their careers, reduce working hours, or cease working altogether, leading to financial strains due to decreased income and limited access to financial support [4,6].

Heterogeneity in DCD is evident not only in the severity and categories of impacted domains but also in the co-occurrence with other conditions. Up to 70% of children with DCD are reported to experience one or more additional conditions, including Autism Spectrum Disorder (ASD), Attention-Deficit/Hyperactivity Disorder (ADHD), specific learning disorders (e.g., dyslexia, dyscalculia), and childhood apraxia of speech [6–8]. In addition to requiring diverse support systems, it is crucial to recognize that children with concurrent conditions often encounter even poorer outcomes, including lower quality of life [9] and emotional health issues [10]. While DCD is estimated to affect at least one child in every classroom, the condition is among the least known childhood conditions and is too often unrecognized [6]. In Germany and the UK, only 59% of clinicians across various specialties were familiar with DCD [11]. In Canada, 100% of paediatric occupational therapists demonstrated knowledge on DCD, contrasting with 41% of paediatricians and 22% of general practitioners [12]. The lack of clinician awareness adds to the under recognition of the condition, delays in diagnosis, and minimization of parental concerns, leaving parents feeling isolated and unsupported [13–15]. There is also a lack of awareness of DCD in the education sector, where only 23% of Canadian teachers [16] and 35% of Australian teachers [17] were found to be familiar with DCD. The lack of awareness of DCD at schools may cause misinterpretation and trivialisation of the perceived difficulties as at least some of these children were wrongly considered ‘lazy’ and ‘not trying hard enough’ [18,19]. In terms of general public awareness, a Canadian study reported that only 6% of parents had ever heard of DCD [16]. Parents may struggle to comprehend the cause of their child's difficulties and may not realize that there could be an underlying diagnosable condition. Consequently, they may experience frustration when their child faces challenges in completing basic tasks [20]. This frustration can exacerbate strained family dynamics and contribute to the child's emotional challenges.

The “Impact for DCD” movement was created in an effort to comprehensively understand the wide-ranging impact of DCD and advocate for systemic change. Initially launched in Australia, the team of Licari et al. (2021) developed an extensive survey aimed at mapping the impact of DCD on children and their families, and identify areas most important to families requiring change [6]. Numerous countries have since joined this effort to comprehensively assess the global impact. It is crucial to conduct studies in each country, as the impact may vary significantly depending on the healthcare and educational system, access to resources, social support networks, culture, and general awareness of the condition. Thus far, research findings discussing the impact of DCD have been published from Australia [6,21,22], Canada [23–25], and the United States of America [26]. This study aims to contribute to this movement by mapping the impact of DCD in Belgium, which has been largely unexplored except for one qualitative study on early DCD features [27]. Identifying challenges and areas for improvement within the Belgian context could help define areas of focus and prioritize support for families. Results of this study can inform policy decisions related to education, healthcare, and social support services.

## Methods

This study was approved by the Medical Ethics committee of the University Hospital Ghent (Belgium) (ONZ-2022–0203) and registered at ClinicalTrials.Gov (NCT05499143). After reading the informed consent form, all participants provided digital consent by affirmatively answering the consent question before participating.

### Survey and participants

Following the guidelines for the process of cross-cultural adaptation of self-report measures [28], the Australian Impact for DCD survey [6] was translated to Dutch and French and culturally adapted for Belgian context. This process involved four interpreters translating the original English survey into Dutch (n = 2) and French (n = 2). Subsequently, a synthesis of the Dutch and French translations was created during a meeting after which four different interpreters translated these syntheses back to English. A meeting was held to discuss any differences in interpretation that required adaptation to the Dutch and French version. Written reports were provided at each stage. Next, an extensive expert committee (including eight translators, one parent of a child with DCD, one representative of the Belgian non-profit parental support organisation for DCD “Dyspraxis”, four allied health professionals, and eight researchers in the field of DCD) reviewed the Belgian survey and discussed the survey items. Similar to the Australian survey, questions were included regarding diagnosis, therapy, activities and participation, education, social and emotional impact, and the impact on the family. Three subscales from the Strengths and Difficulties Questionnaire (SDQ) [29] were used: emotional challenges, peer challenges, and prosocial behaviour. In the absence of Belgian norms for children 4 to 10y, British SDQ norms were used, while Belgian norms were used for children aged eleven to eighteen. The original survey was complemented with several novel themes including toilet training, speech articulation, and strengths of the children. The survey included both multiple choice and open questions and took an average of 45 minutes to complete (S1 File).

The survey was launched August 17^th^ 2022 and remained available until December 17^th^ 2022. Data collection was supported by REDCap electronic data capture tools hosted at the University of Ghent [30]. Parents of 4-to-18-year-old children living in Belgium with movement difficulties consistent with DCD (i.e., that could not be explained by another medical condition) were invited to complete the online survey. The survey focused on parental experiences, thus all responses were provided by parents. The study was promoted through social media platforms, official University communication, and the non-profit parental support organisation Dyspraxis. Invitations to participate were sent via letters and emails to a wide range of Belgian paediatric healthcare providers (e.g., physiotherapists, occupational therapists, paediatricians, rehabilitation centres, speech-language therapists, psychomotor therapists) and special education schools, ensuring nationwide coverage. Participants were excluded if they did not live in Belgium, did not meet the age criterion (4–18 years), lacked movement difficulties consistent with DCD, had another medical condition explaining the movement difficulties, or did not fully complete the questionnaire.

### Analysis

Both quantitative and qualitative analyses were performed. Exploring the open-ended responses provided qualitative insights that complemented the survey data, offering a richer understanding of the topic. For each item, the total number of participants who selected a particular answer was reported, along with the percentage relative to the full sample or the relevant subsamples when follow-up questions were involved. Quantitative analysis conducted in JASP [31] included a comparison between responses from children with and without co-occurring conditions, which were binary-coded as either present or absent. Due to the non-normal distribution of the numerical data, Mann-Whitney U tests were employed, with effect sizes reported as Rank-Biserial r. Significance level was set at p < 0.05. For categorical data, chi-square tests were conducted. Qualitative inductive thematic analysis was conducted to discern the underlying concepts from open-ended questions. Two researchers independently coded all responses using NVivo 14 software [32]. The codes were then organized into themes through an iterative process. Any discrepancies were resolved through discussion. To enhance the trustworthiness of the data collection and analysis, the inclusion and exclusion criteria were thoroughly addressed in the initial part of the questionnaire. Investigator triangulation was implemented by double-coding all open-ended questions and engaging in discussions within the multidisciplinary research team to validate the results. Furthermore, heightened reflexivity was fostered to ensure thorough and open-minded investigation.

## Results

### Participants

Parents of 1256 children initially consented to participate. A total of 477 families of 491 children were withheld for analysis after excluding participants whose child did not live in Belgium (n = 97), was not aged 4–18 years (n = 19), did not experience movement difficulties consistent with DCD (n = 22), had another medical condition that could explain the movement difficulties (n = 329), or because the survey was not completed (n = 298). Sample characteristics are described in Table 1. The study sample (n = 491) with mean age 10.4 ± 3.37y predominantly consists of term-born, male children, aged 7–12 years, the majority being eldest children, from families with medium to high incomes, primarily residing in Flanders. At least one additional diagnosis was present in 59.5% of children with ADHD being the most prevalent co-occurring condition (31.0%), followed by ASD (22.8%) and specific learning disorders (21.8%).

**Table 1:** Sample characteristics (n = 491).

	N	%
**Sex**		
Male	373	76.0
Female	118	24.0
**Gestational age**		
<28 weeks	3	0.6
28 - 32 weeks	11	2.2
32 - 37 weeks	63	12.8
>37 weeks	412	83.9
Missing	2	0.4
**Age**		
4–6 years	64	13.0
7–9 years	145	29.5
10–12 years	156	31.8
13–15 years	80	16.3
16–18 years	46	9.4
**Position in family**		
Eldest	199	40.5
Middle	55	11.2
Youngest	169	34.4
Only child	68	13.8
**Monthly net family income**		
Low (<1962 euros)	36	7.3
Medium (1962–3924 euros)	190	38.7
High (>3924 euros)	205	41.8
I prefer not to share	60	12.2
**Region of residence**		
Flanders	377	76.8
Wallonia	99	20.2
Brussels Capital Area	15	3.1
**Co-occurring conditions**		
ADHD	152	31.0
Autism spectrum disorder	112	22.8
Specific learning disorder	107	21.8
Speech-language disorder	65	13.2
Dysgraphia	38	7.7
Anxiety or depression	24	4.9
Epilepsy	10	2.0
Hearing problems	4	0.8
Tic disorder	3	0.6
Cerebral visual impairment	3	0.6
Other	19	3.9

Abbreviations: ADHD, Attention Deficit Hyperactivity Disorder; n, number.

### Diagnosis

#### Diagnostic trajectory.

**Fig 1:**
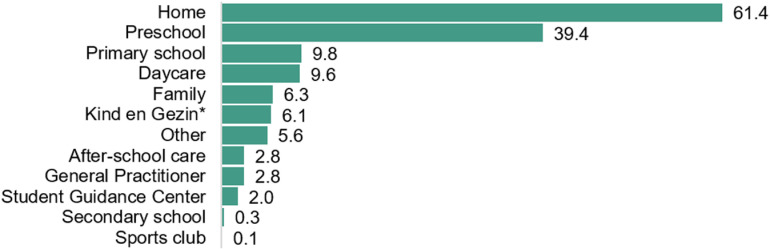
Percentage of environmental settings that first raised concerns (n = **491): first concerns emerged primarily at home.** Respondents could select multiple answers. *Kind en Gezin translates to ‘Child and Family’; this is the Flemish government agency in Belgium responsible for childcare and family welfare services).

**Fig 2:**
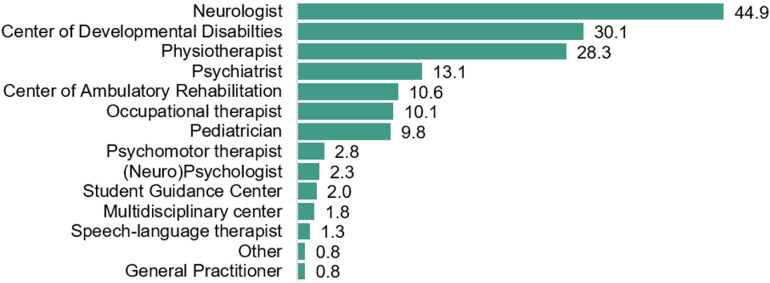
Professionals (%) who formally diagnosed the child with movement difficulties (n = **396): Various professionals and institutions provide the diagnosis of Developmental Coordination Disorder.** Respondents could select multiple answers.

**Fig 3:**
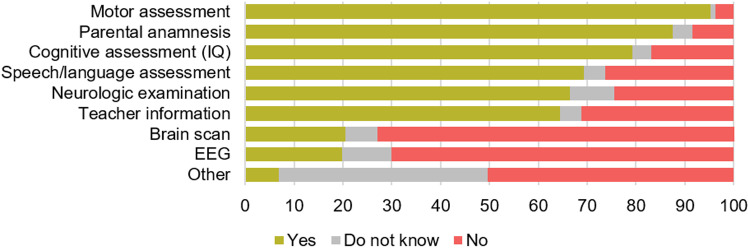
Assessments conducted within the diagnostic process of movement difficulties (%) (n = **396): Motor assessments and parental anamnesis were common, while teacher consultations were less frequent.** Respondents could select multiple answers. Abbreviations: IQ, intelligence quotient; EEG, electro-encephalogram.

Concerns about the child's movements were first raised by parents when the children were on average 3.3 ± 2.25y. These concerns were identified primarily at home (n = 243, 61.4%), followed by reports from preschool (2.5 to 5y) (n = 156, 39.4%), primary schools (6 to 12y) (n = 39, 9.8%), or daycare facilities (<2.5y) (n = 38, 9.6%) (Fig 1). Parents sought help when their children were at the mean age of 4.7 ± 2.57y. A total of 80.7% (n = 396) of the children had received a formal diagnosis at the mean age of 6.9 ± 2.36y. There was no significant difference between children with or without co-occurring conditions in age of first concern (U = 28254.0, p = 0.74, *r* = −0.017; S2 File) or diagnosis (U = 16641.0, *p* = 0.428, *r* = −0.048; S2 File). The most common diagnostic terms were DCD (n = 294, 76.5%) and/or dyspraxia (n = 152, 38.4%) (Table 2), which were mostly diagnosed by neurologists (n = 178, 44.9%), Centres of Developmental Disabilities (n = 120, 30.3%), physiotherapists (n = 112, 28.3%), or psychiatrists (n = 52, 13.1%) (Fig 2). If a physiotherapist diagnosed the movement difficulties, it typically involved collaboration with a medical doctor (98 out of 112 cases, 77.5%). During the diagnostic process, motor assessments (95.2%) and parental anamnesis (87.6%) were frequently conducted alongside numerous other assessments (Fig 3). According to parents, teachers were consulted in 64.4% of the cases. In 95 children (19.3%), no formal diagnosis of movement difficulties was made to date, but rather their movement difficulties were commonly described as, e.g., ‘a risk/ characteristics of DCD’, ‘motor coordination problems’ or ‘non-fluent motor skills’ (Table 2).

**Table 2:** Diagnoses and descriptions of movement difficulties.

	N	%
**Formal diagnosis of movement difficulties (n** = **396)**		
Developmental Coordination Disorder (DCD)*	294	74.2
Dyspraxia	152	38.4
Hypotonia	32	8.1
Hypermobility	29	7.3
Trouble d’Acquisition de la Coordination (TAC)	8	2.0
Sensory Integration Disorder	1	0.3
Minimal Brain Damage	1	0.3
Other	1	0.3
**Description of movement difficulties in the absence** **of a formal diagnosis (n** = **95)**		
Risk/ characteristics of DCD	57	60.0
Motor coordination problems	46	48.4
Non-fluent motor skills	46	48.4
Clumsiness	45	47.4
Delayed motor development	30	31.6
Motor planning difficulties	25	26.3
Delayed motor milestones	16	16.8
Not described by professional	16	16.8
Reflex Integration Disorder	9	9.5
Other	1	1.1

Respondents could select multiple answers.

*The DCD category encompassed the English term (Developmental Coordination Disorder), the literal translation in Dutch (coördinatie ontwikkelingstoornis), and the literal translation in French (trouble développemental de la coordination; TDC).

Abbreviations: DCD, Developmental Coordination Disorder; n, number.

#### Awareness and impact of a diagnosis of DCD.

**Fig 4:**
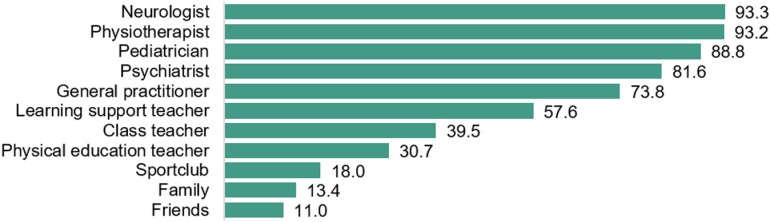
Percentage of specialists, school settings, and friends/family contacted by the family who knew the diagnosis of Developmental Coordination Disorder: most healthcare professionals knew DCD, but knowledge was limited in educational and leisure settings, as well as among family and friends.

Most parents (n = 232, 58.6%) had not heard of DCD before their child's diagnosis but agreed that receiving a diagnosis was beneficial both for themselves (n = 362, 91.4%) and for their child (n = 339, 85.6%). When asked to elaborate on the value of diagnosis, parents commented that for their children, a diagnosis translated to better self-understanding and increased understanding from both parents and teachers, more support at school, and access to therapy (n = 339). However, in cases where the diagnosis was deemed unhelpful, parents perceived a larger gap between their child and others and a negative impact on the child's self-confidence (n = 57). For parents (n = 362), confirmation of the diagnosis entailed acknowledgment, validation, and clarification of their concerns, fostering acceptance, and providing reassurance that they are not imagining or exaggerating the perceived difficulties, thereby alleviating a period of uncertainty, stress, doubt, and frustration. Confirmation of a diagnosis empowered parents to articulate their child's challenges more effectively, leading to improved understanding and fewer negative remarks from others. Additionally, they could offer better support, increased understanding, and patience. However, instances where parents felt unsupported by the diagnosis primarily revolved around a lack of post-diagnosis assistance and ongoing uncertainty as they had to “figure everything out by themselves” (n = 34). While most medical doctors and physiotherapists attended by the family demonstrated familiarity with the term DCD, approximately one in four general practitioners did not know DCD (Fig 4). Furthermore, less than half of class teachers and one third of physical education (PE) teachers had prior knowledge of DCD. Finally, 11–13% of friends and family had heard of the condition.

### Functional impact

#### Leisure and sporting activities.

Parents reported that most children (n = 372, 75.8%) engaged in organized leisure activities. Less than half of the children (n = 222, 45.2%) were reported to enjoy participation in organized sports activities while 35.2% (n = 173) enjoyed them sometimes and 19.6% (n = 96) not at all. These proportions did not differ depending on the presence of co-occurring conditions (χ² = 0.3, p = 0.596 and χ² = 3.0, p = 0.224; S2 File). Among the children, 82.5% (n = 405) did not engage in 60 minutes of daily moderate-to-vigorous physical activity, with no difference between children with or without co-occurring conditions (χ² = 2.6, *p* = 0.454; S2 File). One third of parents (n = 170, 34.6%) voiced concerns regarding the potential adverse effects of reduced physical activity on their child's health and 55.6% (n = 273) reported a lack of available leisure activities tailored to their child's needs.

#### Activities of daily life.

**Fig 5:**
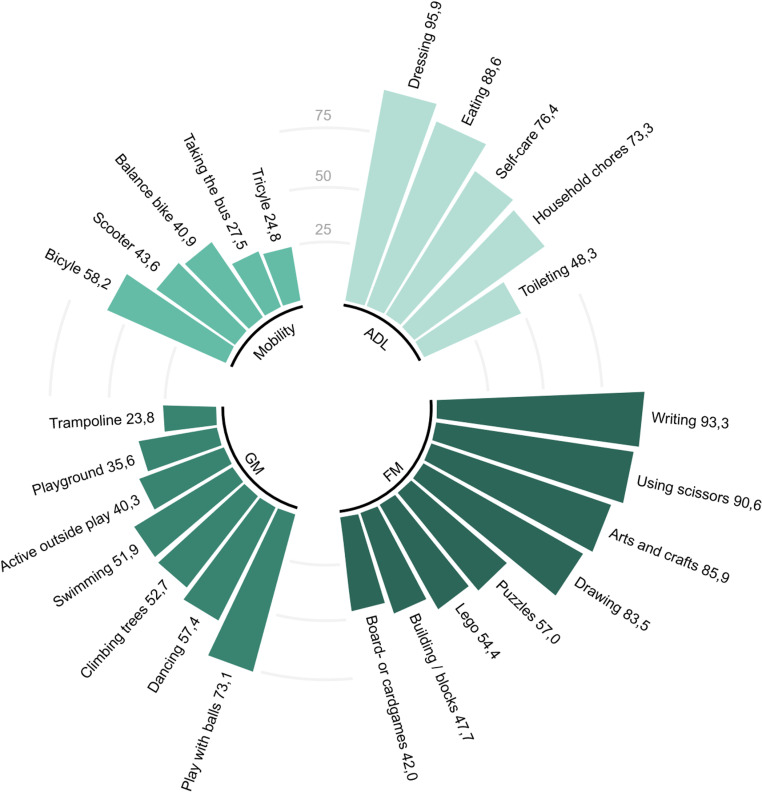
Parents reported that their children encountered difficulty with various activities (%) (n = **491).** Abbreviations: ADL, activities of daily living; FM, fine motor activities, GM, gross motor activities.

Parents reported that their children experienced challenges in various activities of daily living, fine and gross motor skills, and mobility (encompassing aspects such as biking and using public transportation) (Fig 5). Approximately half of the parents reported to encounter difficulties with toilet training (n = 235, 47.9%), including faecal incontinence (soiling or encopresis) beyond 4y (n = 118, 24.0%), daytime urinary incontinence after 5y (n = 137, 27.9%), and bedwetting (enuresis) after 5y (n = 196, 39.9%). None of these outcomes were significantly different in the presence of co-occurring conditions (χ² 0.0–3.0, *p* = 0.08–0.88, S2 File). Qualitative analysis (n = 234) disclosed that delayed and/or prolonged toilet training was a common concern among these parents. Parents reported that issues with toilet training would reoccur in response to stress or environmental changes, such as holidays or transitions to a new school year. Motor skill difficulties (e.g., undressing, aiming, wiping), behavioural challenges (e.g., concentration, procrastination, refusal to go to the toilet), and sensory issues (e.g., poor body awareness, difficulty relaxing, preference for wet wipes) were commonly cited by parents as contributing factors to these difficulties. A mother of a six-year-old child described her child's struggles with toilet training as: “He didn't know how to hold it, he peed next to the toilet (he still does). He didn't know how to aim, and he still doesn't know how to wipe his bottom properly.”

Half of the children were reported to have reduced sleep quality (n = 248, 50.6%) which was observed to a greater extent in children with co-occurring conditions (40.0% vs. 57.9%, χ² = 15.2, p < 0.001). Qualitative analyses indicated that parents (n = 248) mostly cited difficulties in their children falling asleep, often attributing this to worries: “[He is] a real thinker who has difficulty catching sleep. His head is often full, and he doesn't find rest”. Sleep disturbances were frequently associated with parasomnia, such as nightmares, sleepwalking, and sleep talking, with occasional reports of sleep-related anxiety.

#### Speech articulation.

Around half of the parents (n = 257, 52.3%) reported concerns regarding their children's speech articulation with more worries in children with co-occurring conditions (44.5% versus 57.7%; χ² = 8.3, p = 0.004). Parents (n = 250; 50.9%) reported issues relating to unclear articulation, including phonetic and phonological errors, such as difficulties with specific sound pronunciation, syllable flipping, and word distortion or mispronunciation. Additionally, suprasegmental issues, such as speaking too quickly, fluency problems like stuttering, as well as alterations in voice volume, were frequently mentioned. These issues were sometimes associated with feelings of insecurity: “She tends to speak unclear, quiet, and mumbles. This is usually because she feels insecure. However, she can articulate clearly, for example when she is reading.” Moreover, oral motor challenges, including hypotonia and tongue positioning problems, were reported, along with drooling and a tendency to keep the mouth open.

### School and education

#### School trajectory and enjoyment.

Most children attended regular education, with 78.8% (n = 387) attending mainstream schools and 3.7% (n = 18) alternative didactic schools (E.g., Steiner, Montessori, Freinet). Special education was attended by 13.0% (n = 64) and a minority attended inclusive regular education (n = 15, 3.1%). Only seven children (1.4%) received other types of schooling (homeschooling, private schooling, no schooling, or schooling tailored to gifted children). Nearly one in four children had to retake at least one year of school (n = 114, 23.2%). This proportion was significantly larger in children with co-occurring conditions (16.5% versus 27.8%; χ² = 8.5, p = 0.003). Qualitative analysis (n = 114) indicated that children often repeated the final year of preschool (usually around 5y), primarily due to being considered immature or lacking the necessary motor skills for a successful transition to primary school while in older children, general delays in scholastic skills (reading, language, math) were reasons for repeating a year of school. Parents had mixed feelings about repeating a year of school: “He had a teacher with very little understanding [of his problems] and who also told him he was stupid and couldn't do anything. His self-confidence was completely gone so [we took him to a] new school […] to give him some peace of mind”.

According to parents, one in five children (n = 93, 18.9%) did not at all enjoy going to school. Qualitative analysis (n = 188) indicated that children experienced increased stress to perform well at school while perceiving that they are slower and ‘different from others’: “He gives his maximum to achieve the minimum”. Furthermore, parents reported incidences of bullying, challenges in connecting with peers, a lack of understanding, and increased fatigue among their children. Parents expressed concerns about their child's exhaustion at the end of the school day, noting that tasks take longer to complete, learning becomes more difficult, and there is limited time for relaxation. The majority of parents (n = 373, 76.0%) reported greater fatigue in their child compared to peers by the end of the school day and this proportion was even higher in children with co-occurring conditions (70.0% versus 79.4%; χ² = 4.6, p = 0.03).

#### Communication and support at school.

The majority of teachers were informed about the child's movement difficulties at the start of the school year (n = 410, 83.5%) and parent meetings were held either only with the class teacher (n = 129, 26.3%) or with a multidisciplinary team (n = 261, 53.2%) to discuss the child's needs in the classroom. At the beginning of the school year, contact between the children's therapist and the teachers was made for approximately half of the children (n = 237, 48.3%). According to parents, adaptations were implemented by teachers for 59.9% (n = 294) of children, often including extra time for assessments or exams (n = 248, 50.5%). Additional support was provided by the school’s learning support teacher (n = 147, 29.9%) and/or an external support teacher (n = 229, 46.6%). Regarding PE classes, 39.4% (n = 190) of parents were informed that their child received some form of support. Furthermore, 28.0% (n = 138) of children did not feel confident participating in occasional sports events at school, with no significant difference observed based on the presence of co-occurring conditions (χ² = 3.9, p = 0.149; S2 File).

#### Therapy and intervention.

Most children received therapy (n = 438, 89.2%) with physiotherapy, speech-language therapy, and psychotherapy most frequently reported (Table 3). Around one in three children (n = 143, 29.1%) occasionally missed classes due to therapy attendance. While more than half of the parents felt that their child received sufficient therapy to address their movement difficulties (n = 281, 57.2%), they also expressed that they, as parents, did not feel adequately supported to assist their child with these challenges (n = 290, 59.1%). One in ten children (n = 45, 9.2%) took medication, primarily for increasing focus and attention related to a co-occurring ADHD diagnosis.

**Table 3:** Overview of therapy attendance history, sector of therapy attendance, and current therapy attendance a month.

History of therapy attendance (%)			Sector of therapy attendance (%)				Current therapy attendance a month (hours)			
	Yes	No	Missing	Private	CAR	Other	Missing	Mean ± SD	Range	N*
Physiotherapy	78.0	8.1	13.8	69.2	15.1	1.8	13.8	3.6 ± 2.1	[0.5–12]	142/196
Speech-language therapy	59.9	22.4	17.7	62.9	19.4	4.1	13.6	3.8 ± 2.0	[0.5–10]	88/133
Psychology	34.0	43.0	23.0	58.1	22.8	4.8	14.4	1.9 ± 1.2	[0.16–5]	39/62
Occupational therapy	29.9	46.2	23.8	38.1	40.8	8.8	12.2	4.3 ± 3.6	[0.5–25]	53/83
Neuropsychology	15.1	59.9	25.1	58.1	16.2	10.8	14.9	2.0 ± 1.3	[0.5–4.0]	15/28
Psychomotor therapy	10.2	81.9	7.9	76.0	4.0	2.0	18.0	2.8 ± 1.2	[1.0–4.0]	8/11
Other	14.3	29.9	55.8	65.7	7.1	18.6	8.6	3.1 ± 3.4	[0.25–12]	19/24

*Due to incomplete responses to these specific questions, the number of respondents was expressed relative to the total number of respondents currently attending that therapy.

Abbreviations: CAR, Centre Ambulatory Rehabilitation; PT, physiotherapy; OT, occupational therapy, SLT, speech-language therapy.

### Social-emotional impact

**Fig 6:**
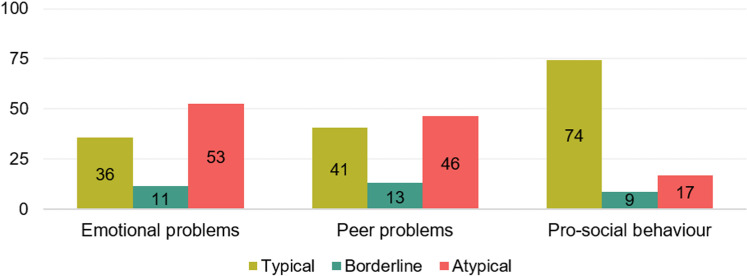
Distribution of performance (%) on three subtests of the Strengths and Difficulties Questionnaire (n = **491): About half of the children experience clinical ranges of emotional and peer challenges though the majority demonstrates adequate pro-social behaviour.**

On the SDQ, approximately half of the children exhibited elevated levels of parent-reported emotional challenges (n = 259, 52.7%) and peer-related issues (n = 228, 46.4%) whilst the majority demonstrated pro-social behaviour within the typical range (n = 365, 74.3%) (Fig 6). Additionally, half of the parents reported that their child had difficulties making friends (n = 221, 45.0%). All of these variables were significantly more prevalent in children with co-occurring conditions, with more emotional challenges (57.8% versus 68.5%, U = 24197.0, p = 0.001, *r* = −0.168), more peer-related issues (48.7% versus 66.8%; U = 22311.0, p < 0.001, *r* = −0.233), more difficulties making friends (33.0% versus 53.3%; χ² = 19.7, p < 0.001), and lower levels of pro-social behaviour (22.1% versus 28.1%; U = 33293.5, p = 0.006, *r* = 0.144).

### Impact on the family

**Fig 7:**
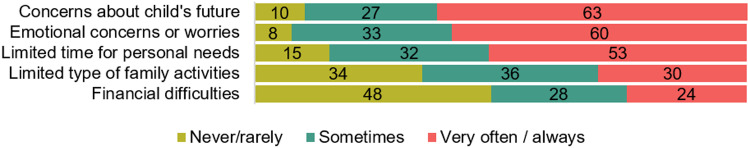
The multifaceted impact of movement difficulties on families: parents worry most about the future and emotional wellbeing of their child and perceive limited time for their own personal needs (n = **491) (%).**

More than half of the parents expressed concerns about their child's future, experienced emotional worries, found limited time for their own personal needs, and reported that the type of family activities was limited (Fig 7). Additionally, they expressed concerns of the adverse impact of motor problems on their child's academic success (n = 329, 67%) and future professional activities (n = 318, 64.8%). Parents also faced financial burden (Fig 7). Monthly out-of-pocket expenses for therapy were reported in 154 (31.4%) children. On average, these expenses amounted to €144.21 per month (range €0–771). The majority of parents received no additional financial support for therapy attendance beyond enforced health insurance coverage (n = 358, 72.9%), although 22.8% (n = 112) received increased child allowance. Many parents (n = 292, 59.5%) reported additional expenses associated with their child's movement difficulties such as adapted school materials, clothing, and private classes to learn specific motor skills. Moreover, 37.5% (n = 184) regularly took time off work to facilitate their child's therapy attendance, while 49.1% (n = 241) had either reduced their working hours or were contemplating doing so (16.7%, n = 82).

Qualitative analysis (n = 471) revealed that parents’ top priority is addressing the limited awareness and understanding of DCD, which often results in others perceiving their concerns as exaggerated. They express a need for clearer guidance on supporting their child with DCD, as well as a plea for reduced administrative burdens and assistance in navigating the complex support system. Additionally, they feel a lack of support, leaving many of them exhausted and struggling to find enough time to help their child effectively.

### Strengths of children with DCD

**Fig 8:**
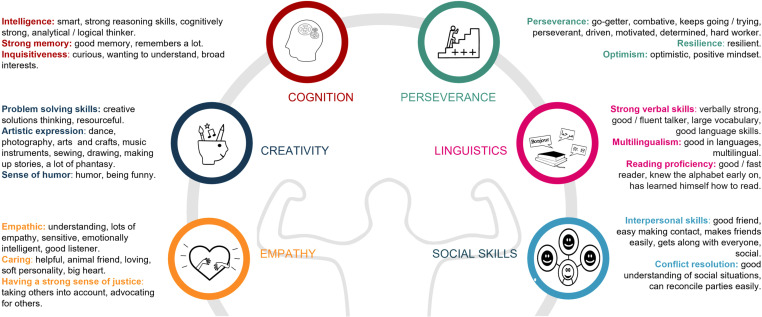
Parent-reported strengths in children with Developmental Coordination Disorder (n = **476).**

The identification of reported strengths was conducted through qualitative analysis (Fig 8). Parents (n = 476) reported their children were very empathic, caring, and had a strong sense of justice whilst creativity was observed in problem-solving skills, artistic expression, and a good sense of humour. Furthermore, they highlighted the cognitive abilities of their children, encompassing intelligence, a strong memory, and inquisitiveness. Perseverance, resilience, and optimism were also described. One parent elucidated this characteristic stating: “She tries as hard as she can to get ahead. She does everything she can to do what’s asked of her, and if she doesn't get it right [the] first time, she tries again until she succeeds.” A subset of parents emphasized their children's linguistic proficiency, including strong verbal skills, multilingualism, and reading proficiency. Lastly, good social skills were mentioned. One mother summarizes: “Our son is incredibly creative, out of the box thinking but above all very social, funny, open-minded, has a very big heart, is very empathic and can perfectly reconcile parties and disarm a difficult situation.”

## Discussion

This survey, encompassing 491 Belgian children's parental experiences, highlights the widespread impact on both the child and the family. On average, children received a diagnosis two years after their first search for help. However, one in five children did not (yet) receive a formal diagnosis for their movement difficulties. Despite efforts such as school support meetings and therapy access, parents indicate a lack of adequate support, tailored leisure activities, and general awareness, affecting the child's well-being and their own. Recognizing and addressing these challenges while acknowledging the strengths of these children is essential for ensuring a better future. It is important to acknowledge that this study lacks a control group, limiting our ability to compare these experiences with those of typically developing children.

### Diagnostic trajectory and support

Many medical professionals in Belgium are utilizing the correct diagnostic term “Developmental Coordination Disorder”, although descriptive terms such as “dyspraxia” are still in use. The international DCD diagnostic guidelines are well-known in Belgium, possibly explaining the higher adherence to correct diagnostic terminology and the slightly older age at diagnosis (6.9y) when compared to Australian (5.3y) and American results (4.9y) [6,26]. International guidelines [33] specifically recommend caution in diagnosing children before the age of five, also likely influencing the timing of diagnoses. Interestingly, the presence of co-occurring conditions did not significantly impact the age of first concerns or diagnosis. However, such conditions might redirect attention from motor difficulties, potentially delaying recognition while, conversely, multidisciplinary assessments for children with multiple challenges may expedite the identification of motor difficulties. Nonetheless, the two-year gap between seeking help and confirming a diagnosis is substantial, with unclear access to support during this period. The majority of families reported the substantial benefit of receiving a diagnosis, emphasizing the necessity of correct and timely diagnosis. Further research is warranted to explore the impact of early diagnosis on parental stress and child outcomes. In Australia and Canada [6,24], parents also played a pivotal role in identifying concerns early on, yet over half had never heard of the condition beforehand, likely delaying the first search for help. Increased awareness of DCD among parents can assist in early recognition. Finally, parents felt insufficiently supported to help their child after diagnosis. Our findings therefore highlight that efforts should be intensified to provide pre- and post-diagnostic support for parents.

Interestingly, concerns were more frequently expressed in Belgian preschool settings (39.4%) compared to Canada (8.0%), possibly attributed to the extended preschool duration of three and a half years in Belgium compared to one year in Canada [24]. Contrary to international guidelines [33], teachers were not often included in the diagnostic process. Integrating teachers into both the diagnostic and support process could facilitate timely diagnosis and enhance awareness and support for children with DCD in schools. This is necessary, as numerous children in our study had to repeat at least one academic year, a practice whose efficacy is subject to debate. The impact of academic progress from repeating might be limited [34] and although short-term improvements in emotional well-being are observed, long-term outcomes suggest potential negative effects on social acceptance [35]. Alternatively, Tingle, Schoeneberger and Algozzine [36] advocate for remedial instruction over grade retention, which was received by less than half of the included children in this study. However, meetings involving parents and/or therapists, as well as adaptations for the child in the classroom, occurred in approximately half of the children, demonstrating efforts to accommodate diverse learning needs. However, there was less communication between parents and PE teachers. Despite their training in motor development, the majority of PE teachers in this study were not familiar with DCD. Yet, previous literature indicates that when asked to identify children likely to perform poorly on a motor test, PE teachers correctly identified half of the children, highlighting their potential as an untapped resource for detecting features of DCD at school [37].

### Widespread challenges

In this survey, we examined the perceived challenges in children with DCD and confirmed increased social and emotional difficulties [38, 39], sleep disturbances [40–42], diminished physical activity [43], fatigue [44], toilet training issues [27,45], speech articulation challenges [46, 47], and a substantial impact on the family [4–6].

The high levels of social and emotional challenges underscore the importance of addressing these aspects, given that individuals with DCD are at an increased risk of developing depression and anxiety [48]. The results further emphasize the need for increased awareness of DCD within the mental health sector [49], as the potential presence of underlying DCD is frequently overlooked when individuals seek support [50]. While this appears to be a promising intervention target, to date, there have been no studies investigating the effectiveness of psychotherapy for individuals with DCD. The cause and nature of the reported lower sleep quality in this study requires further research as the reported prevalence of 50.6% in this study is higher than seen in typically developing children (3–36%) [51, 52]. While in individuals with ASD and ADHD, sleep issues have been associated with factors such as melatonin regulation dysfunction, obstructive sleep apnoea, and circadian rhythm sleep disorder [53], it might also be related to lower emotional well-being leading to increased pre-sleep worries [54]. Astoundingly, 82.5% of children in our study sample did not meet the recommended 60 minutes of daily moderate-to-vigorous physical activity, which is over four times higher than the 19.6% reported among children aged six to nine in the WHO European region [55]. These findings support international guidelines advocating for increased emphasis on physical fitness for individuals with DCD. Despite global trends indicating declining physical activity among children [55], neurodivergent children may require additional support to enjoy movement. The decreased physical activity can cause increased fatigue in these children, while fatigue can also lead to reduced physical activity. Moreover, since nearly every aspect of daily life demands motor coordination, which is particularly challenging for children with DCD, it is unsurprising that most parents report heightened fatigue in their children. To date, toilet training challenges are well-known in ASD [56] and ADHD [57], but remain largely unrecognized in DCD. The prevalence of daytime urinary incontinence (27.9%) and bedwetting (39.9%) reported after 5y in this study significantly exceeds the population prevalence of 10% [58] and 21% [59] respectively. Toileting not only involves a range of coordinated motor skills such as managing zippers, orienting to the toilet, and wiping, but also requires, among others, effective planning and organization (e.g., timing bathroom breaks during recess, ensuring access to toilet paper if not available), spatial orientation (locating the restroom), and management of sensory stimuli (feeling the urge, coping with certain sensations). Detecting these toilet training issues is crucial, as they often correlate with psychosocial and peer challenges [60] and are associated with impaired treatment response [61]. In our sample, speech articulation difficulties (52.3%) were reported in nearly four times as many children as those diagnosed with speech language conditions (13.2%). Interestingly, diagnoses of speech language conditions were less prevalent in the Belgian sample compared to other countries (23–40%) [6,24,26]. Articulation is only one aspect of the complex process of speech production that requires a lot of coordination of muscles controlling expiration and fine tuning of the sounds we make (i.e., tongue, mouth and jaw). Speech production may be impacted by self-confidence but also by oral motor coordination difficulties previously identified in children with DCD [46, 47]. Although this study did not assess the severity of the speech articulation challenges, it is crucial to acknowledge their potential to significantly and independently affect emotional well-being and overall quality of life. The above-discussed challenges stress the importance of implementing holistic and personalized approaches.

The study findings further confirm that the impact may extend beyond the individual, impacting parents’ day-to-day activities and well-being, prompting parental calls for increased awareness of DCD and guidance to support their child effectively. However, most intervention studies have predominantly focused on ‘treating’ the child with DCD. To date, no formal DCD parent support programs (e.g., including psychoeducation) have been studied, although they have been proven beneficial in other NDCs [62, 63]. Only one study compared Cognitive Orientation to Daily Occupational Performance (CO-OP) training in children with DCD, with and without parental coaching, but revealed no statistical differences between groups in terms of improvement in children's occupational and motor performance [64]. Yet, this study focused on a small group of engaged parents undergoing coaching in CO-OP strategies rather than psychoeducation and did not measure the impact on the parents themselves. In Belgium, physical and occupational therapists play a vital role in supporting the child, school, and parents, while psychologists could also play a crucial role in this process. This is especially important given the impact on the well-being of both the child and their family. Many children in the survey attended sessions with a psychologist, but it is unclear how many parents were also engaged in these services. Further research is warranted to explore whether targeted parental coaching and psychoeducation can alleviate family stress and enhance the child's outcomes as well as the parent’s well-being.

### Parent-reported strengths in children

This study represents the first large-scale investigation into parent-reported strengths in children with DCD. There are similarities with strengths reported in other NDCs such as creativity, empathy, and problem-solving skills [65, 66]. The high prevalence of co-occurring conditions may have influenced these findings. Further research will need to clarify if these strengths are related specifically to DCD, to co-occurring NDCs, or to neurodivergence in general. Recognizing positive traits in neurodivergent individuals is essential within a positive psychology approach [67], as it may reduce stigma, promote inclusion, and support individuals by utilizing their positive attributes. For individuals with DCD who are susceptible to low self-esteem, identifying their strengths, and enhancing their utilization may offer a valuable strategy for boosting mental well-being.

### Study limitations and strengths

This study lacked a comparison group of typically developing children or those with other NDCs, precluding direct comparisons. However, it sheds light on the specific areas requiring attention within a large cohort of children. Although the study design did not allow for diagnosis verification, this approach allowed the inclusion of all children, regardless of whether they had received a formal diagnosis. Due to the survey’s length and the prerequisite literacy skills in Dutch or French, families who speak other languages or possess lower literacy levels might have been prevented from participation. Given the extensive data already reported, further comparisons by potential influencing variables such as sex, age, socio-economic status, and geographical regions of Belgium will be subject of further analysis.

## Conclusion

These study results clearly highlight the widespread and significant impact of DCD from the parents’ perspective across multiple domains. We need to support children not only in their motor skills but also in other developmental domains (e.g., continence, sleep, speech articulation) and contexts (e.g., school and leisure activities), with particular emphasis on emotional well-being. Moreover, several strengths are reported by parents that can be utilized to boost children's confidence and overall development. The results emphasize that parents are insufficiently supported, with their own mental health also being negatively affected. Moreover, there is an urgent need to increase awareness among schools, the general population, and healthcare professionals. This is essential to ensure they can recognize the features of DCD, understand its associated challenges, and provide appropriate support. The next crucial step involves investigating how to better empower and support parents, raise awareness more broadly, and engage in discussions with policymakers and stakeholders to collaboratively devise effective strategies to overcome these challenges.

## Supporting information

S1. File Full survey Dutch/French. (PDF)

S2. File Percentage distribution of non-significant variables comparing between the presence of the absence of co-occurring conditions. (PDF)

S3 File Manuscript_Dutch_translation. (PDF)

S4 File Manuscript_French_translation. (PDF)

## References

[1] American Psychiatric Assocation. Diagnostic and Statistical Manual of Mental Disorders (5th text revision edition)(DSM-5-TR). Washington; 2022.

[2] Zwicker JG, Harris SR, Klassen AF. Quality of life domains affected in children with developmental coordination disorder: a systematic review. Child Care Health Dev. 2013;39(4):562–80. doi: 10.1111/j.1365-2214.2012.01379.x 22515477

[3] Missiuna C, Moll S, King S, King G, Law M. A trajectory of troubles: parents’ impressions of the impact of developmental coordination disorder. Phys Occup Ther Pediatr. 2007;27(1):81–101. doi: 10.1300/j006v27n01_06 17298942

[4] Cleaton MAM, Lorgelly PK, Kirby A. Developmental coordination disorder: the impact on the family. Qual Life Res. 2019;28(4):925–34. doi: 10.1007/s11136-018-2075-1 30536221

[5] Jijon AM, Leonard HC. Parenting stress in parents of children with developmental coordination disorder. Res Dev Disabil. 2020;104:103695. doi: 10.1016/j.ridd.2020.103695 32474232

[6] Licari MK, Alvares GA, Bernie C, Elliott C, Evans KL, McIntyre S, et al. The unmet clinical needs of children with developmental coordination disorder. Pediatr Res. 2021;90(4):826–31. doi: 10.1038/s41390-021-01373-1 33504966

[7] Miller LT, Missiuna CA, Macnab JJ, Malloy-Miller T, Polatajko HJ. Clinical description of children with developmental coordination disorder. Can J Occup Ther. 2001;68(1):5–15. doi: 10.1177/000841740106800101 11233688

[8] Farmer M, Echenne B, Bentourkia M. Study of clinical characteristics in young subjects with Developmental coordination disorder. Brain Dev. 2016;38(6):538–47. doi: 10.1016/j.braindev.2015.12.010 26763621

[9] Flapper BC, Schoemaker MM. Developmental coordination disorder in children with specific language impairment: co-morbidity and impact on quality of life. Res Dev Disabil. 2013;34(2):756–63. 23220052 10.1016/j.ridd.2012.10.014

[10] Missiuna C, Cairney J, Pollock N, Campbell W, Russell DJ, Macdonald K, et al. Psychological distress in children with developmental coordination disorder and attention-deficit hyperactivity disorder. Res Dev Disabil. 2014;35(5):1198–207. 24559609 10.1016/j.ridd.2014.01.007

[11] Meachon E, Melching H, Alpers G. The Overlooked Disorder: (Un)awareness of developmental coordination disorder across clinical professions. Adv Neurodev Disord. 2023;8:253–61.

[12] Karkling M, Paul A, Zwicker J. Occupational therapists’ awareness of guidelines for assessment and diagnosis of developmental coordination disorder: Mesure selon laquelle les ergotherapeutes connaissent les lignes directrices relatives a l’evaluation et au diagnostic du trouble du developpement de la coordination. Can. J. Occup. Ther. 2017;84(3):148–57. 28730904 10.1177/0008417417700915

[13] Maciver D, Owen C, Flannery K, Forsyth K, Howden S, Shepherd C, et al. Services for children with developmental co-ordination disorder: the experiences of parents. Child Care Health Dev. 2011;37(3):422–9. doi: 10.1111/j.1365-2214.2010.01197.x 21276034

[14] Ahern K. Developmental coordination disorder: validation of a qualitative analysis using statistical factor analysis. Int. J. Qual. Methods. 2002;1(3):70–82. doi: 10.1177/160940690200100305

[15] Alonso Soriano C, Hill EL, Crane L. Surveying parental experiences of receiving a diagnosis of developmental coordination disorder (DCD). Res Dev Disabil. 2015;43–44:11–20. doi: 10.1016/j.ridd.2015.06.001 26151439

[16] Wilson BN, Neil K, Kamps PH, Babcock S. Awareness and knowledge of developmental co-ordination disorder among physicians, teachers and parents. Child Care Health Dev. 2013;39(2):296–300. doi: 10.1111/j.1365-2214.2012.01403.x 22823542 PMC3579234

[17] Hunt J, Zwicker JG, Godecke E, Raynor A. Awareness and knowledge of developmental coordination disorder: A survey of caregivers, teachers, allied health professionals and medical professionals in Australia. Child Care Health Dev. 2021;47(2):174–83. doi: 10.1111/cch.12824 33140459 PMC7894302

[18] Khairati F, Stewart N, Zwicker J. How developmental coordination disorder affects daily life: The adolescent perspective. Res. Dev. Disabil. 2024;144:104640. 38056031 10.1016/j.ridd.2023.104640

[19] Rodger S, Mandich A. Getting the run around: accessing services for children with developmental co-ordination disorder. Child Care Health Dev. 2005;31(4):449–57. doi: 10.1111/j.1365-2214.2005.00524.x 15948882

[20] Novak C, Lingam R, Coad J, Emond A. “Providing more scaffolding”: parenting a child with developmental co-ordination disorder, a hidden disability. Child Care Health Dev. 2012;38(6):829–35. doi: 10.1111/j.1365-2214.2011.01302.x 21848938

[21] Mancini V, Licari M, Alvares G, McQueen M, McIntyre S, Reynolds J. Psychosocial wellbeing, parental concerns, and familial impact of children with developmental coordination disorder. Res. Dev. Disabil. 2024;145:104659. doi: 10.1016/j.ridd.2024.104659 38160588

[22] Reynolds JE, Alvares GA, Williams J, Froude E, Elliott C, McIntyre S, et al. Investigating the impact of developmental coordination difficulties across home, school, and community settings: Findings from the Australian Impact for DCD survey. Res Dev Disabil. 2024;147:104712. doi: 10.1016/j.ridd.2024.104712 38471296

[23] Klein ES, Cheung C, Garces A, Barbic S, Zwicker JG. Caregiver burden and mental health: Parent perspectives when raising a child with developmental coordination disorder. Res Dev Disabil. 2024;144:104656. doi: 10.1016/j.ridd.2023.104656 38141380

[24] Klein ES, Licari M, Barbic S, Zwicker JG. Diagnostic services for developmental coordination disorder: Gaps and opportunities identified by parents. Child Care Health Dev. 2024;50(1):e13230. doi: 10.1111/cch.13230 38265129

[25] Klein ES, Licari M, Barbic S, Zwicker JG. Success or failure? Are we meeting the needs of children with developmental coordination disorder? CJOT. 2023;91(2):00084174231197618. 10.1177/00084174231197618 PMC11088219 37670671

[26] Tamplain P, Miller HL, Peavy D, Cermak S, Williams J, Licari M. The impact for DCD - USA study: The current state of Developmental Coordination Disorder (DCD) in the United States of America. Res Dev Disabil. 2024;145:104658. doi: 10.1016/j.ridd.2023.104658 38176290 PMC10840388

[27] De Roubaix A, Van de Velde D, Van Waelvelde H. Parental report of early features of developmental coordination disorder: A qualitative study. Res Dev Disabil. 2023;143:104636. doi: 10.1016/j.ridd.2023.104636 37980836

[28] Beaton DE, Bombardier C, Guillemin F, Ferraz MB. Guidelines for the process of cross-cultural adaptation of self-report measures. Spine (Phila Pa 1976). 2000;25(24):3186–91. doi: 10.1097/00007632-200012150-00014 11124735

[29] Goodman R. The strengths and difficulties questionnaire: a research note. J Child Psychol Psychiatry. 1997;38(5):581–6. doi: 10.1111/j.1469-7610.1997.tb01545.x 9255702

[30] Harris PA, Taylor R, Minor BL, Elliott V, Fernandez M, O’Neal L, et al. The REDCap consortium: Building an international community of software platform partners. J Biomed Inform. 2019;95:103208. doi: 10.1016/j.jbi.2019.103208 31078660 PMC7254481

[31] Team J. JASP (version 0.14. 1). 2020.

[32] Lumivero. Nvivo Software 14.24.4 (49). 2024.

[33] Blank R, Barnett AL, Cairney J, Green D, Kirby A, Polatajko H, et al. International clinical practice recommendations on the definition, diagnosis, assessment, intervention, and psychosocial aspects of developmental coordination disorder. Dev Med Child Neurol. 2019;61(3):242–85. doi: 10.1111/dmcn.14132 30671947 PMC6850610

[34] Goos M, Pipa J, Peixoto F. Effectiveness of grade retention: A systematic review and meta-analysis. Educ Res Rev. 2021;34:100401.

[35] Wu W, West SG, Hughes JN. Effect of grade retention in first grade on psychosocial outcomes. J Educ Psychol. 2010;102(1):135–52. doi: 10.1037/a0016664 20448829 PMC2864494

[36] Tingle LR, Schoeneberger J, Algozzine B. Does grade retention make a difference? The Clearing House 2012;85(5):179-85.

[37] Piek JP, Edwards K. The identification of children with developmental coordination disorder by class and physical education teachers. Br J Educ Psychol. 1997;67 (Pt 1):55–67. doi: 10.1111/j.2044-8279.1997.tb01227.x 9114732

[38] De Roubaix A, Roeyers H, Van Waelvelde H, Bar-On L. Social responsiveness in children with developmental coordination disorder. Braz J Phys Ther. 2024;28(1):100591. doi: 10.1016/j.bjpt.2024.100591 38394720 PMC10899025

[39] Lingam R, Jongmans MJ, Ellis M, Hunt LP, Golding J, Emond A. Mental health difficulties in children with developmental coordination disorder. Pediatrics. 2012;129(4):e882-91. doi: 10.1542/peds.2011-1556 22451706

[40] Barnett AL, Wiggs L. Sleep behaviour in children with developmental co-ordination disorder. Child Care Health Dev. 2012;38(3):403–11. doi: 10.1111/j.1365-2214.2011.01260.x 21668466

[41] Chénier-Leduc G, Béliveau M-J, Dubois-Comtois K, Butler B, Berthiaume C, Pennestri M-H. Sleep difficulties in preschoolers with psychiatric diagnoses. Int J Environ Res Public Health. 2019;16(22):4485. doi: 10.3390/ijerph16224485 31739470 PMC6888178

[42] Wiggs K, Elmore AL, Nigg JT, Nikolas MA. Pre- and perinatal risk for attention-deficit hyperactivity disorder: does neuropsychological weakness explain the link?. J Abnorm Child Psychol. 2016;44(8):1473–85. doi: 10.1007/s10802-016-0142-z 26961824 PMC5362256

[43] Rivilis I, Hay J, Cairney J, Klentrou P, Liu J, Faught BE. Physical activity and fitness in children with developmental coordination disorder: a systematic review. Res Dev Disabil. 2011;32(3):894–910. doi: 10.1016/j.ridd.2011.01.017 21310588

[44] Wiggs L, Sparrowhawk M, Barnett AL. Parent report and actigraphically defined sleep in children with and without developmental coordination disorder; links with fatigue and sleepiness. Front Pediatr. 2016;4:81. doi: 10.3389/fped.2016.00081 27540540 PMC4973273

[45] Summers J, Larkin D, Dewey D. Activities of daily living in children with developmental coordination disorder: dressing, personal hygiene, and eating skills. Hum Mov Sci. 2008;27(2):215–29. doi: 10.1016/j.humov.2008.02.002 18348898

[46] Lingam R, Golding J, Jongmans MJ, Hunt LP, Ellis M, Emond A. The association between developmental coordination disorder and other developmental traits. Pediatrics. 2010;126(5):e1109-18. doi: 10.1542/peds.2009-2789 20956425

[47] Archibald LMD, Alloway TP. Comparing language profiles: children with specific language impairment and developmental coordination disorder. Int J Lang Commun Disord. 2008;43(2):165–80. doi: 10.1080/13682820701422809 17852518

[48] Omer S, Jijon AM, Leonard HC. Research Review: Internalising symptoms in developmental coordination disorder: a systematic review and meta‐analysis. J Child Psychol Psychiatry. 2018;60(6). 10.1111/jcpp.13001 PMC7379561 30485419

[49] Meachon EJ, Zemp M, Alpers GW. Developmental Coordination Disorder (DCD): Relevance for Clinical Psychologists in Europe. Clin Psychol Eur. 2022;4(2):e4165. doi: 10.32872/cpe.4165 36397944 PMC9667416

[50] Verlinden S, De Wijngaert P, Van den Eynde J. Developmental coordination disorder in adults: A case series of a condition that is underdiagnosed by adult psychiatrists. Psychiatry Research Case Reports. 2023;2(2):100148. doi: 10.1016/j.psycr.2023.100148

[51] Vélez-Galarraga R, Guillén-Grima F, Crespo-Eguílaz N, Sánchez-Carpintero R. Prevalence of sleep disorders and their relationship with core symptoms of inattention and hyperactivity in children with attention-deficit/hyperactivity disorder. Eur J Paediatr Neurol. 2016;20(6):925–37. doi: 10.1016/j.ejpn.2016.07.004 27461837

[52] Meltzer LJ, Crabtree VM. Pediatric sleep problems: A clinician’s guide to behavioral interventions. American Psychological Association; 2016.

[53] Al Lihabi A. A literature review of sleep problems and neurodevelopment disorders. Front Psychiatry. 2023;14:1122344. doi: 10.3389/fpsyt.2023.1122344 36911135 PMC9995546

[54] Bagley EJ, Kelly RJ, Buckhalt JA, El-Sheikh M. What keeps low-SES children from sleeping well: the role of presleep worries and sleep environment. Sleep Med. 2015;16(4):496–502. doi: 10.1016/j.sleep.2014.10.008 25701537 PMC4395518

[55] Whiting S, Buoncristiano M, Gelius P, Abu-Omar K, Pattison M, Hyska J. Physical activity, screen time, and sleep duration of children aged 6–9 years in 25 countries: an analysis within the WHO European childhood obesity surveillance initiative (COSI) 2015–2017. Obes facts. 2021;14(1). 10.1159/000511263 PMC7983588 33352575

[56] Leader G, Francis K, Mannion A, Chen J. Toileting problems in children and adolescents with parent-reported diagnoses of autism spectrum disorder. J Dev Phys Disabil. 2018;30:307-27.

[57] McKeown C, Hisle-Gorman E, Eide M, Gorman GH, Nylund CM. Association of constipation and fecal incontinence with attention-deficit/hyperactivity disorder. Pediatrics. 2013;132(5):e1210-5. doi: 10.1542/peds.2013-1580 24144702 PMC4530301

[58] Nieuwhof-Leppink AJ, Schroeder RPJ, van de Putte EM, de Jong TPVM, Schappin R. Daytime urinary incontinence in children and adolescents. Lancet Child Adolesc Health. 2019;3(7):492–501. doi: 10.1016/S2352-4642(19)30113-0 31060913

[59] Butler RJ, Heron J. The prevalence of infrequent bedwetting and nocturnal enuresis in childhood. A large British cohort. Scand J Urol Nephrol. 2008;42(3):257–64. doi: 10.1080/00365590701748054 18432533

[60] von Gontard A, Lettgen B, Olbing H, Heiken-Löwenau C, Gaebel E, Schmitz I. Behavioural problems in children with urge incontinence and voiding postponement: a comparison of a paediatric and child psychiatric sample. Br J Urol. 1998;81 Suppl 3:100–6. doi: 10.1046/j.1464-410x.1998.00019.x 9634031

[61] O’Kelly F, t’Hoen LA, Silay S, Lammers RJM, Sforza S, Bindi E, et al. Neuropsychiatric developmental disorders in children are associated with an impaired response to treatment in bladder bowel dysfunction: a prospective multi-institutional european observational study. J Urol. 2023;210(6):899–907. doi: 10.1097/JU.0000000000003701 37747130

[62] Tonge B, Brereton A, Kiomall M, Mackinnon A, King N, Rinehart N. Effects on parental mental health of an education and skills training program for parents of young children with autism: a randomized controlled trial. J Am Acad Child Adolesc Psychiatry. 2006;45(5):561–9. doi: 10.1097/01.chi.0000205701.48324.26 16670650

[63] Nussey C, Pistrang N, Murphy T. How does psychoeducation help? A review of the effects of providing information about Tourette syndrome and attention-deficit/hyperactivity disorder. Child Care Health Dev. 2013;39(5):617–27. doi: 10.1111/cch.12039 23461278

[64] Araujo CRS, Cardoso AA, Polatajko HJ, de Castro Magalhães L. Efficacy of the Cognitive Orientation to daily Occupational Performance (CO-OP) approach with and without parental coaching on activity and participation for children with developmental coordination disorder: A randomized clinical trial. Res Dev Disabil. 2021;110:103862. doi: 10.1016/j.ridd.2021.103862 33508735

[65] Maw KJ, Beattie G, Burns EJ. Cognitive strengths in neurodevelopmental disorders, conditions and differences: A critical review. Neuropsychologia. 2024;197:108850. doi: 10.1016/j.neuropsychologia.2024.108850 38467371

[66] Schippers LM, Horstman LI, van de Velde H, Pereira RR, Zinkstok J, Mostert JC, et al. A qualitative and quantitative study of self-reported positive characteristics of individuals with ADHD. Front Psychiatry. 2022;13:922788. doi: 10.3389/fpsyt.2022.922788 36311492 PMC9597197

[67] Seligman LD, Ollendick TH, Langley AK, Baldacci HB. The utility of measures of child and adolescent anxiety: a meta-analytic review of the Revised Children’s Manifest Anxiety Scale, the State-Trait Anxiety Inventory for Children, and the Child Behavior Checklist. J Clin Child Adolesc Psychol. 2004;33(3):557–65. doi: 10.1207/s15374424jccp3303_13 15271613

