# Supplementary material for: Understanding the impact of developmental coordination disorder on Belgian children and families: A national survey study
Source: PLoS One. 2025 Apr 22;20(4):e0320311. doi: 10.1371/journal.pone.0320311 (PMC12013903; doi:10.1371/journal.pone.0320311)
Supplement: S1. File — (PDF) [file pone.0320311.s001.pdf]

Codebook

Data Dictionary Codebook

2022-08-17 15:24:27

Collapse all instruments

#	Variable / Field Name	Field Label *Field Note*	Field Attributes (Field Type, Validation, Choices, Calculations, etc.)
Instrument: **Introductie** (introductie) Enabled as survey			Collapse
1	record_id	Record ID	text
2	inleiding	In de kindertijd worden belangrijke vaardigheden verworven zoals leren stappen, praten, eten met bestek, lopen en spelen. Jammer genoeg is dit niet voor elk kind even gemakkelijk. Wanneer kinderen langdurig moeilijkheden ervaren tijdens het leren en uitvoeren van deze vaardigheden én dit een grote impact heeft op hun dagelijks functioneren, kan dit een indicatie zijn voor de diagnose Developmental Coordination Disorder (DCD). Als ouder of voogd van een kind met deze aanhoudende bewegingsmoeilijkheden staat u voor heel wat uitdagingen. Zo moet u bijvoorbeeld de weg vinden tot onderzoek, therapie en onderwijsondersteuning. Dit kan gepaard gaan met een grote fysieke, mentale en financiële impact op uw kind en uw gezin. Spijtig genoeg is het bewustzijn van DCD én de impact die het heeft op kinderen en hun gezin momenteel erg beperkt in België. Hier moet verandering in komen! Om dit te kunnen verwezenlijken hebben wij uw hulp nodig. We nodigen u uit om deel te nemen aan deze enquête als u een of meerdere kind(eren) heeft tussen 4 en 18 jaar met aanhoudende bewegingsmoeilijkheden die niet geassocieerd zijn aan andere motorische aandoeningen (bvb. cerebrale parese, spierziekte,...). Ook wanneer er (nog) geen diagnose van DCD werd gesteld, maar er wel duidelijke aanhoudende bewegingsmoeilijkheden ervaren worden, mag u de vragenlijst invullen. Dit project, onder leiding van de Universiteit van Gent, zal gebruikt worden om essentieel bewijsmateriaal te verzamelen om de impact van DCD aan te tonen. Door dit te doen hopen we om het bewustzijn te verhogen en een actie te kunnen starten om betere ondersteuning te voorzien voor families, scholen en medische centra.Deze enquête bevat een reeks vragen en neemt ongeveer 30 tot 45 minuten in beslag. U kan uw antwoorden tijdelijk opslaan en op een later tijdstip verder gaan met het beantwoorden van de vragen. Deelnemen aan deze enquête is volledig vrijwillig en anoniem. Mogelijks liggen sommige onderwerpen wat gevoelig. Deze informatie isechter zeer belangrijk om uw ervaring volledig te kunnen begrijpen. Wij verzoeken u vriendelijk om uw tijd te nemen om de vragen te beantwoorden en om de vragenlijst door te sturen naar alle andere Belgische families die u kent waar ook bewegingsmoeilijkheden worden opgemerkt.	descriptive
3	consent	Ik heb bovenstaande informatie over de studie gelezen en begrepen en ga akkoord om deel te nemen aan de studie door te klikken naar de volgende pagina.	radio, Required<br>1 Ja<br>0 Nee<br><br>Custom alignment: LV
4	introductie_complete	Section Header: *Form Status*<br>Complete?	dropdown<br>0 Incomplete<br>1 Unverified<br>2 Complete
Instrument: **Inclusiecriteria** (inclusiecriteria) Enabled as survey			Collapse
5	incl_belg	Mijn kind woont in België.	radio, Required<br>1 Ja<br>0 Nee
6	incl_leeftijd	Mijn kind is tussen 4 en 18 jaar oud.	radio, Required<br>1 Ja<br>0 Nee
7	incl_moeilijkheden	Mijn kind ondervindt bewegingsmoeilijkheden en/of er is een vermoeden of diagnose van DCD.	radio, Required<br>1 Ja<br>0 Nee

8	incl_medische_aand	Mijn kind heeft geen andere medische aandoening die de bewegingsmoeilijkheden verklaren (bijvoorbeeld cerebrale parese, spierziekte,...).	radio, Required<br>1 Ja<br>0 Nee
9	inclusiecriteria_complete	Section Header: *Form Status*<br>Complete?	dropdown<br>0 Incomplete<br>1 Unverified<br>2 Complete
Instrument: **Familie** (familie) Enabled as survey			Collapse
10	fam_n_kind	Hoeveel kinderen (inclusief pluskinderen) heeft u?	radio, Required<br>1 1<br>2 2<br>3 3<br>4 4<br>5 5+
11	fam_n_kind_mot	Hoeveel van uw kinderen ondervinden bewegingsmoeilijkheden?	radio, Required<br>1 1<br>2 2<br>3 3<br>4 4<br>5 5+
12	familiaal_mot	Is er een geschiedenis van bewegingsmoeilijkheden in uw familie?<br>*(Dit gaat niet over het kind zelf)*	radio, Required<br>1 Ja<br>0 Nee
13	fambew<br>Show the field ONLY if:<br>[familiaal_mot] = '1'	Wie ondervindt er binnen uw familie nog bewegingsmoeilijkheden en welke moeilijkheden ervaren ze?	notes<br>Custom alignment: LV
14	familiaal_other	Is er een geschiedenis van andere medische aandoeningen in uw familie die een effect kunnen hebben op beweging/ leren? (dit gaat niet over het kind zelf)<br>*(dit gaat niet over het kind zelf)*	radio, Required<br>1 Ja<br>0 Nee
15	fam_other<br>Show the field ONLY if:<br>[familiaal_other] = '1'	Gelieve aan te kruisen wat van toepassing is:	checkbox, Required<br>1 fam_other___1 AD(H)D (Attention Deficit Hyperactivity Disorder)<br>2 fam_other___2 Autisme spectrum stoornis (ASS)<br>3 fam_other___3 Leerstoornissen (vb. dyslexie, dyscalculie, dysorthografie)<br>4 fam_other___4 Verstandelijke beperking<br>5 fam_other___5 Globale ontwikkelingsvertraging<br>6 fam_other___6 Tic aandoening<br>7 fam_other___7 Andere {fam_other_other}<br>Custom alignment: LV
16	fam_other_other<br>Show the field ONLY if:<br>[fam_other(7)] = '1'		text
17	uitleg	Aangezien de impact kan variëren van kind tot kind willen we uw vriendelijk verzoeken om eerst de enquête te vervolledigen voor uw oudste kind met bewegingsmoeilijkheden. Na afloop worden de vragen herhaald voor elk volgend kind met bewegingsmoeilijkheden.	descriptive
18	familie_complete	Section Header: *Form Status*<br>Complete?	dropdown<br>0 Incomplete<br>1 Unverified<br>2 Complete
Instrument: **Impact DCD** (impact_dcd) Enabled as survey			Collapse
19	age	Section Header: *Domein 1: bewegingsmoeilijkheden en diagnose*<br>Wat is de huidige leeftijd van uw kind?	text, Required

20	sex	Wat is het biologische geslacht van uw kind?	radio, Required<br>1 Man<br>2 Vrouw<br>3 Andere
21	n_child	Welke plaats heeft het kind in het gezin?	radio, Required<br>1 Oudste<br>2 Midden (niet het oudste of het jongste)<br>3 Jongste<br>4 Enig kind
22	ga	Na hoeveel weken zwangerschap werd uw kind geboren?<br>*ter info: typische zwangerschapsduur is 40.0 weken*	text (number_1dp, Min: 24.0, Max: 45.0), Required
23	complicaties	Waren er complicaties voor, tijdens of na de bevalling?	radio, Required<br>1 Ja<br>0 Nee
24	complicaties_specifiek<br>Show the field ONLY if: [complicaties] = '1'	Welke?	notes<br>Custom alignment: LV
25	age_worry	Hoe oud was uw kind toen u zich voor het eerst zorgen maakte over zijn/haar bewegen?	text, Required
26	omgeving_geobserveerd	In welke omgeving of door wie werden deze moeilijkheden voor het eerst geobserveerd of aangekaart?	checkbox, Required<br>1 omgeving_geobserveerd___1 Thuis<br>2 omgeving_geobserveerd___2 Bij grootouders<br>3 omgeving_geobserveerd___3 Huisarts<br>4 omgeving_geobserveerd___4 Kind en Gezin<br>5 omgeving_geobserveerd___5 Onthaalmoeder/crèch<br>6 omgeving_geobserveerd___6 Kinderopvang/ naschoolse opvang<br>7 omgeving_geobserveerd___7 Kleuterschool<br>8 omgeving_geobserveerd___8 Lagere school<br>9 omgeving_geobserveerd___9 Middelbare school<br>10 omgeving_geobserveerd___10 Sportclub<br>11 omgeving_geobserveerd___11 CLB (centrum voor leerlingenbegeleiding<br>12 omgeving_geobserveerd___12 Andere {omgeving_andere}<br>Custom alignment: LV
27	omgeving_andere<br>Show the field ONLY if: [omgeving_geobserveerd(12)] = '1'		text
28	age_help	Hoe oud was uw kind toen u voor het eerst professionele hulp zocht?	text, Required
29	diagnose	Is er een formele diagnose gesteld voor de bewegingsmoeilijkheden van uw kind?	radio, Required<br>1 Ja<br>0 Nee
30	diagnose_specifiek<br>Show the field ONLY if: [diagnose] = '1'	Is er bij uw kind een formele diagnose gesteld van een van de volgende motorische aandoeningen? (gelieve alle antwoorden aan te duiden die van toepassing zijn)	checkbox, Required<br>1 diagnose_specifiek___1 Developmental coordination disorder (DCD) of coördinatie ontwikkelingstoornis<br>2 diagnose_specifiek___2 Dyspraxie<br>3 diagnose_specifiek___3 Sensorische integratiestoornis<br>4 diagnose_specifiek___4 Minimal Brain Damage<br>5 diagnose_specifiek___5 Hypotonie<br>6 diagnose_specifiek___6 Hypermobiliteit<br>7 diagnose_specifiek___7 Andere {diagnose_andere}<br>Custom alignment: LV

31	diagnose_andere<br>Show the field ONLY if:<br>[diagnose_specifiek(7)] = '1'		text
32	diagnose_professional<br>Show the field ONLY if:<br>[diagnose] = '1'	Welke professional stelde deze diagnose? (gelieve alle antwoorden aan te duiden die van toepassing zijn)	checkbox, Required<br>1 diagnose_professional___1 Huisarts<br>2 diagnose_professional___2 Kinderarts<br>3 diagnose_professional___3 Ergotherapeut<br>4 diagnose_professional___4 Kinesitherapeut<br>5 diagnose_professional___5 COS (centrum voor ontwikkelingsstoornisse<br>6 diagnose_professional___6 CAR (centrum voor ambulante revalidatie)<br>7 diagnose_professional___7 (kinder)neuroloog<br>8 diagnose_professional___8 (kinder)psychiater<br>9 diagnose_professional___9 CLB-arts (centrum voor leerlingenbegeleiding)<br>10 diagnose_professional___10 Andere {d_aother}<br>Custom alignment: LV
33	d_aother<br>Show the field ONLY if:<br>[diagnose_professional(10)] = '1'		text
34	uitleg7<br>Show the field ONLY if:<br>[diagnose] = '1'	Werd de diagnose gebaseerd op volgende zaken?	descriptive
35	diagn_ervaring_ouder<br>Show the field ONLY if:<br>[diagnose] = '1'	Uw ervaringen als ouder	radio (Matrix), Required<br>1 Ja<br>0 Nee<br>2 Ik weet het niet
36	diagn_ervaring_leerkracht<br>Show the field ONLY if:<br>[diagnose] = '1'	De ervaring van de leerkracht van uw kind	radio (Matrix), Required<br>1 Ja<br>0 Nee<br>2 Ik weet het niet
37	diagn_motorische_test<br>Show the field ONLY if:<br>[diagnose] = '1'	Motorische test	radio (Matrix), Required<br>1 Ja<br>0 Nee<br>2 Ik weet het niet
38	diagn_neuro_ond<br>Show the field ONLY if:<br>[diagnose] = '1'	Neurologisch onderzoek	radio (Matrix), Required<br>1 Ja<br>0 Nee<br>2 Ik weet het niet
39	diagn_iq<br>Show the field ONLY if:<br>[diagnose] = '1'	IQ-test	radio (Matrix), Required<br>1 Ja<br>0 Nee<br>2 Ik weet het niet
40	diagn_logo<br>Show the field ONLY if:<br>[diagnose] = '1'	Logopedische test	radio (Matrix), Required<br>1 Ja<br>0 Nee<br>2 Ik weet het niet
41	diagn_hersenscan<br>Show the field ONLY if:<br>[diagnose] = '1'	Hersenscan (MR, CT,...)	radio (Matrix), Required<br>1 Ja<br>0 Nee<br>2 Ik weet het niet

42	diagn_eeg<br>Show the field ONLY if:<br>[diagnose] = '1'	EEG (Elektro-encefalogram)	radio (Matrix), Required<br>1 Ja<br>0 Nee<br>2 Ik weet het niet
43	diagn_ander<br>Show the field ONLY if:<br>[diagnose] = '1'	Andere {diagn_argumenten_andere}	radio (Matrix)<br>1 Ja<br>0 Nee<br>2 Ik weet het niet
44	diagn_argumenten_andere<br>Show the field ONLY if:<br>[diagn_ander] = '1'		notes
45	leeftijd_diagnose<br>Show the field ONLY if:<br>[diagnose] = '1'	Hoe oud was uw kind toen hij/zij de diagnose kreeg?	text, Required
46	diagnose_gekend<br>Show the field ONLY if:<br>[diagnose] = '1'	Had u voor de diagnose al gehoord van de aandoening?	radio, Required<br>1 Ja<br>0 Nee
47	diagnose_gekend_hoe<br>Show the field ONLY if:<br>[diagnose_gekend] = '1'	Hoe kende u de diagnose?	notes, Required<br>Custom alignment: LV
48	help<br>Show the field ONLY if:<br>[diagnose] = '1'	Heeft het krijgen van deze diagnose u als ouder geholpen?	radio, Required<br>1 Ja<br>0 Nee
49	help_uitleg<br>Show the field ONLY if:<br>[diagnose] = '1'	Waarom wel/niet?	notes<br>Custom alignment: LV
50	diagnose_geholpen_kind<br>Show the field ONLY if:<br>[diagnose] = '1'	Heeft het krijgen van deze diagnose uw kind geholpen?	radio, Required<br>1 Ja<br>0 Nee
51	diagnose_geholpen_kind_extra<br>Show the field ONLY if:<br>[diagnose] = '1'	Waarom wel/niet?	notes<br>Custom alignment: LV
52	info<br>Show the field ONLY if:<br>[diagnose] = '1'	Waar kon u bij aanvang terecht voor informatie en ondersteuning? (Gelieve zo volledig mogelijk te zijn)	notes<br>Custom alignment: LV
53	info_belangrijkst<br>Show the field ONLY if:<br>[diagnose] = '1'	Wat is/was uw belangrijkste bron van informatie over de diagnose?	notes<br>Custom alignment: LV
54	uitleg6<br>Show the field ONLY if:<br>[diagnose] = '1'	Kenden de volgende personen de diagnose wanneer u hierover sprak?	descriptive
55	huisarts<br>Show the field ONLY if:<br>[diagnose] = '1'	Huisarts	radio (Matrix), Required<br>1 Ja<br>0 Nee<br>2 Nvt
56	pediater<br>Show the field ONLY if:<br>[diagnose] = '1'	Kinderarts/ Pediater	radio (Matrix), Required<br>1 Ja<br>0 Nee<br>2 Nvt
57	neuroloog<br>Show the field ONLY if:<br>[diagnose] = '1'	Neuroloog	radio (Matrix), Required<br>1 Ja<br>0 Nee<br>2 Nvt
58	psychiater<br>Show the field ONLY if:<br>[diagnose] = '1'	Psychiater	radio (Matrix), Required<br>1 Ja<br>0 Nee<br>2 Nvt

59	kinesitherapeut<br>Show the field ONLY if:<br>[diagnose] = '1'	Kinesitherapeut	radio (Matrix), Required<br>1 Ja<br>0 Nee<br>2 Nvt
60	klasleerkracht<br>Show the field ONLY if:<br>[diagnose] = '1'	Klasleerkracht	radio (Matrix), Required<br>1 Ja<br>0 Nee<br>2 Nvt
61	zorgleerkracht<br>Show the field ONLY if:<br>[diagnose] = '1'	Zorgleerkracht	radio (Matrix), Required<br>1 Ja<br>0 Nee<br>2 Nvt
62	turnleerkracht<br>Show the field ONLY if:<br>[diagnose] = '1'	Turnleerkracht	radio (Matrix), Required<br>1 Ja<br>0 Nee<br>2 Nvt
63	sportclub<br>Show the field ONLY if:<br>[diagnose] = '1'	Sportclub	radio (Matrix), Required<br>1 Ja<br>0 Nee<br>2 Nvt
64	familie<br>Show the field ONLY if:<br>[diagnose] = '1'	Familie	radio (Matrix), Required<br>1 Ja<br>0 Nee<br>2 Nvt
65	vrienden<br>Show the field ONLY if:<br>[diagnose] = '1'	Vrienden	radio (Matrix), Required<br>1 Ja<br>0 Nee<br>2 Nvt
66	beschrijving<br>Show the field ONLY if:<br>[diagnose] = '0'	Zijn de bewegingsmoeilijkheden van uw kind als volgt 'beschreven' door een professional? (gelieve alle antwoorden aan te duiden die van toepassing zijn)	checkbox, Required<br>1 beschrijving___1 Risico / kenmerken / vermoeden van DCD<br>2 beschrijving___2 Motorische planningsmoeilijkheden<br>3 beschrijving___3 Problemen met motorische coördinatie<br>4 beschrijving___4 Houterige motoriek<br>5 beschrijving___5 Onhandig<br>6 beschrijving___6 Vertraagde motorische ontwikkeling<br>7 beschrijving___7 Vertraagde motorische mijlpalen<br>8 beschrijving___8 Reflexintegratiestoornis<br>9 beschrijving___9 Andere {beschrijving_ander}<br>10 beschrijving___10 De bewegingsmoeilijkheden werden niet beschreven door een professional.<br>Custom alignment: LV
67	beschrijving_ander<br>Show the field ONLY if:<br>[beschrijving(9)] = '1'		text

68	beschrijving_door<br>Show the field ONLY if: [beschrijving(1)] = '1' or [beschrijving(2)] = '1' or [beschrijving(3)] = '1' or [beschrijving(4)] = '1' or [beschrijving(5)] = '1' or [beschrijving(6)] = '1' or [beschrijving(7)] = '1' or [beschrijving(8)] = '1' or [beschrijving(9)] = '1'	Welke professional beschreef dit zo? (gelieve alle antwoorden aan te duiden die van toepassing zijn)	checkbox, Required<br>1 beschrijving_door___1 Huisarts<br>2 beschrijving_door___2 Kinderarts / pediater<br>3 beschrijving_door___3 Ergotherapeut<br>4 beschrijving_door___4 Kinesitherapeut<br>5 beschrijving_door___5 COS (centrum voor ontwikkelingsstoornissen)<br>6 beschrijving_door___6 CAR (centrum voor ambulante revalidatie)<br>7 beschrijving_door___7 (kinder)neuroloog<br>8 beschrijving_door___8 (kinder)psychiater<br>9 beschrijving_door___9 CLB-arts (centrum voor leerlingenbegeleiding)<br>10 beschrijving_door___10 Psycholoog<br>11 beschrijving_door___11 Logopedist<br>12 beschrijving_door___12 Osteopaat<br>13 beschrijving_door___13 Andere {beschrijven_door_andere}<br>Custom alignment: LV
69	beschrijven_door_andere<br>Show the field ONLY if: [beschrijving_door(13)] = '1'		text
70	leeftijd_beschrijving<br>Show the field ONLY if: [beschrijving(1)] = '1' or [beschrijving(2)] = '1' or [beschrijving(3)] = '1' or [beschrijving(4)] = '1' or [beschrijving(5)] = '1' or [beschrijving(6)] = '1' or [beschrijving(7)] = '1' or [beschrijving(8)] = '1' or [beschrijving(9)] = '1'	Hoe oud was uw kind toen hij/zij op deze manier beschreven werd?	text, Required<br>Custom alignment: LV
71	ondersteuning	Waar vond u informatie en ondersteuning?	text<br>Custom alignment: LV
72	info_geen_diagn	Wat is/was uw voornaamste bron van informatie?	text<br>Custom alignment: LV
73	ondersteuning_gn_diagn	Wist u waar u terecht kon voor ondersteuning van uw kind zijn bewegingsmoeilijkheden?	radio, Required<br>1 Ja<br>0 Nee
74	diagnostiek	Wist u waar u terecht kon voor diagnostiek van uw kind zijn bewegingsmoeilijkheden?	radio, Required<br>1 Ja<br>0 Nee
75	comorbyn	Is uw kind ook gediagnosticeerd met een bijkomstige aandoening?	radio, Required<br>1 Ja<br>2 Nee

76	andere_s<br><br>Show the field ONLY if:<br>[comorbyn] = '1'	Gelieve alle antwoorden aan te duiden die van toepassing zijn.	checkbox, Required<br>1 andere_s___1 ASS<br>2 andere_s___2 AD(H)D<br>3 andere_s___3 Verbale dyspraxie / verbale apraxie/ taalstoornis/ spreekmoeilijkheden<br>4 andere_s___4 Dysfasie<br>5 andere_s___5 Dysgrafie<br>6 andere_s___6 Leerstoornis (dyscalculie, dyslexie, dysorthografie,...)<br>7 andere_s___7 Epilepsie<br>8 andere_s___8 Angststoornis<br>9 andere_s___9 Depressie<br>10 andere_s___10 Genetische aandoening {genetisch}<br>11 andere_s___11 Andere {andere_fam}<br><br>Custom alignment: LV
77	genetisch<br><br>Show the field ONLY if:<br>[andere_s(10)] = '1'		text<br>Custom alignment: LV
78	andere_fam<br><br>Show the field ONLY if:<br>[andere_s(11)] = '1'		text
79	uitleg2	Section Header: *Domein 2: Activiteiten*<br>Waren/zijn de volgende activiteiten moeilijker voor uw kind?	descriptive
80	aankleden	Aankleden (bv. knopen, ritsen, veters)	radio (Matrix), Required<br>1 Ja<br>0 Nee<br>2 Niet van toepassing
81	eten	Eten (bv. bestek gebruiken, drank(je) inschenken)	radio (Matrix), Required<br>1 Ja<br>0 Nee<br>2 Niet van toepassing
82	zelfzorg	Zelfzorg (bv. haar wassen, tanden poetsen)	radio (Matrix), Required<br>1 Ja<br>0 Nee<br>2 Niet van toepassing
83	huishouden	Helpen met huishoudelijke taken	radio (Matrix), Required<br>1 Ja<br>0 Nee<br>2 Niet van toepassing
84	zelfstandig_toilet	Zelfstandig naar toilet gaan	radio (Matrix), Required<br>1 Ja<br>0 Nee<br>2 Niet van toepassing
85	tekenen	Tekenen	radio (Matrix), Required<br>1 Ja<br>0 Nee<br>2 Niet van toepassing
86	schrijven	Schrijven	radio (Matrix), Required<br>1 Ja<br>0 Nee<br>2 Niet van toepassing

87	knippen	Knippen	radio (Matrix), Required<br>1 Ja<br>0 Nee<br>2 Niet van toepassing
88	knutselen	Knutselen	radio (Matrix), Required<br>1 Ja<br>0 Nee<br>2 Niet van toepassing
89	lego	Spelen met lego	radio (Matrix), Required<br>1 Ja<br>0 Nee<br>2 Niet van toepassing
90	bouwen	Bouwen/ met blokken spelen	radio (Matrix), Required<br>1 Ja<br>0 Nee<br>2 Niet van toepassing
91	puzzels	Puzzels maken	radio (Matrix), Required<br>1 Ja<br>0 Nee<br>2 Niet van toepassing
92	bord_kaartspelen	Bord- of kaartspelen	radio (Matrix), Required<br>1 Ja<br>0 Nee<br>2 Niet van toepassing
93	buitenspelen	Actief buitenspelen	radio (Matrix), Required<br>1 Ja<br>0 Nee<br>2 Niet van toepassing
94	steppen	Steppen	radio (Matrix), Required<br>1 Ja<br>0 Nee<br>2 Niet van toepassing
95	trampoline	Trampoline springen	radio (Matrix), Required<br>1 Ja<br>0 Nee<br>2 Niet van toepassing
96	spelen_met_bal	Spelen met een bal	radio (Matrix), Required<br>1 Ja<br>0 Nee<br>2 Niet van toepassing
97	klimmen	Klimmen in een boom	radio (Matrix), Required<br>1 Ja<br>0 Nee<br>2 Niet van toepassing
98	dansen	Dansen	radio (Matrix), Required<br>1 Ja<br>0 Nee<br>2 Niet van toepassing
99	speeltuin	Spelen op de speeltuin	radio (Matrix), Required<br>1 Ja<br>0 Nee<br>2 Niet van toepassing

100	loopfiets	Fietsen met een loopfiets	radio (Matrix), Required<br>1 Ja<br>0 Nee<br>2 Niet van toepassing
101	driewieler	Fietsen met een driewieler	radio (Matrix), Required<br>1 Ja<br>0 Nee<br>2 Niet van toepassing
102	tweewieler	Fietsen met een tweewieler	radio (Matrix), Required<br>1 Ja<br>0 Nee<br>2 Niet van toepassing
103	zelfstandig_bus	Zelfstandig de bus nemen	radio (Matrix), Required<br>1 Ja<br>0 Nee<br>2 Niet van toepassing
104	zwemmen	Veilig zwemmen zonder toezicht	radio (Matrix), Required<br>1 Ja<br>0 Nee<br>2 Niet van toepassing
105	andere	Andere {andere_activiteiten}	radio (Matrix), Required<br>1 Ja<br>0 Nee<br>2 Niet van toepassing
106	andere_activiteiten<br>Show the field ONLY if: [andere] = '1'		text
107	beschrijving_beweging	Welke termen beschrijven het best hoe uw kind momenteel beweegt? (gelieve alle antwoorden aan te duiden die van toepassing zijn)	checkbox<br>1 beschrijving_beweging___1 Ongecoördineerd<br>2 beschrijving_beweging___2 Raar<br>3 beschrijving_beweging___3 Niet georganiseerd<br>4 beschrijving_beweging___4 Slecht getimed<br>5 beschrijving_beweging___5 Onhandig<br>6 beschrijving_beweging___6 Onstabiel<br>7 beschrijving_beweging___7 Traag<br>8 beschrijving_beweging___8 Gehaast<br>9 beschrijving_beweging___9 Zwaar<br>10 beschrijving_beweging___10 Lomp<br>11 beschrijving_beweging___11 Houterig<br>12 beschrijving_beweging___12 Andere {andere2}<br>Custom alignment: LV
108	andere2<br>Show the field ONLY if: [beschrijving_beweging(12)] = '1'		text
109	langer	Denkt u dat uw kind er langer over doet dan andere kinderen om moeilijke bewegingstaken te volbrengen?	radio<br>1 Ja<br>0 Nee
110	vermoeid	Wordt uw kind vermoeid wanneer hij/zij bewegingstaken uitvoert die hij/zij moeilijk vindt?	radio<br>1 Ja<br>0 Nee
111	meer_vermoeid	Denkt u dat uw kind meer vermoeid is dan andere kinderen op het einde van de dag?	radio<br>1 Ja<br>0 Nee
112	uren_slaap	Hoeveel uren slaapt uw kind gemiddeld per nacht?	text

113	slaapkwaliteit	Heeft uw kind een verminderde slaapkwaliteit (wordt 's nachts vaak wakker, heeft moeite met in slaap vallen, veel nachtmerries, slaapwandelen...)	radio<br>1 Ja<br>0 Nee
114	uitleg_slaapkwaliteit<br>Show the field ONLY if: [slaapkwaliteit] = '1'	Kan u hier wat meer uitleg over geven?	notes, Required<br>Custom alignment: LV
115	zelf_keuze_thuis	Als uw kind thuis 30 minuten voor zichzelf had, wat zou hij/zij dan kiezen om te doen ?	notes, Required<br>Custom alignment: LV
116	schermtijd	Hoeveel minuten schermtijd (tv, laptop, gsm, gameconsole) heeft uw kind gemiddeld per dag ter ontspanning (klasopdrachten niet meegerekend)?	text, Required
117	genieten_sport_fa	Geniet uw kind van deelnemen aan georganiseerde sportactiviteiten en fysieke activiteit?	radio<br>1 Ja<br>0 Nee<br>2 Soms
118	verduidelijking_genieten<br>Show the field ONLY if: [genieten_sport_fa] = '0' or [genieten_sport_fa] = '2'	Gelieve uw antwoord te verduidelijken.	notes<br>Custom alignment: LV
119	georg_vrije_tijd	Neemt uw kind deel aan georganiseerde vrije tijdsactiviteiten? Dit kan zowel sport, spel als muziek, tekenen, jeugdbeweging,... zijn.	radio, Required<br>1 Ja<br>0 Nee
120	uitleg_vrije_tijd<br>Show the field ONLY if: [georg_vrije_tijd] = '1'	Welke georganiseerde sportactiviteit? Hoeveel minuten per week? {sport1} {sport1_week} {sport2} {sport2_week} {sport3} {sport3_week} {sport4} {sport4_week}	descriptive
121	sport1		text
122	sport2		text
123	sport3		text
124	sport4		text
125	sport1_week		text
126	sport2_week		text
127	sport3_week		text
128	sport4_week		text
129	vrije_tijd_beschikbaar	Heeft u het gevoel dat er voldoende vrijetijdsactiviteiten beschikbaar op maat van uw kind in uw buurt?	radio, Required<br>1 Ja<br>0 Nee
130	mvpa	Hoeveel minuten per dag houdt uw kind zich bezig met gematigde tot zware fysieke activiteit (De ademhaling versnelt, het hart gaat sneller slaan, maar het kind is niet buiten adem en kan nog gewoon praten)?	radio, Required<br>1 Minder dan 30 minuten<br>2 30 minuten tot 1 uur<br>3 1 tot 2 uur<br>4 Meer dan 2 uur
131	bezorgd_fys_gez	Heeft u bezorgdheden over de impact die de bewegingsmoeilijkheden van uw kind heeft op zijn/haar fysieke gezondheid?	radio, Required<br>1 Ja<br>0 Nee
132	uitleg_bezorgd_fys_gez<br>Show the field ONLY if: [bezorgd_fys_gez] = '1'	Wat is uw grootste bezorgdheid?	notes<br>Custom alignment: LV
133	moeilijkheden_zindelijkheid	Heeft uw kind moeilijkheden ervaren met potjestraining en/of zindelijkheid?	radio, Required<br>1 Ja<br>0 Nee
134	uitleg_moeilijkheden_zindelijkheid<br>Show the field ONLY if: [moeilijkheden_zindelijkheid] = '1'	Indien ja, kan u wat meer uitleg geven over bovenstaand antwoord aub?	notes<br>Custom alignment: LV
135	uitleg_continetie	Zijn de volgende uitspraken van toepassing bij uw kind?	descriptive

136	stoelgangverlies	Vanaf de leeftijd van 4 jaar had mijn kind geen onvoorzien stoelgangverlies meer.	radio (Matrix), Required<br>1 Waar<br>0 Niet waar
137	urineverlies_overdag	Vanaf de leeftijd van 5 jaar was mijn kind altijd volledig droog overdag (ook geen druppelverlies).	radio (Matrix), Required<br>1 Waar<br>0 Niet waar
138	urineverlies_nacht	Vanaf de leeftijd van 5 jaar was mijn kind altijd volledig droog 's nachts (ook geen druppelverlies).	radio (Matrix), Required<br>1 Waar<br>0 Niet waar
139	articulatie	Werd er ooit gezegd dat uw kind een minder goede articulatie had?	radio, Required<br>1 Ja<br>0 Nee
140	uitleg_articulatie<br>Show the field ONLY if:<br>[articulatie] = '1'	Kan u hier wat meer uitleg over geven aub?	notes<br>Custom alignment: LV
141	leerjaar	Section Header: *Domein 3: School*<br>In welk leerjaar zit uw kind?	text, Required
142	soort_onderwijs	Gaat uw kind naar het regulier onderwijs, het bijzonder onderwijs of een methodeschool?	radio, Required<br>1 Regulier onderwijs<br>2 Bijzonder onderwijs type {bijzonder_onderwijs}<br>3 Inclusief onderwijs<br>4 Methodeschool (Montessori, Steiner, Freinet)<br>5 Andere {andere_soort_onderwijs}
143	andere_soort_onderwijs<br>Show the field ONLY if:<br>[soort_onderwijs] = '5'		text
144	bijzonder_onderwijs<br>Show the field ONLY if:<br>[soort_onderwijs] = '2'		text
145	jaar_overdoen	Heeft uw kind ooit een jaar op school moeten overdoen?	radio, Required<br>1 Ja<br>0 Nee
146	waarom_jaar_overdoen<br>Show the field ONLY if:<br>[jaar_overdoen] = '1'	Waarom heeft hij/zij een jaar moeten overdoen?	notes<br>Custom alignment: LV
147	leerkracht_op_hoogte	Was de leerkracht in het begin van dit schooljaar op de hoogte van de bewegingsmoeilijkheden van uw kind?	radio, Required<br>1 Ja<br>0 Nee<br>2 Ik weet het niet
148	overleg_leerkracht	Heeft u in het begin van dit schooljaar een overleg gehad met de leerkracht om de noden van uw kind te bespreken?	radio, Required<br>1 Ja, enkel met de leerkracht<br>2 Ja, met de leerkracht en andere therapeuten (multidisciplinair overleg of MDO)<br>0 Neen<br>3 Ik weet het niet
149	leerkracht_therapeut	Heeft de leerkracht in het begin van dit schooljaar contact gehad met een therapeut die uw kind begeleidt?	radio, Required<br>1 Ja<br>0 Nee<br>2 Ik weet het niet
150	aanpassingen_klas	Werden er in het begin van het schooljaar aanpassingen in de klas gedaan door de leerkracht?	radio, Required<br>1 Ja<br>0 Nee<br>2 Ik weet het niet

151	ooit_ondersteuning_school	Heeft uw kind ooit ondersteuning ontvangen vanuit de school?	radio, Required<br>1 Ja van een extern team (ondersteuningsnetwerken, vroegere GON)<br>2 Ja van een intern team (zorgcoördinator, leerkracht)<br>0 Nee<br>3 Ik weet het niet
152	extra_tijd	Krijgt uw kind extra tijd om taken te voltooien die beïnvloed worden door zijn/haar bewegingsmoeilijkheden (bv. Toetsen of examens)?	radio, Required<br>1 Ja<br>0 Nee<br>2 Ik weet het niet
153	meer_moe_school	Is uw kind meer moe dan andere kinderen op het einde van een schooldag?	radio, Required<br>1 Ja<br>0 Nee
154	turnleerkracht_school	Heeft de school van uw kind een turnleerkracht?	radio, Required<br>1 Ja<br>0 Nee
155	turnleerkracht_comm<br>Show the field ONLY if:<br>[turnleerkracht_school] = '1'	Heeft de turnleerkracht ooit gecommuniceerd met u of de klasleerkracht over het ondersteunen van uw kind?	radio<br>1 Ja<br>0 Nee<br>2 Ik weet het niet
156	ondersteuning_turnen<br>Show the field ONLY if:<br>[turnleerkracht_school] = '1'	Heeft u het gevoel dat uw kind ondersteund wordt om deel te nemen aan de turnlessen op school?	radio<br>1 Ja<br>0 Nee<br>2 Ik weet het niet
157	sportdag<br>Show the field ONLY if:<br>[turnleerkracht_school] = '1'	Voelt uw kind zich comfortabel om deel te nemen aan de sportdag op school?	radio<br>1 Ja<br>0 Nee<br>2 Niet van toepassing
158	uitdagingen_school	Wat zijn de grootste uitdagingen voor uw kind op school?	notes, Required<br>Custom alignment: LV
159	graag_school	Gaat uw kind graag naar school?	radio, Required<br>1 Ja<br>0 Nee<br>2 Soms
160	niet_graag_school<br>Show the field ONLY if:<br>[graag_school] = '0' or [graag_school] = '2'	Kan u hier wat meer uitleg over geven?	notes<br>Custom alignment: LV
161	moeilijkheden_vrienden	Heeft uw kind moeilijkheden (gehad) om vrienden te maken op school?	radio, Required<br>1 Ja<br>0 Nee
162	neg_invloed_opleiding	Denkt u dat de bewegingsmoeilijkheden van uw kind een negatieve invloed hebben op de mogelijkheid om zijn/haar opleiding succesvol af te ronden?	radio, Required<br>1 Ja<br>0 Nee
163	opleiding_succesvol	Maakt u zich zorgen over hoe de bewegingsmoeilijkheden van uw kind hem/haar kunnen belemmeren om zijn/ haar schoolopleiding succesvol af te ronden?	radio, Required<br>1 Ja<br>0 Nee
164	grootste_bezorgdheid<br>Show the field ONLY if:<br>[opleiding_succesvol] = '1'	Wat is uw grootste bezorgdheid?	notes<br>Custom alignment: LV
165	toekomstig_werk	Maakt u zich zorgen dat de bewegingsmoeilijkheden van uw kind een invloed hebben op zijn/haar toekomstige tewerkstellingsmogelijkheden?	radio, Required<br>1 Ja<br>0 Nee

166	therapie_gevolgd	Section Header: *Domein 4: Therapie en interventie*<br>Heeft uw kind ooit therapie gevolgd om hem/haar te ondersteunen in zijn/haar bewegingsmoeilijkheden?	radio, Required<br>1 Ja<br>0 Nee
167	therapie_soort_2<br>Show the field ONLY if: [therapie_gevolgd] = '1'	Ja / Nee Waar? Hoeveel jaar? Ergotherapie {ergo_yn} {ergo_where} {ergo_duur} Kinesitherapie {kine_yn} {kine_where} {kine_duur} Psycholoog {psycho_yn} {psycho_where} {psycho_duur} Logopedie {logo_yn} {logo_where} {logo_duur} Neuropsycholoog {neuropsycho_yn} {neuropsycho_where} {neuropsycho_duur} Andere: {andere_therapie} {andere_yn} {andere_where} {andere_duur}	descriptive
168	ergo_yn		radio<br>1 Ja<br>0 Nee
169	kine_yn		radio<br>1 Ja<br>0 Nee
170	psycho_yn		radio<br>1 Ja<br>0 Nee
171	logo_yn		radio<br>1 Ja<br>0 Nee
172	neuropsycho_yn		radio<br>1 Ja<br>0 Nee
173	andere_yn		radio<br>1 Ja<br>0 Nee
174	andere_therapie<br>Show the field ONLY if: [andere_yn] = '1'	Welke andere therapie heeft uw kind gevolgd?	text
175	ergo_where<br>Show the field ONLY if: [ergo_yn] = '1'		dropdown<br>1 Prive parktijk (al dan niet multidisciplinair)<br>2 CAR (Centrum voor Ambulante Revalidatie) of Revalidatiecentrum<br>3 Andere
176	kine_where<br>Show the field ONLY if: [kine_yn] = '1'		dropdown<br>1 Prive parktijk (al dan niet multidisciplinair)<br>2 CAR (Centrum voor Ambulante Revalidatie) of Revalidatiecentrum<br>3 Andere
177	psycho_where<br>Show the field ONLY if: [psycho_yn] = '1'		dropdown<br>1 Prive parktijk (al dan niet multidisciplinair)<br>2 CAR (Centrum voor Ambulante Revalidatie) of Revalidatiecentrum<br>3 Andere
178	logo_where<br>Show the field ONLY if: [logo_yn] = '1'		dropdown<br>1 Prive parktijk (al dan niet multidisciplinair)<br>2 CAR (Centrum voor Ambulante Revalidatie) of Revalidatiecentrum<br>3 Andere
179	neuropsycho_where<br>Show the field ONLY if: [neuropsycho_yn] = '1'		dropdown<br>1 Prive parktijk (al dan niet multidisciplinair)<br>2 CAR (Centrum voor Ambulante Revalidatie) of Revalidatiecentrum<br>3 Andere

180	andere_where<br>Show the field ONLY if:<br>[andere_yn] = '1'		dropdown<br>1 Prive parktijk (al dan niet multidisciplinair)<br>2 CAR (Centrum voor Ambulante Revalidatie) of Revalidatiecentrum<br>3 Andere
181	ergo_elders<br>Show the field ONLY if:<br>[ergo_where] = '3'	Waar heeft uw kind ergotherapie gevolgd?	text
182	kine_elders<br>Show the field ONLY if:<br>[kine_where] = '3'	Waar heeft uw kind kinesitherapie gevolgd?	text
183	psycho_elders<br>Show the field ONLY if:<br>[psycho_where] = '3'	Waar heeft uw kind psychotherapie gevolgd?	text
184	logo_elders<br>Show the field ONLY if:<br>[logo_where] = '3'	Waar heeft uw kind logopedie gevolgd?	text
185	neuropsycho_elders<br>Show the field ONLY if:<br>[neuropsycho_where] = '3'	Waar heeft uw kind neuropsychologie gevolgd?	text
186	anderetherapie_elders<br>Show the field ONLY if:<br>[andere_where] = '3'	Waar heeft uw kind een andere vorm van therapie gevolgd?	text
187	ergo_duur<br>Show the field ONLY if:<br>[ergo_yn] = '1'		text
188	kine_duur<br>Show the field ONLY if:<br>[kine_yn] = '1'		text
189	psycho_duur<br>Show the field ONLY if:<br>[psycho_yn]=1		text
190	logo_duur<br>Show the field ONLY if:<br>[logo_yn] = '1'		text
191	neuropsycho_duur<br>Show the field ONLY if:<br>[neuropsycho_yn] = '1'		text
192	andere_duur<br>Show the field ONLY if:<br>[andere_yn] = '1'		text
193	reden_geen_therapie<br>Show the field ONLY if:<br>[therapie_gevolgd] = '0'	Waren er specifieke redenen waarom geen therapie werd gestart?	radio, Required<br>1 Ja<br>0 Nee
194	reden_geen_therapie_spec<br>Show the field ONLY if:<br>[reden_geen_therapie] = '1'	Welke?	notes<br>Custom alignment: LV
195	therapie_momenteel	Volgt uw kind momenteel therapie om hem/haar te ondersteunen in zijn/haar bewegingsmoeilijkheden?	radio, Required<br>1 Ja<br>0 Nee
196	reden_geen_therapie_2<br>Show the field ONLY if:<br>[therapie_momenteel] = '0'	Zijn er specifieke redenen waarom uw kind momenteel geen therapie volgt?	radio, Required<br>1 Ja<br>0 Nee
197	reden_geen_therapie_spec_2<br>Show the field ONLY if:<br>[reden_geen_therapie_2] = '1'	Welke?	notes<br>Custom alignment: LV

198	therapie_soort_3<br>Show the field ONLY if:<br>[therapie_momenteel] = '1'	Ja / Nee Waar? Gemiddeld aantal uren per maand Geschatte maandelijkse kosten uit eigen zak Ergotherapie {ergo_yn_2} {ergo_where_2} {uurergo} {kostergo} Kinesitherapie {kine_yn_2} {kine_where_2} {uurkine} {kostkine} Psycholoog {psycho_yn_2} {psycho_where_2} {uurpsycho} {kostpsycho} Logopedie {logo_yn_2} {logo_where_2} {uurlogo} {kostlogo} Neuropsychologie {neuropsycho_yn_2} {neuropsycho_where_2} {uurneuropsycho} {kostneuropsycho} Andere: {andere_therapie_2} {andere_yn_2} {andere_where_2} {uurandere} {kostandere}	descriptive
199	andere_therapie_2<br>Show the field ONLY if:<br>[andere_yn_2] = '1'	Welke andere therapie heeft uw kind gevolgd?	text
200	ergo_yn_2		radio<br>1 Ja<br>0 Nee
201	kine_yn_2		radio<br>1 Ja<br>0 Nee
202	psycho_yn_2		radio<br>1 Ja<br>0 Nee
203	logo_yn_2		radio<br>1 Ja<br>0 Nee
204	neuropsycho_yn_2		radio<br>1 Ja<br>0 Nee
205	andere_yn_2		radio<br>1 Ja<br>0 Nee
206	ergo_where_2<br>Show the field ONLY if:<br>[ergo_yn_2] = '1'		dropdown<br>1 Prive parktijk (al dan niet multidisciplinair)<br>2 CAR (Centrum voor Ambulante Revalidatie) of Revalidatiecentrum<br>3 Andere
207	kine_where_2<br>Show the field ONLY if:<br>[kine_yn_2] = '1'		dropdown<br>1 Prive parktijk (al dan niet multidisciplinair)<br>2 CAR (Centrum voor Ambulante Revalidatie) of Revalidatiecentrum<br>3 Andere
208	psycho_where_2<br>Show the field ONLY if:<br>[psycho_yn_2] = '1'		dropdown<br>1 Prive parktijk (al dan niet multidisciplinair)<br>2 CAR (Centrum voor Ambulante Revalidatie) of Revalidatiecentrum<br>3 Andere
209	logo_where_2<br>Show the field ONLY if:<br>[logo_yn_2] = '1'		dropdown<br>1 Prive parktijk (al dan niet multidisciplinair)<br>2 CAR (Centrum voor Ambulante Revalidatie) of Revalidatiecentrum<br>3 Andere
210	neuropsycho_where_2<br>Show the field ONLY if:<br>[neuropsycho_yn_2] = '1'		dropdown<br>1 Prive parktijk (al dan niet multidisciplinair)<br>2 CAR (Centrum voor Ambulante Revalidatie) of Revalidatiecentrum<br>3 Andere

211	andere_where_2<br><br>Show the field ONLY if:<br>[andere_yn_2] = '1'		dropdown<br>1 Prive parktijk (al dan niet multidisciplinair)<br>2 CAR (Centrum voor Ambulante Revalidatie) of Revalidatiecentrum<br>3 Andere
212	ergo_elders_2<br><br>Show the field ONLY if:<br>[ergo_where_2] = '3'	Waar volgt uw kind ergotherapie?	text
213	kine_elders_2<br><br>Show the field ONLY if:<br>[kine_where_2] = '3'	Waar volgt uw kind kinesitherapie?	text
214	psycho_elders_2<br><br>Show the field ONLY if:<br>[psycho_where_2] = '3'	Waar volgt uw kind psychotherapie?	text
215	logo_elders_2<br><br>Show the field ONLY if:<br>[logo_where_2] = '3'	Waar volgt uw kind logopedie?	text
216	neuropsycho_elders_2<br><br>Show the field ONLY if:<br>[neuropsycho_where_2] = '3'	Waar volgt uw kind neuropsychologie?	text
217	anderetherapie_elders_2<br><br>Show the field ONLY if:<br>[andere_where_2] = '3'	Waar volgt uw kind een andere vorm van therapie?	text
218	uurergo<br><br>Show the field ONLY if:<br>[ergo_yn_2] = '1'		text
219	uurkine<br><br>Show the field ONLY if:<br>[kine_yn_2] = '1'		text
220	uurpsycho<br><br>Show the field ONLY if:<br>[psycho_yn_2] = '1'		text
221	uurlogo<br><br>Show the field ONLY if:<br>[logo_yn_2] = '1'		text
222	uurneuropsycho<br><br>Show the field ONLY if:<br>[neuropsycho_yn_2] = '1'		text
223	uurandere<br><br>Show the field ONLY if:<br>[andere_yn_2] = '1'		text
224	kostergo<br><br>Show the field ONLY if:<br>[ergo_yn_2] = '1'		text
225	kostkine<br><br>Show the field ONLY if:<br>[kine_yn_2] = '1'		text
226	kostpsycho<br><br>Show the field ONLY if:<br>[psycho_yn_2] = '1'		text
227	kostlogo<br><br>Show the field ONLY if:<br>[logo_yn_2] = '1'		text
228	kostneuropsycho<br><br>Show the field ONLY if:<br>[neuropsycho_yn_2] = '1'		text
229	kostandere<br><br>Show the field ONLY if:<br>[andere_yn_2] = '1'		text

230	bijkomstige_kosten	Zijn er bijkomende kosten die u moet maken in het kader van de bewegingsmoeilijkheden van uw kind (vb. grotere trampoline, speciale pennen, aangepast bestek,...)	radio, Required<br>1 Ja<br>0 Nee
231	bijkomstige_kost_specifiek<br>Show the field ONLY if:<br>[bijkomstige_kosten] = '1'	Welke?	notes<br>Custom alignment: LV
232	vrij_van_werk	Moet u soms vrij nemen van uw werk om uw kind therapie te laten bijwonen?	radio, Required<br>1 Ja<br>0 Nee
233	vrij_van_werk_uren<br>Show the field ONLY if:<br>[vrij_van_werk] = '1'	Hoeveel uur per maand is dat ongeveer?	text, Required
234	minder_gaan_werken	Is een ouder minder gaan werken om uw kind met bewegingsmoeilijkheden beter te kunnen ondersteunen?	radio, Required<br>1 Ja<br>0 Nee<br>2 Nee maar dit werd overwogen
235	lessen_missen	Mist uw kind lessen op school om de therapie bij te wonen?	radio, Required<br>1 Ja<br>0 Nee
236	lessen_missen_minuten<br>Show the field ONLY if:<br>[lessen_missen] = '1'	Hoeveel minuten per week mist hij/zij?	text, Required
237	voldoende_therapie	Heeft u het gevoel dat uw kind voldoende therapie krijgt om hem/haar te helpen in zijn/haar bewegingsmoeilijkheden?	radio, Required<br>1 Ja<br>0 Nee
238	voldoende_tips	Heeft u het gevoel dat u als ouder voldoende ondersteuning en tips krijgt om uw kind te helpen met zijn bewegingsmoeilijkheden?	radio, Required<br>1 Ja<br>0 Nee
239	finaniciele_ondersteuning	Ontvangt u momenteel financiële steun voor de therapiekosten van uw kind?	radio, Required<br>0 Nee<br>1 Ja, verhoogd kindergeld<br>2 Ja een persoonlijk assistentie budget (PAB)<br>3 Ja, ander: {financiele_steun_andere}
240	financiele_steun_andere<br>Show the field ONLY if:<br>[finaniciele_ondersteuning] = '3'		text
241	medicatie	Gebruikt uw kind medicatie in het kader van zijn bewegingsmoeilijkheden?	radio, Required<br>1 Ja<br>0 Nee
242	medicatie_soort<br>Show the field ONLY if:<br>[medicatie] = '1'	Welke medicatie neemt uw kind hiervoor?	text<br>Custom alignment: LV
243	bezorgd	Section Header: *Domein 5: Sociale en emotionele impact*<br>Bent u bezorgd over de impact van de bewegingsmoeilijkheden van uw kind op zijn/haar sociale en emotionele welbevinden?	radio, Required<br>1 Ja<br>0 Nee
244	bezorgdheden_voornaamste<br>Show the field ONLY if:<br>[bezorgd]=1	Wat zijn uw voornaamste bezorgdheden?	notes<br>Custom alignment: LV
245	vermijden_bew	Trekt uw kind zich terug of vermijdt hij/zij deel te nemen aan bewegingsactiviteiten?	radio (Matrix), Required<br>4 Altijd<br>3 Vaak<br>2 Soms<br>1 Zelden<br>0 Nooit

246	angstig_f	Is uw kind angstig bij het leren of uitvoeren van bewegingsactiviteiten?	radio (Matrix), Required<br>4 Altijd<br>3 Vaak<br>2 Soms<br>1 Zelden<br>0 Nooit
247	angstig_bew	Ervaart uw kind moeilijkheden om met leeftijdsgenoten in interactie te gaan?	radio (Matrix), Required<br>4 Altijd<br>3 Vaak<br>2 Soms<br>1 Zelden<br>0 Nooit
248	vrienden_m	Heeft uw kind moeilijkheden om vrienden te maken?	radio (Matrix), Required<br>4 Altijd<br>3 Vaak<br>2 Soms<br>1 Zelden<br>0 Nooit
249	lichaamstaal	Is uw kind in staat om zijn lichaamstaal aan te passen aan een sociale situatie (oogcontact, lichaamsoriëntatie)?	radio (Matrix), Required<br>4 Altijd<br>3 Vaak<br>2 Soms<br>1 Zelden<br>0 Nooit
250	gesprek	Praat uw kind op het juiste moment tijdens een gesprek?	radio (Matrix), Required<br>4 Altijd<br>3 Vaak<br>2 Soms<br>1 Zelden<br>0 Nooit
251	stil	Blijft uw kind stil en luistert hij/zij terwijl een andere persoon aan het praten is?	radio (Matrix), Required<br>4 Altijd<br>3 Vaak<br>2 Soms<br>1 Zelden<br>0 Nooit
252	gelukkig	Hoe vaak voelt uw kind zich gelukkig?	radio (Matrix), Required<br>4 Altijd<br>3 Vaak<br>2 Soms<br>1 Zelden<br>0 Nooit
253	verdietig	Hoe vaak voelt uw kind zich verdrietig?	radio (Matrix), Required<br>4 Altijd<br>3 Vaak<br>2 Soms<br>1 Zelden<br>0 Nooit
254	boos	Hoe vaak voelt uw kind zich boos of kwaad?	radio (Matrix), Required<br>4 Altijd<br>3 Vaak<br>2 Soms<br>1 Zelden<br>0 Nooit

255	uitleg3	Wilt u aub voor iedere vraag kiezen tussen "Niet waar", "Een beetje waar", of "Zeker waar". Wilt u aub uw antwoorden baseren op het gedrag van het kind de laatste zes maanden.	descriptive
256	fysieke_klachten	Klaagt vaak over hoofdpijn, buikpijn, of misselijkheid	radio (Matrix), Required<br>0 Niet waar<br>1 Een beetje waar<br>2 Zeker waar
257	zorgen_kind	Heeft veel zorgen, lijkt vaak over dingen in te zitten	radio (Matrix), Required<br>0 Niet waar<br>1 Een beetje waar<br>2 Zeker waar
258	ongelukkig	Vaak ongelukkig, in de put of in tranen	radio (Matrix), Required<br>0 Niet waar<br>1 Een beetje waar<br>2 Zeker waar
259	nerveus	Zenuwachtig of zich vastklampend in nieuwe situaties, verliest makkelijk zelfvertrouwen	radio (Matrix), Required<br>0 Niet waar<br>1 Een beetje waar<br>2 Zeker waar
260	angstig	Voor heel veel bang, is snel angstig	radio (Matrix), Required<br>0 Niet waar<br>1 Een beetje waar<br>2 Zeker waar
261	eenzaam	Nogal op zichzelf, neigt er toe alleen te spelen	radio (Matrix), Required<br>0 Niet waar<br>1 Een beetje waar<br>2 Zeker waar
262	vriend	Heeft minstens één goede vriend of vriendin	radio (Matrix), Required<br>0 Niet waar<br>1 Een beetje waar<br>2 Zeker waar
263	leuk_gevonden	Wordt over het algemeen aardig gevonden door andere kinderen	radio (Matrix), Required<br>0 Niet waar<br>1 Een beetje waar<br>2 Zeker waar
264	pesten	Wordt getreiterd of gepest door andere kinderen	radio (Matrix), Required<br>0 Niet waar<br>1 Een beetje waar<br>2 Zeker waar
265	volwassen	Kan beter opschieten met volwassenen dan met andere kinderen	radio (Matrix), Required<br>0 Niet waar<br>1 Een beetje waar<br>2 Zeker waar
266	attent_gevoelens	Houdt rekening met gevoelens van anderen	radio (Matrix), Required<br>0 Niet waar<br>1 Een beetje waar<br>2 Zeker waar
267	delen	Deelt makkelijk met andere kinderen (bijvoorbeeld speelgoed, snoep, potloden, enz.)	radio (Matrix), Required<br>0 Niet waar<br>1 Een beetje waar<br>2 Zeker waar
268	vriendelijk	Aardig tegen jongere kinderen	radio (Matrix), Required<br>0 Niet waar<br>1 Een beetje waar<br>2 Zeker waar

269	helpen	Biedt vaak vrijwillig hulp aan anderen (ouders, leerkrachten, andere kinderen)	radio (Matrix), Required<br>0 Niet waar<br>1 Een beetje waar<br>2 Zeker waar
270	behulpzaam	Behulpzaam als iemand zich heeft bezeerd, van streek is of zich ziek voelt	radio (Matrix), Required<br>0 Niet waar<br>1 Een beetje waar<br>2 Zeker waar
271	uitleg4	Hoe vaak hebben de bewegingsmoeilijkheden van uw kind de volgende effecten voor het gezin?	descriptive
272	bezorgd_gezin	Emotionele zorgen of bezorgdheid?	radio (Matrix), Required<br>4 Altijd<br>3 Heel vaak<br>2 Soms<br>1 Zelden<br>0 Nooit
273	tijd_jezelf	Het beperken van de tijd die u heeft voor uw eigen persoonlijke behoeften?	radio (Matrix), Required<br>4 Altijd<br>3 Heel vaak<br>2 Soms<br>1 Zelden<br>0 Nooit
274	soort_activiteiten	Het beperken van de soorten activiteiten die u als gezin kunt doen?	radio (Matrix), Required<br>4 Altijd<br>3 Heel vaak<br>2 Soms<br>1 Zelden<br>0 Nooit
275	financiele_moeilijkh	Financiële moeilijkheden?	radio (Matrix), Required<br>4 Altijd<br>3 Heel vaak<br>2 Soms<br>1 Zelden<br>0 Nooit
276	bezorgd_toekomst	Bezorgdheid over zijn/haar toekomst?	radio (Matrix), Required<br>4 Altijd<br>3 Heel vaak<br>2 Soms<br>1 Zelden<br>0 Nooit
277	invloed_gezinsleden	Hebben de bewegingsmoeilijkheden van uw kind nog een andere manier invloed op het gezin?	radio<br>1 Ja<br>0 Nee
278	invloed_gezinsleden_uitleg<br>Show the field ONLY if:<br>[invloed_gezinsleden] = '1'	Op wie en op welke manier?	notes<br>Custom alignment: LV
279	sterktes_kind	Wat zijn de sterktes van uw kind?	notes<br>Custom alignment: LV
280	impact_dcd_complete	Section Header: *Form Status*<br>Complete?	dropdown<br>0 Incomplete<br>1 Unverified<br>2 Complete

Instrument: **Demografische Informatie** (demografische_informatie) Enabled as survey Collapse

281	provinc	In welke provincie woont u?	radio, Required<br>1 Oost-Vlaanderen<br>2 West-Vlaanderen<br>3 Vlaams-Brabant<br>4 Antwerpen<br>5 Limburg<br>6 Henegouwen<br>7 Luik<br>8 Luxemburg<br>9 Namen<br>10 Waals-Brabant<br>Custom alignment: LV
282	pc	Wat is uw postcode?	text, Required<br>Custom alignment: LV
283	income	Wat is uw netto gezinsinkomen per maand?	radio, Required<br>1 < 1962€/maand<br>2 1962 - 3924 €/maand<br>3 >3924 €/maand<br>4 Ik wens dit liever niet te zeggen<br>Custom alignment: LV
284	mar_state	Wat is uw burgerlijke staat?	radio, Required<br>1 Alleenstaand<br>2 Getrouwd of samenwonend<br>3 Andere: {burgerlijke_staat_ander}<br>Custom alignment: LV
285	burgerlijke_staat_ander<br>Show the field ONLY if:<br>[mar_state] = '3'		text
286	diploma	Wat is het hoogst behaalde diploma van de moeder/ ouder 1?	radio, Required<br>1 Lager onderwijs<br>2 Middelbaar onderwijs<br>3 Hoger onderwijs, korte duur (bachelor)<br>4 Hoger onderwijs, lange duur (master, doctoraat)<br>Custom alignment: LV
287	diploma_2	Wat is het hoogst behaalde diploma van de vader/ ouder 2?	radio, Required<br>1 Lager onderwijs<br>2 Middelbaar onderwijs<br>3 Hoger onderwijs, korte duur (bachelor)<br>4 Hoger onderwijs, lange duur (master, doctoraat)<br>5 Niet van toepassing (éénoudergezin)<br>Custom alignment: LV
288	respondent	Wie vult deze vragenlijst in?	radio, Required<br>1 Mama<br>2 Papa<br>3 Andere: {andere_resp}<br>Custom alignment: LV
289	andere_resp<br>Show the field ONLY if:<br>[respondent] = '3'		text<br>Custom alignment: LV
290	demografische_informatie_complete	Section Header: *Form Status*<br>Complete?	dropdown<br>0 Incomplete<br>1 Unverified<br>2 Complete

Instrument: **Slotvragen** (slotvragen) Enabled as survey Collapse

291	meer_middelen_voor	Indien er meer middelen van de overheid zouden zijn, waar wens je dat deze naartoe moeten gaan? (bvb. meer ondersteuning op school organiseren, meer financiële ondersteuning, meer psychologische hulp, ...)	notes<br>Custom alignment: LV
292	ondersteuning_prioriteit	Welke vorm van ondersteuning heeft voor u momenteel de hoogste prioriteit?	notes<br>Custom alignment: LV
293	noden_kind	Waar heeft uw kind momenteel nood aan?	notes<br>Custom alignment: LV
294	noden_ouder	Waar heeft u als ouder momenteel nood aan?	notes<br>Custom alignment: LV
295	opmerkingen	Zijn er nog andere opmerkingen of bezorgdheden die u naar voren wilt brengen?	notes<br>Custom alignment: LV
296	slotvragen_complete	Section Header: *Form Status*<br>Complete?	dropdown<br>0 Incomplete<br>1 Unverified<br>2 Complete

Codebook

Data Dictionary Codebook

2022-08-17 15:25:41

Collapse all instruments

#	Variable / Field Name	Field Label *Field Note*	Field Attributes (Field Type, Validation, Choices, Calculations, etc.)
Instrument: **Introductie** (introductie) Enabled as survey			
1	record_id	Record ID	text
2	inleiding	Pendant l'enfance, des compétences importantes sont acquises, comme apprendre à marcher, à parler, à manger avec des couverts, à courir et à jouer. Malheureusement, ce n'est pas facile pour tous les enfants. Lorsque les enfants rencontrent des difficultés à long terme dans l'apprentissage et l'exécution de ces compétences et que cela a un impact majeur sur leur fonctionnement quotidien, cela peut être une indication pour le diagnostic du trouble de la coordination du développement (TDC) ou Developmental Coordination Disorder (DCD) en Anglais. En tant que parent ou tuteur d'un enfant souffrant de ces troubles persistants du mouvement, vous devez relever de nombreux défis. Par exemple, vous devez trouver votre chemin pour accéder à la recherche, la thérapie et le soutien éducatif. Cela peut avoir un impact physique, mental et financier important sur votre enfant et votre famille. Malheureusement, la sensibilisation au TDC et à son impact sur les enfants et leurs familles est actuellement très limitée en Belgique. Cela doit changer! Pour y parvenir, nous avons besoin de votre aide. Nous vous invitons à participer à cette enquête si vous avez un ou plusieurs enfants âgés de 4 à 18 ans présentant des problèmes de mouvements persistants qui ne sont pas associés à d'autres troubles moteurs (p. ex. paralysie cérébrale, dystrophie musculaire, ...). Même si aucun diagnostic de TDC n'a (encore) été posé, mais que des problèmes de mouvements persistants sont clairement ressentis, vous pouvez remplir le questionnaire. Ce projet, dirigé par l'Université de Gand, servira à rassembler des preuves essentielles pour démontrer l'impact du TDC. Nous espérons ainsi sensibiliser le public et prendre des mesures pour mieux soutenir les familles, les écoles et les centres médicaux. Cette enquête contient une série de questions et prend environ 30 à 45 minutes à compléter. Vous pouvez enregistrer temporairement vos réponses et continuer à répondre aux questions plus tard. La participation à cette enquête est totalement volontaire et anonyme. Certains sujets peuvent être sensibles. Cependant, ces informations sont très importantes pour comprendre pleinement votre expérience. Nous vous demandons de bien vouloir prendre le temps de répondre aux questions et de transmettre le questionnaire à toute autre famille belge que vous connaissez où des problèmes de mouvements ont également été constatés.	descriptive
3	consent	J'ai lu et compris les informations ci-dessus concernant l'étude et j'accepte de participer à l'étude en cliquant sur la page suivante.	radio, Required<br>1 Oui<br>0 Non<br><br>Custom alignment: LV
4	introductie_complete	Section Header: *Form Status*<br>Complete?	dropdown<br>0 Incomplete<br>1 Unverified<br>2 Complete
Instrument: **Inclusiecriteria** (inclusiecriteria) Enabled as survey			
5	incl_belg	Mon enfant habite en Belgique.	radio, Required<br>1 Oui<br>0 Non
6	incl_leeftijd	Mon enfant a entre 4 et 18 ans.	radio, Required<br>1 Oui<br>0 Non

7	incl_moeilijkheden	Mon enfant a des difficultés liées aux mouvements et/ou il y a une suspicion ou un diagnostic de TDC, TAC (trouble d'acquisition de la coordination), Developmental Coordination Disorder ou dyspraxie.	radio, Required<br>1 Oui<br>0 Non
8	incl_medische_aand	Mon enfant ne souffre pas d'un autre problème médical qui pourrait expliquer les difficultés liées aux mouvements (par exemple, paralysie cérébrale, maladie musculaire).	radio, Required<br>1 Oui<br>0 Non
9	inclusiecriteria_complete	Section Header: *Form Status*<br>Complete?	dropdown<br>0 Incomplete<br>1 Unverified<br>2 Complete
Instrument: **Familie** (familie)		Enabled as survey	Collapse
10	fam_n_kind	Combien d'enfants avez-vous (y compris de famille recomposée) ?	radio, Required<br>1 1<br>2 2<br>3 3<br>4 4<br>5 5+
11	fam_n_kind_mot	Combien de vos enfants présentent des difficultés liées aux mouvements?	radio, Required<br>1 1<br>2 2<br>3 3<br>4 4<br>5 5+
12	familiaal_mot	Y a-t-il des antécédents de difficultés liées aux mouvements dans votre famille?<br>*(Il ne s'agit pas de l'enfant lui-même)*	radio, Required<br>1 Oui<br>0 Non
13	fambew<br>Show the field ONLY if:<br>[familiaal_mot] = '1'	Qui d'autre, dans votre famille, présente des difficultés de mouvements et de quelles difficultés s'agit-il?	notes<br>Custom alignment: LV
14	familiaal_other	Y a-t-il des antécédents de toute autre condition médicale affectant les mouvements ou les apprentissages dans votre famille ? (en dehors de votre enfant qui présente déjà des difficultés liées aux mouvements)<br>*(Il ne s'agit pas de l'enfant lui-même)*	radio, Required<br>1 Oui<br>0 Non
15	fam_other<br>Show the field ONLY if:<br>[familiaal_other] = '1'	Veuillez cocher toutes les propositions qui s'appliquent:	checkbox, Required<br>1 fam_other___1 Trouble Déficitaire de l'Attention avec / sans Hyperactivité (TDA/H)<br>2 fam_other___2 Trouble du Spectre de l'Autisme (TSA)<br>3 fam_other___3 Troubles d'apprentissage (dyslexie, dyscalculie, dysorthographie,...)<br>4 fam_other___4 Déficit intellectuel<br>5 fam_other___5 Retard développemental global<br>6 fam_other___6 Troubles de tics<br>7 fam_other___7 Autre {fam_other_other}<br>Custom alignment: LV
16	fam_other_other<br>Show the field ONLY if:<br>[fam_other(7)] = '1'		text
17	uitleg	Etant donné que l'impact est susceptible de varier d'un enfant à un autre, nous vous demandons de compléter en premier lieu le questionnaire pour le plus âgé de vos enfants affectés par des difficultés liées aux.	descriptive

18	familie_complete	Section Header: *Form Status*<br>Complete?	dropdown<br>0 Incomplete<br>1 Unverified<br>2 Complete
Instrument: **Impact DCD** (impact_dcd) Enabled as survey			Collapse
19	age	Section Header: *Domaine 1/5: Difficultés de mouvements et informations diagnostiques*<br>Quel est l'âge actuel de l'enfant?	text, Required
20	sex	Quel est le genre biologique de l'enfant?	radio, Required<br>1 Garçon<br>2 Fille<br>3 Autre
21	n_child	Quelle position occupe votre enfant dans la famille?	radio, Required<br>1 Aîné<br>2 Milieu (ni le plus vieux, ni le plus jeune)<br>3 Dernier<br>4 Enfant unique
22	ga	Après combien de semaines de grossesse votre enfant est-il né?<br>*Pour votre information : la période de gestation typique est de 40.0 semaines.*	text (number_1dp, Min: 24.0, Max: 45.0), Required
23	complicaties	Avez-vous eu des complications avant, pendant ou après l'accouchement?	radio, Required<br>1 Oui<br>0 Non
24	complicaties_specifiek<br>Show the field ONLY if:<br>[complicaties] = '1'	Veuillez spécifier.	notes<br>Custom alignment: LV
25	age_worry	Quel âge avait votre enfant lorsque vous avez commencé à vous préoccuper de ses mouvements ?	text, Required
26	omgeving_geobserveerd	Dans quel environnement ou par qui ces difficultés ont-elles été observées ou évoquées en premier?	checkbox, Required<br>1 omgeving_geobserveerd___1 Maison<br>2 omgeving_geobserveerd___2 Les grands-parents<br>3 omgeving_geobserveerd___3 Médecin généraliste<br>4 omgeving_geobserveerd___4 Office de la Naissance et de l'Enfance (ONE)<br>5 omgeving_geobserveerd___5 Garderie<br>6 omgeving_geobserveerd___6 Garde d'enfants / accueil périscolaire<br>7 omgeving_geobserveerd___7 École maternelle<br>8 omgeving_geobserveerd___8 École primaire<br>9 omgeving_geobserveerd___9 École secondaire<br>10 omgeving_geobserveerd___10 Club de sport<br>11 omgeving_geobserveerd___11 PMS (Centre Psycho-Médico-Social)<br>12 omgeving_geobserveerd___12 Autre {omgeving_andere}<br>Custom alignment: LV
27	omgeving_andere<br>Show the field ONLY if:<br>[omgeving_geobserveerd(12)] = '1'		text
28	age_help	Quel âge avait votre enfant lorsque vous avez sollicité de l'aide professionnelle pour la première fois?	text, Required
29	diagnose	Votre enfant a-t-il reçu un diagnostic formel pour ses difficultés de mouvements?	radio, Required<br>1 Oui<br>0 Non

30	diagnose_specifiek<br>Show the field ONLY if:<br>[diagnose] = '1'	Votre enfant a-t-il reçu un diagnostic formel d'une des conditions liées aux mouvements suivantes? (Veuillez cocher toutes les propositions qui s'appliquent)	checkbox, Required<br>8 | diagnose_specifiek___8 | Trouble Développemental de la Coordination (TDC)<br>9 | diagnose_specifiek___9 | Trouble d'Acquisition de la Coordination (TAC)<br>1 | diagnose_specifiek___1 | Developmental Coordination Disorder (DCD)<br>2 | diagnose_specifiek___2 | Dyspraxie<br>3 | diagnose_specifiek___3 | Trouble de l'intégration sensorielle<br>4 | diagnose_specifiek___4 | Lésions cérébrales minimes (Minimal brain damage)<br>5 | diagnose_specifiek___5 | Hypotonie<br>6 | diagnose_specifiek___6 | Hypermobilité<br>7 | diagnose_specifiek___7 | Autre {diagnose_andere}<br>Custom alignment: LV
31	diagnose_andere<br>Show the field ONLY if:<br>[diagnose_specifiek(7)] = '1'		text
32	diagnose_professional<br>Show the field ONLY if:<br>[diagnose] = '1'	Qui a émis ce diagnostic? Veuillez cocher toutes les propositions qui s'appliquent.	checkbox, Required<br>1 | diagnose_professional___1 | Médecin généraliste<br>2 | diagnose_professional___2 | Pédiatre<br>3 | diagnose_professional___3 | Ergothérapeute<br>4 | diagnose_professional___4 | Kinésithérapeute<br>11 | diagnose_professional___11 | Psychomotricien<br>6 | diagnose_professional___6 | CRA (Centre de réadaptation ambulatoire)<br>7 | diagnose_professional___7 | Neurologue ou neuropédiatre<br>8 | diagnose_professional___8 | Psychiatre ou pédopsychiatre<br>9 | diagnose_professional___9 | Médecin du PMS<br>10 | diagnose_professional___10 | Autre {d_aother}<br>Custom alignment: LV
33	d_aother<br>Show the field ONLY if:<br>[diagnose_professional(10)] = '1'		text
34	uitleg7<br>Show the field ONLY if:<br>[diagnose] = '1'	Le diagnostic était-il basé sur les éléments suivants ?	descriptive
35	diagn_ervaring_ouder<br>Show the field ONLY if:<br>[diagnose] = '1'	Vos expériences en tant que parent	radio (Matrix), Required<br>1 | Oui<br>0 | Non<br>2 | Ne sait pas
36	diagn_ervaring_leerkracht<br>Show the field ONLY if:<br>[diagnose] = '1'	• L'expérience de l'enseignant de votre enfant	radio (Matrix), Required<br>1 | Oui<br>0 | Non<br>2 | Ne sait pas
37	diagn_motorische_test<br>Show the field ONLY if:<br>[diagnose] = '1'	Test moteur	radio (Matrix), Required<br>1 | Oui<br>0 | Non<br>2 | Ne sait pas

38	diagn_neuro_ond<br>Show the field ONLY if:<br>[diagnose] = '1'	Examen neurologique	radio (Matrix), Required<br>1 Oui<br>0 Non<br>2 Ne sait pas
39	diagn_iq<br>Show the field ONLY if:<br>[diagnose] = '1'	Test de QI	radio (Matrix), Required<br>1 Oui<br>0 Non<br>2 Ne sait pas
40	diagn_logo<br>Show the field ONLY if:<br>[diagnose] = '1'	Test logopédique	radio (Matrix), Required<br>1 Oui<br>0 Non<br>2 Ne sait pas
41	diagn_hersenscan<br>Show the field ONLY if:<br>[diagnose] = '1'	Scanner cérébral (IRM, CT-scan,...)	radio (Matrix), Required<br>1 Oui<br>0 Non<br>2 Ne sait pas
42	diagn_eeg<br>Show the field ONLY if:<br>[diagnose] = '1'	EEG (électroencéphalogramme)	radio (Matrix), Required<br>1 Oui<br>0 Non<br>2 Ne sait pas
43	diagn_ander<br>Show the field ONLY if:<br>[diagnose] = '1'	Autres {diagn_argumenten_andere}	radio (Matrix)<br>1 Oui<br>0 Non<br>2 Ne sait pas
44	diagn_argumenten_andere<br>Show the field ONLY if:<br>[diagn_ander] = '1'		notes
45	leeftijd_diagnose<br>Show the field ONLY if:<br>[diagnose] = '1'	Quel âge avait votre enfant lors du diagnostic?	text, Required
46	diagnose_gekend<br>Show the field ONLY if:<br>[diagnose] = '1'	Aviez-vous déjà entendu parler du diagnostic auparavant?	radio, Required<br>1 Oui<br>0 Non
47	diagnose_gekend_hoe<br>Show the field ONLY if:<br>[diagnose_gekend] = '1'	Comment avez-vous eu connaissance du diagnostic?	notes, Required<br>Custom alignment: LV
48	help<br>Show the field ONLY if:<br>[diagnose] = '1'	Le fait de recevoir ce diagnostic vous a-t-il aidé en tant que parent?	radio, Required<br>1 Oui<br>0 Non
49	help_uitleg<br>Show the field ONLY if:<br>[diagnose] = '1'	Veuillez préciser.	notes<br>Custom alignment: LV
50	diagnose_geholpen_kind<br>Show the field ONLY if:<br>[diagnose] = '1'	Le fait de recevoir ce diagnostic a-t-il aidé votre enfant?	radio, Required<br>1 Oui<br>0 Non
51	diagnose_geholpen_kind_extra<br>Show the field ONLY if:<br>[diagnose] = '1'	Veuillez préciser.	notes<br>Custom alignment: LV
52	info<br>Show the field ONLY if:<br>[diagnose] = '1'	Vers où vous êtes-vous tourné pour bénéficier d’informations et de soutien au début? (Veuillez être aussi complet que possible).	notes<br>Custom alignment: LV
53	info_belangrijkst<br>Show the field ONLY if:<br>[diagnose] = '1'	Quelle est/a été votre principale source d’informations?	notes<br>Custom alignment: LV

54	uitleg6<br>Show the field ONLY if:<br>[diagnose] = '1'	Les personnes suivantes connaissaient-elles le diagnostic de TDC lorsque vous en avez parlé?	descriptive
55	huisarts<br>Show the field ONLY if:<br>[diagnose] = '1'	Médecin de famille	radio (Matrix), Required<br>1 Oui<br>0 Non<br>2 Non applicable
56	pediater<br>Show the field ONLY if:<br>[diagnose] = '1'	Pédiatre	radio (Matrix), Required<br>1 Oui<br>0 Non<br>2 Non applicable
57	neuroloog<br>Show the field ONLY if:<br>[diagnose] = '1'	Neurologue	radio (Matrix), Required<br>1 Oui<br>0 Non<br>2 Non applicable
58	psychiater<br>Show the field ONLY if:<br>[diagnose] = '1'	Psychiatre	radio (Matrix), Required<br>1 Oui<br>0 Non<br>2 Non applicable
59	kinesitherapeut<br>Show the field ONLY if:<br>[diagnose] = '1'	Kinésithérapeute	radio (Matrix), Required<br>1 Oui<br>0 Non<br>2 Non applicable
60	klasleerkracht<br>Show the field ONLY if:<br>[diagnose] = '1'	Enseignant	radio (Matrix), Required<br>1 Oui<br>0 Non<br>2 Non applicable
61	zorgleerkracht<br>Show the field ONLY if:<br>[diagnose] = '1'	Professeur particulier	radio (Matrix), Required<br>1 Oui<br>0 Non<br>2 Non applicable
62	turnleerkracht<br>Show the field ONLY if:<br>[diagnose] = '1'	Professeur de gymnastique	radio (Matrix), Required<br>1 Oui<br>0 Non<br>2 Non applicable
63	sportclub<br>Show the field ONLY if:<br>[diagnose] = '1'	Club sportif	radio (Matrix), Required<br>1 Oui<br>0 Non<br>2 Non applicable
64	familie<br>Show the field ONLY if:<br>[diagnose] = '1'	Famille	radio (Matrix), Required<br>1 Oui<br>0 Non<br>2 Non applicable
65	vrienden<br>Show the field ONLY if:<br>[diagnose] = '1'	Amis	radio (Matrix), Required<br>1 Oui<br>0 Non<br>2 Non applicable

66	beschrijving<br>Show the field ONLY if:<br>[diagnose] = '0'	Les difficultés de mouvements de votre enfant ont-elles été décrites par un professionnel par une des propositions suivantes? (Veuillez cocher toutes les propositions qui s'appliquent)	checkbox, Required<br>1 beschrijving___1 Risque / caractéristiques / suspicion de TDC<br>2 beschrijving___2 Difficultés de planification motrice<br>3 beschrijving___3 Problèmes de coordination motrice<br>4 beschrijving___4 Rigidité motrice<br>5 beschrijving___5 Maladroit<br>6 beschrijving___6 Retard de développement moteur<br>7 beschrijving___7 Retard dans les étapes du développement moteur<br>8 beschrijving___8 Troubles de l'intégration des réflexes<br>9 beschrijving___9 Autre {beschrijving_ander}<br>10 beschrijving___10 Non, les difficultés de mouvements n'ont pas été décrites par un professionnel.<br>Custom alignment: LV
67	beschrijving_ander<br>Show the field ONLY if:<br>[beschrijving(9)] = '1'		text
68	beschrijving_door<br>Show the field ONLY if:<br>[beschrijving(1)] = '1' or [beschrijving(2)] = '1' or [beschrijving(3)] = '1' or [beschrijving(4)] = '1' or [beschrijving(5)] = '1' or [beschrijving(6)] = '1' or [beschrijving(7)] = '1' or [beschrijving(8)] = '1' or [beschrijving(9)] = '1'	Quel professionnel vous a fourni cette description? (Veuillez cocher toutes les propositions qui s'appliquent)	checkbox, Required<br>1 beschrijving_door___1 Médecin généraliste<br>2 beschrijving_door___2 Pédiatre<br>3 beschrijving_door___3 Ergothérapeute<br>4 beschrijving_door___4 Kinésithérapeute<br>14 beschrijving_door___14 Psychomotricien<br>6 beschrijving_door___6 Centre de réadaptation ambulatoire<br>7 beschrijving_door___7 Neurologue ou neuropédiatre<br>8 beschrijving_door___8 Psychiatre ou pédopsychiatre<br>9 beschrijving_door___9 Médecin PMS<br>10 beschrijving_door___10 Psychologue<br>11 beschrijving_door___11 Logopède<br>12 beschrijving_door___12 Ostéopathe<br>13 beschrijving_door___13 Autre {beschrijven_door_andere}<br>Custom alignment: LV
69	beschrijven_door_andere<br>Show the field ONLY if:<br>[beschrijving_door(13)] = '1'		text
70	leeftijd_beschrijving<br>Show the field ONLY if:<br>[beschrijving(1)] = '1' or [beschrijving(2)] = '1' or [beschrijving(3)] = '1' or [beschrijving(4)] = '1' or [beschrijving(5)] = '1' or [beschrijving(6)] = '1' or [beschrijving(7)] = '1' or [beschrijving(8)] = '1' or [beschrijving(9)] = '1'	Quel âge avait votre enfant lors de cette description?	text, Required<br>Custom alignment: LV
71	ondersteuning	Vers où vous êtes-vous tourné(e) pour bénéficier d'informations et de soutien?	text<br>Custom alignment: LV
72	info_geen_diagn	Quelle est/a été votre principale source d'informations?	text<br>Custom alignment: LV

73	ondersteuning_gn_diagn	Saviez-vous où trouver de l'aide pour les problèmes de mouvements de votre enfant?	radio, Required<br>1 Oui<br>0 Non
74	diagnostiek	Saviez-vous où aller pour obtenir un diagnostic?	radio, Required<br>1 Oui<br>0 Non
75	comorbyn	Votre enfant a-t-il/elle reçu le diagnostic d'autres troubles associés?	radio, Required<br>1 Oui<br>0 Non
76	andere_s<br>Show the field ONLY if:<br>[comorbyn] = '1'	Veuillez cocher toutes les propositions qui s'appliquent:	checkbox, Required<br>1 andere_s___1 Trouble du Spectre de l'Autisme (TSA)<br>2 andere_s___2 Trouble Déficitaire de l'Attention avec/sans Hyperactivité (TDA/H)<br>3 andere_s___3 Dyspraxie verbale / Apraxie pédiatrique de la parole /Trouble du langage/ Difficultés de la parole<br>4 andere_s___4 Dysphasie (trouble développemental du langage)<br>5 andere_s___5 Dysgraphie<br>6 andere_s___6 Trouble de l'apprentissage (dyscalculie, dyslexie, dysorthographie,...)<br>7 andere_s___7 Épilepsie<br>9 andere_s___9 Anxiété<br>10 andere_s___10 Dépression<br>11 andere_s___11 Trouble génétique {genetisch}<br>12 andere_s___12 Autre {andere_fam}<br>Custom alignment: LV
77	genetisch<br>Show the field ONLY if:<br>[andere_s(11)] = '1'		text<br>Custom alignment: LV
78	andere_fam<br>Show the field ONLY if:<br>[andere_s(12)] = '1'		text
79	uitleg2	Section Header: *Domaine 2/5: Activités*<br>Les activités suivantes ont-elles été/seront-elles plus difficiles pour votre enfant ?	descriptive
80	aankleden	S'habiller (e.g., boutons, tirettes, lacets)	radio (Matrix), Required<br>1 Oui<br>0 Non<br>2 Non applicable
81	eten	Manger (e.g., utiliser des couverts, verser une boisson)	radio (Matrix), Required<br>1 Oui<br>0 Non<br>2 Non applicable
82	zelfzorg	Soin personnel (e.g., se brosser les cheveux, les dents)	radio (Matrix), Required<br>1 Oui<br>0 Non<br>2 Non applicable
83	huishouden	Aider pour les tâches ménagères	radio (Matrix), Required<br>1 Oui<br>0 Non<br>2 Non applicable

84	zelfstandig_toilet	Aller aux toilettes de façon autonome	radio (Matrix), Required<br>1 Oui<br>0 Non<br>2 Non applicable
85	tekenen	Dessiner	radio (Matrix), Required<br>1 Oui<br>0 Non<br>2 Non applicable
86	schrijven	Ecrire	radio (Matrix), Required<br>1 Oui<br>0 Non<br>2 Non applicable
87	knippen	Découper avec des ciseaux	radio (Matrix), Required<br>1 Oui<br>0 Non<br>2 Non applicable
88	knutselen	Activités de bricolage	radio (Matrix), Required<br>1 Oui<br>0 Non<br>2 Non applicable
89	lego	Jouer aux Lego	radio (Matrix), Required<br>1 Oui<br>0 Non<br>2 Non applicable
90	bouwen	Jeu de constructions / blocs	radio (Matrix), Required<br>1 Oui<br>0 Non<br>2 Non applicable
91	puzzels	Faire des puzzles	radio (Matrix), Required<br>1 Oui<br>0 Non<br>2 Non applicable
92	bord_kaartspelen	Jeux de société/de cartes	radio (Matrix), Required<br>1 Oui<br>0 Non<br>2 Non applicable
93	buitenspelen	Jeux actifs en extérieur	radio (Matrix), Required<br>1 Oui<br>0 Non<br>2 Non applicable
94	steppen	Rouler en trottinette	radio (Matrix), Required<br>1 Oui<br>0 Non<br>2 Non applicable
95	trampoline	Faire du trampoline	radio (Matrix), Required<br>1 Oui<br>0 Non<br>2 Non applicable
96	spelen_met_bal	Jouer avec une balle	radio (Matrix), Required<br>1 Oui<br>0 Non<br>2 Non applicable

97	klimmen	Grimper à un arbre	radio (Matrix), Required<br>1 Oui<br>0 Non<br>2 Non applicable
98	dansen	Danser	radio (Matrix), Required<br>1 Oui<br>0 Non<br>2 Non applicable
99	speeltuin	Jouer sur le terrain de jeu	radio (Matrix), Required<br>1 Oui<br>0 Non<br>2 Non applicable
100	loopfiets	Rouler avec un vélo d'équilibre	radio (Matrix), Required<br>1 Oui<br>0 Non<br>2 Non applicable
101	driewieler	Rouler avec un tricycle	radio (Matrix), Required<br>1 Oui<br>0 Non<br>2 Non applicable
102	tweewieler	Rouler à vélo	radio (Matrix), Required<br>1 Oui<br>0 Non<br>2 Non applicable
103	zelfstandig_bus	Prendre le bus de façon autonome	radio (Matrix), Required<br>1 Oui<br>0 Non<br>2 Non applicable
104	zwemmen	Nager en toute sécurité sans surveillance	radio (Matrix), Required<br>1 Oui<br>0 Non<br>2 Non applicable
105	andere	Autre {andere_activiteiten}	radio (Matrix), Required<br>1 Oui<br>0 Non<br>2 Non applicable
106	andere_activiteiten<br>Show the field ONLY if:<br>[andere] = '1'		text
107	beschrijving_beweging	Quels termes décrivent le mieux les mouvements de votre enfant en ce moment? (Veuillez cocher toutes les réponses applicables)	checkbox<br>1 beschrijving_beweging___1 Non-coordonnés<br>2 beschrijving_beweging___2 Étranges<br>3 beschrijving_beweging___3 Désorganisés<br>4 beschrijving_beweging___4 Peu synchronisés<br>5 beschrijving_beweging___5 Maladroits<br>6 beschrijving_beweging___6 Instables<br>7 beschrijving_beweging___7 Lents<br>8 beschrijving_beweging___8 Précipités<br>9 beschrijving_beweging___9 Lourds<br>10 beschrijving_beweging___10 Patauds<br>12 beschrijving_beweging___12 Autre {andere2}<br><br>Custom alignment: LV

108	andere2<br>Show the field ONLY if: [beschrijving_beweging(12)] = '1'		text
109	langer	Pensez-vous que votre enfant nécessite plus de temps que d’autres enfants pour accomplir des tâches impliquant des mouvements?	radio<br>1 Oui<br>0 Non
110	vermoeid	Votre enfant se fatigue-t-il/elle en faisant des tâches impliquant des mouvements qu’il/elle trouve compliqués?	radio<br>1 Oui<br>0 Non
111	meer_vermoeid	En comparaison aux autres enfants, pensez-vous que le vôtre est plus fatigué que les autres en fin de journée?	radio<br>1 Oui<br>0 Non
112	uren_slaap	En moyenne, combien d’heures par nuit votre enfant dort-il/elle?	text
113	slaapkwaliteit	Votre enfant a-t-il/elle une qualité de sommeil réduite (réveils fréquents la nuit, difficultés à s’endormir, nombreux cauchemars, somnambulisme, etc.).	radio<br>1 Oui<br>0 Non
114	uitleg_slaapkwaliteit<br>Show the field ONLY if: [slaapkwaliteit] = '1'	Veuillez préciser.	notes, Required<br>Custom alignment: LV
115	zelf_keuze_thuis	Si votre enfant avait 30 minutes pour lui/elle seul(e) à la maison, que choisirait-il/elle de faire?	notes, Required<br>Custom alignment: LV
116	schermtijd	Combien de minutes de temps d'écran (télévision, ordinateur portable, téléphone mobile, console de jeux) votre enfant passe-t-il/elle en moyenne par jour à se détendre (sans compter les devoirs scolaires)?	text, Required
117	genieten_sport_fa	Votre enfant prend-t-il/elle plaisir à participer à des sports organisés et des activités physiques ?	radio<br>1 Oui<br>0 Non<br>2 Parfois
118	verduidelijking_genieten<br>Show the field ONLY if: [genieten_sport_fa] = '0' or [genieten_sport_fa] = '2'	Veuillez préciser.	notes<br>Custom alignment: LV
119	georg_vrije_tijd	Votre enfant participe-t-il/elle à des activités de loisirs organisées ? Il peut s'agir de sports, de jeux, de musique, de dessin, de mouvements de jeunesse, ...	radio, Required<br>1 Oui<br>0 Non
120	uitleg_vrije_tijd<br>Show the field ONLY if: [georg_vrije_tijd] = '1'	Le nom de chaque activité organisée à laquelle votre enfant participe actuellement Le temps passé à réaliser cette activité en minutes par semaine {sport1} {sport1_week} {sport2} {sport2_week} {sport3} {sport3_week} {sport4} {sport4_week}	descriptive
121	sport1		text
122	sport2		text
123	sport3		text
124	sport4		text
125	sport1_week		text
126	sport2_week		text
127	sport3_week		text
128	sport4_week		text
129	vrije_tijd_beschikbaar	Pensez-vous qu'il y a suffisamment d'activités de loisirs disponibles pour votre enfant dans votre quartier?	radio, Required<br>1 Oui<br>0 Non
130	mvpa	Combien de minutes par jour votre enfant pratique-t-il/elle une activité physique modérée à vigoureuse (la respiration s'accélère, le cœur se met à battre plus vite, mais l'enfant n'est pas essoufflé et peut encore parler normalement)?	radio, Required<br>1 Moins de 30 minutes<br>2 30 minutes à 1 heure<br>3 1 à 2 heures<br>4 Plus de 2 heures

131	bezorgd_fys_gez	Vous préoccupez-vous de l'impact des difficultés de mouvements de votre enfant sur sa santé physique?	radio, Required<br>1 Oui<br>0 Non
132	uitleg_bezorgd_fys_gez<br>Show the field ONLY if: [bezorgd_fys_gez] = '1'	Quelle est votre plus grande préoccupation?	notes<br>Custom alignment: LV
133	moeilijkheden_zindelijkheid	Votre enfant a-t-il eu des difficultés avec l'apprentissage de la propreté et/ou des toilettes ?	radio, Required<br>1 Oui<br>0 Non
134	uitleg_moeilijkheden_zindelijkheid<br>Show the field ONLY if: [moeilijkheden_zindelijkheid] = '1'	Veuillez préciser?	notes<br>Custom alignment: LV
135	uitleg_continentie	Les affirmations suivantes s'appliquent-elles à votre enfant ?	descriptive
136	stoelgangverlies	Dès l'âge de 4 ans, mon enfant n'a pas eu d'incontinence fécale.	radio (Matrix), Required<br>1 Vrai<br>0 Faux
137	urineverlies_overdag	Dès l'âge de 5 ans, mon enfant a toujours été complètement sec pendant la journée (pas de perte de gouttes non plus).	radio (Matrix), Required<br>1 Vrai<br>0 Faux
138	urineverlies_nacht	Dès l'âge de 5 ans, mon enfant a toujours été complètement sec la nuit (pas de perte de gouttes non plus).	radio (Matrix), Required<br>1 Vrai<br>0 Faux
139	articulatie	A-t-on déjà dit que votre enfant articule moins bien que les autres enfants?	radio, Required<br>1 Oui<br>0 Non
140	uitleg_articulatie<br>Show the field ONLY if: [articulatie] = '1'	Veuillez préciser?	notes<br>Custom alignment: LV
141	leerjaar	Section Header: *Domaine 3/5: Scolaires*<br>Dans quelle classe se trouve votre enfant?	text, Required
142	soort_onderwijs	Votre enfant fréquente-t-il l'enseignement ordinaire, l'enseignement spécialisé ou une école à pédagogie active?	radio, Required<br>1 Enseignement ordinaire<br>2 Enseignement spécialisé type {bijzonder_onderwijs}<br>3 Enseignement spécialisé, en intégration<br>4 Méthodes à pédagogie active (Montessori, Steiner, Freinet, Decroly)<br>5 Autre {andere_soort_onderwijs}
143	andere_soort_onderwijs<br>Show the field ONLY if: [soort_onderwijs] = '5'		text
144	bijzonder_onderwijs<br>Show the field ONLY if: [soort_onderwijs] = '2'		text
145	jaar_overdoen	Votre enfant a-t-il/elle déjà recommencé une année scolaire?	radio, Required<br>1 Oui<br>0 Non
146	waarom_jaar_overdoen<br>Show the field ONLY if: [jaar_overdoen] = '1'	Pourquoi a-t-il/elle recommencé une année scolaire?	notes<br>Custom alignment: LV
147	leerkracht_op_hoogte	Au début de l'année scolaire, son/ses enseignant(e)(s) était-il(s)/elle(s) au courant des difficultés de mouvements de votre enfant?	radio, Required<br>1 Oui<br>0 Non<br>2 Je ne sais pas

148	overleg_leerkracht	Le(s) avez-vous rencontré(e)(s) afin de discuter des besoins de votre enfant?	radio, Required<br>1 Oui, uniquement avec l'enseignant(e)(s)<br>2 Oui, avec l'enseignant(e)(s) et d'autres thérapeutes (consultation multidisciplinaire)<br>0 Non<br>3 Je ne sais pas
149	leerkracht_therapeut	L'enseignant(e) a-t-il/elle été en contact avec un thérapeute qui suit votre enfant pour pallier aux difficultés de votre enfant?	radio, Required<br>1 Oui<br>0 Non<br>2 Je ne sais pas
150	aanpassingen_klas	L'enseignant(e) a-t-il/elle procédé à des adaptations en classe?	radio, Required<br>1 Oui<br>0 Non<br>2 Je ne sais pas
151	ooit_ondersteuning_school	Votre enfant a-t-il déjà bénéficié d'un soutien de l'école?	radio, Required<br>1 Oui, par une équipe externe (réseaux de soutien)<br>2 Oui, par une équipe interne (coordinateur de soins, enseignant)<br>0 Non<br>3 Je ne sais pas
152	extra_tijd	Votre enfant reçoit-il/elle du temps supplémentaire pour compléter les tâches impactées par ses difficultés de mouvements (p.ex. tests/examens)?	radio, Required<br>1 Oui<br>0 Non<br>2 Je ne sais pas
153	meer_moe_school	Votre enfant est-il/elle plus fatigué(e) que les autres enfants à la fin d'un jour d'école?	radio, Required<br>1 Oui<br>0 Non
154	turnleerkracht_school	Y a-t-il un professeur d'éducation physique dans l'école de votre enfant?	radio, Required<br>1 Oui<br>0 Non
155	turnleerkracht_comm<br>Show the field ONLY if:<br>[turnleerkracht_school] = '1'	Le professeur d'éducation physique a-t-il/elle déjà communiqué avec vous ou l'enseignant(e) de votre enfant sur le soutien à lui apporter?	radio<br>1 Oui<br>0 Non<br>2 Je ne sais pas
156	ondersteuning_turnen<br>Show the field ONLY if:<br>[turnleerkracht_school] = '1'	Pensez-vous que votre enfant est soutenu(e) pour participer aux cours d'éducation physique à l'école?	radio<br>1 Oui<br>0 Non<br>2 Je ne sais pas
157	sportdag<br>Show the field ONLY if:<br>[turnleerkracht_school] = '1'	Votre enfant se sent-il/elle à l'aise lorsqu'il/elle participe à des journées sportives?	radio<br>1 Oui<br>0 Non<br>2 Je ne sais pas
158	uitdagingen_school	Quels sont les principaux défis pour votre enfant à l'école?	notes, Required<br>Custom alignment: LV
159	graag_school	Votre enfant aime-t-il/elle aller à l'école?	radio, Required<br>1 Oui<br>0 Non<br>2 Non applicable
160	niet_graag_school<br>Show the field ONLY if:<br>[graag_school] = '0' or [graag_school] = '2'	Veuillez spécifier?	notes<br>Custom alignment: LV
161	moeilijkheden_vrienden	Votre enfant a-t-il/elle des difficultés pour se faire des amis à l'école?	radio, Required<br>1 Oui<br>0 Non

162	neg_invloed_opleiding	Pensez-vous que les difficultés de mouvements de votre enfant impactent négativement ses capacités à mener à bien sa scolarité?	radio, Required<br>1 Oui<br>0 Non
163	opleiding_succesvol	Vous préoccupez-vous de la manière dont les difficultés de mouvements de votre enfant peuvent impacter sa capacité à compléter sa scolarité avec succès?	radio, Required<br>1 Oui<br>0 Non
164	grootste_bezorgdheid<br>Show the field ONLY if: [opleiding_succesvol] = '1'	Quelle est votre plus grande préoccupation?	notes<br>Custom alignment: LV
165	toekomstig_werk	Craignez-vous que les difficultés de mouvements de votre enfant affectent ses futures possibilités d'emploi?	radio, Required<br>1 Oui<br>0 Non
166	therapie_gevolgd	Section Header: *Domaine 4 /5 - Thérapie / intervention*<br>Votre enfant a-t-il/elle déjà suivi une thérapie pour le/la soutenir dans ses difficultés de mouvements?	radio, Required<br>1 Oui<br>0 Non
167	therapie_soort_2<br>Show the field ONLY if: [therapie_gevolgd] = '1'	Oui / Non Où? Combien de temps? (années) Ergothérapie {ergo_yn} {ergo_where} {ergo_duur} Kinésithérapie {kine_yn} {kine_where} {kine_duur} Psychomotricité {psychomot_yn} {psychomot_where} {psychomot_duur} Psychologie {psycho_yn} {psycho_where} {psycho_duur} Logopédie/orthophonie {logo_yn} {logo_where} {logo_duur} Neuropsychologie {neuropsycho_yn} {neuropsycho_where} {neuropsycho_duur} Autre: {andere_therapie} {andere_yn} {andere_where} {andere_duur}	descriptive
168	ergo_yn		radio<br>1 Oui<br>0 Non
169	kine_yn		radio<br>1 Oui<br>0 Non
170	psychomot_yn		radio<br>1 Oui<br>0 Non
171	psycho_yn		radio<br>1 Oui<br>0 Non
172	logo_yn		radio<br>1 Oui<br>0 Non
173	neuropsycho_yn		radio<br>1 Oui<br>0 Non
174	andere_yn		radio<br>1 Oui<br>0 Non
175	andere_therapie<br>Show the field ONLY if: [andere_yn] = '1'	Quelle autre thérapie a votre enfant reçu?	text
176	ergo_where<br>Show the field ONLY if: [ergo_yn] = '1'		dropdown<br>1 Pratique privée (pluridisciplinaire ou individuel)<br>2 Centre de réadaptation ambulatoire<br>3 Autre
177	kine_where<br>Show the field ONLY if: [kine_yn] = '1'		dropdown<br>1 Pratique privée (pluridisciplinaire ou individuel)<br>2 Centre de réadaptation ambulatoire<br>3 Autre

178	psychomot_where<br><br>Show the field ONLY if:<br>[psychomot_yn] = '1'		dropdown<br>1 Pratique privée (pluridisciplinaire ou individuel)<br>2 Centre de réadaptation ambulatoire<br>3 Autre
179	psycho_where<br><br>Show the field ONLY if:<br>[psycho_yn] = '1'		dropdown<br>1 Pratique privée (pluridisciplinaire ou individuel)<br>2 Centre de réadaptation ambulatoire<br>3 Autre
180	logo_where<br><br>Show the field ONLY if:<br>[logo_yn] = '1'		dropdown<br>1 Pratique privée (pluridisciplinaire ou individuel)<br>2 Centre de réadaptation ambulatoire<br>3 Autre
181	neuropsycho_where<br><br>Show the field ONLY if:<br>[neuropsycho_yn] = '1'		dropdown<br>1 Pratique privée (pluridisciplinaire ou individuel)<br>2 Centre de réadaptation ambulatoire<br>3 Autre
182	andere_where<br><br>Show the field ONLY if:<br>[andere_yn] = '1'		dropdown<br>1 Pratique privée (pluridisciplinaire ou individuel)<br>2 Centre de réadaptation ambulatoire<br>3 Autre
183	ergo_elders<br><br>Show the field ONLY if:<br>[ergo_where] = '3'	Où a votre enfant suivi l'ergothérapie?	text
184	kine_elders<br><br>Show the field ONLY if:<br>[kine_where] = '3'	Où a votre enfant suivi la kinésithérapie?	text
185	psychomot_elders_3<br><br>Show the field ONLY if:<br>[psychomot_where] = '3'	Où a votre enfant suivi la psychomotricité?	text
186	psycho_elders<br><br>Show the field ONLY if:<br>[psycho_where] = '3'	Où a votre enfant suivi la psychothérapie?	text
187	logo_elders<br><br>Show the field ONLY if:<br>[logo_where] = '3'	Où a votre enfant suivi la logopédie?	text
188	neuropsycho_elders<br><br>Show the field ONLY if:<br>[neuropsycho_where] = '3'	Où a votre enfant suivi la neuropsychologie?	text
189	anderetherapie_elders<br><br>Show the field ONLY if:<br>[andere_where] = '3'	Où a votre enfant suivi une autre sorte de thérapie?	text
190	ergo_duur<br><br>Show the field ONLY if:<br>[ergo_yn] = '1'		text
191	kine_duur<br><br>Show the field ONLY if:<br>[kine_yn] = '1'		text
192	psychomot_duur<br><br>Show the field ONLY if:<br>[psychomot_yn] = '1'		text
193	psycho_duur<br><br>Show the field ONLY if:<br>[psycho_yn]=1		text
194	logo_duur<br><br>Show the field ONLY if:<br>[logo_yn] = '1'		text

195	neuropsycho_duur<br>Show the field ONLY if: [neuropsycho_yn] = '1'		text
196	andere_duur<br>Show the field ONLY if: [andere_yn] = '1'		text
197	reden_geen_therapie<br>Show the field ONLY if: [therapie_gevolgd] = '0'	Y a-t-il des raisons pour lesquelles aucune thérapie n'a été commencée ?	radio, Required<br>1 Oui<br>0 Non
198	reden_geen_therapie_spec<br>Show the field ONLY if: [reden_geen_therapie] = '1'	Lesquelles?	notes<br>Custom alignment: LV
199	therapie_momenteel	Faites-vous actuellement appel à des services thérapeutiques pour aider votre enfant dans ses difficultés de mouvements ?	radio, Required<br>1 Oui<br>0 Non
200	reden_geen_therapie_2<br>Show the field ONLY if: [therapie_momenteel] = '0'	Y a-t-il des raisons spécifiques pour lesquelles votre enfant n'est pas actuellement en thérapie?	radio, Required<br>1 Oui<br>0 Non
201	reden_geen_therapie_spec_2<br>Show the field ONLY if: [reden_geen_therapie_2] = '1'	Lesquelles?	notes<br>Custom alignment: LV
202	therapie_soort_3<br>Show the field ONLY if: [therapie_momenteel] = '1'	Oui / Non Où? Nombre d'heures moyen par mois Dépenses mensuelles estimées Ergothérapie {ergo_yn_2} {ergo_where_2} {uurergo} {kostergo} Kinésithérapie {kine_yn_2} {kine_where_2} {uurkine} {kostkine} Psychomotricité {psychomot_yn_2} {psychomot_where_2} {uurpsychomot_2} {kostpsychomot_2} Psychologie {psycho_yn_2} {psycho_where_2} {uurpsycho} {kostpsycho} Logopédie/ orthophonie {logo_yn_2} {logo_where_2} {uurlogo} {kostlogo} Neuropsychologie {neuropsycho_yn_2} {neuropsycho_where_2} {uurneuropsycho} {kostneuropsycho} Autre: {andere_therapie_2} {andere_yn_2} {andere_where_2} {uurandere} {kostandere}	descriptive
203	andere_therapie_2<br>Show the field ONLY if: [andere_yn_2] = '1'	Quelle autre thérapie votre enfant a-t-il suivi?	text
204	ergo_yn_2		radio<br>1 Oui<br>0 Non
205	kine_yn_2		radio<br>1 Oui<br>0 Non
206	psychomot_yn_2		radio<br>1 Oui<br>0 Non
207	psycho_yn_2		radio<br>1 Oui<br>0 Non
208	logo_yn_2		radio<br>1 Oui<br>0 Non
209	neuropsycho_yn_2		radio<br>1 Oui<br>0 Non
210	andere_yn_2		radio<br>1 Oui<br>0 Non

211	ergo_where_2<br>Show the field ONLY if:<br>[ergo_yn_2] = '1'		dropdown<br>1 Pratique privée (pluridisciplinaire ou individuel)<br>2 Centre de réadaptation ambulatoire<br>3 Autre
212	kine_where_2<br>Show the field ONLY if:<br>[kine_yn_2] = '1'		dropdown<br>1 Pratique privée (pluridisciplinaire ou individuel)<br>2 Centre de réadaptation ambulatoire<br>3 Autre
213	psychomot_where_2<br>Show the field ONLY if:<br>[psychomot_yn_2] = '1'		dropdown<br>1 Pratique privée (pluridisciplinaire ou individuel)<br>2 Centre de réadaptation ambulatoire<br>3 Autre
214	psycho_where_2<br>Show the field ONLY if:<br>[psycho_yn_2] = '1'		dropdown<br>1 Pratique privée (pluridisciplinaire ou individuel)<br>2 Centre de réadaptation ambulatoire<br>3 Autre
215	logo_where_2<br>Show the field ONLY if:<br>[logo_yn_2] = '1'		dropdown<br>1 Pratique privée (pluridisciplinaire ou individuel)<br>2 Centre de réadaptation ambulatoire<br>3 Autre
216	neuropsycho_where_2<br>Show the field ONLY if:<br>[neuropsycho_yn_2] = '1'		dropdown<br>1 Pratique privée (pluridisciplinaire ou individuel)<br>2 Centre de réadaptation ambulatoire<br>3 Autre
217	andere_where_2<br>Show the field ONLY if:<br>[andere_yn_2] = '1'		dropdown<br>1 Pratique privée (pluridisciplinaire ou individuel)<br>2 Centre de réadaptation ambulatoire<br>3 Autre
218	ergo_elders_2<br>Show the field ONLY if:<br>[ergo_where_2] = '3'	Où suit votre enfant l'ergothérapie?	text
219	kine_elders_2<br>Show the field ONLY if:<br>[kine_where_2] = '3'	Où suit votre enfant la kinésithérapie?	text
220	psychomot_elders_2<br>Show the field ONLY if:<br>[psychomot_where_2] = '3'	Où suit votre enfant la psychomotricité?	text
221	psycho_elders_2<br>Show the field ONLY if:<br>[psycho_where_2] = '3'	Où suit votre enfant la psychothérapie?	text
222	logo_elders_2<br>Show the field ONLY if:<br>[logo_where_2] = '3'	Où suit votre enfant la logopédie/orthophonie?	text
223	neuropsycho_elders_2<br>Show the field ONLY if:<br>[neuropsycho_where_2] = '3'	Où suit votre enfant la neuropsychologie?	text
224	anderetherapie_elders_2<br>Show the field ONLY if:<br>[andere_where_2] = '3'	Où suit votre enfant une autre sorte de thérapie?	text
225	uurergo<br>Show the field ONLY if:<br>[ergo_yn_2] = '1'		text
226	uurkine<br>Show the field ONLY if:<br>[kine_yn_2] = '1'		text

227	uurpsychomot_2<br><br>Show the field ONLY if:<br>[psychomot_yn_2] = '1'		text
228	uurpsycho<br><br>Show the field ONLY if:<br>[psycho_yn_2] = '1'		text
229	uurlogo<br><br>Show the field ONLY if:<br>[logo_yn_2] = '1'		text
230	uurneuropsycho<br><br>Show the field ONLY if:<br>[neuropsycho_yn_2] = '1'		text
231	uurandere<br><br>Show the field ONLY if:<br>[andere_yn_2] = '1'		text
232	kostergo<br><br>Show the field ONLY if:<br>[ergo_yn_2] = '1'		text
233	kostkine<br><br>Show the field ONLY if:<br>[kine_yn_2] = '1'		text
234	kostpsychomot_2<br><br>Show the field ONLY if:<br>[psychomot_yn_2] = '1'		text
235	kostpsycho<br><br>Show the field ONLY if:<br>[psycho_yn_2] = '1'		text
236	kostlogo<br><br>Show the field ONLY if:<br>[logo_yn_2] = '1'		text
237	kostneuropsycho<br><br>Show the field ONLY if:<br>[neuropsycho_yn_2] = '1'		text
238	kostandere<br><br>Show the field ONLY if:<br>[andere_yn_2] = '1'		text
239	bijkomstige_kosten	Y a-t-il des frais supplémentaires que vous devez engager en raison des difficultés de mouvements de votre enfant (par exemple, un trampoline plus grand, des stylos spéciaux, des couverts adaptés, ...)?	radio, Required<br>1 Oui<br>0 Non
240	bijkomstige_kost_specifiek<br><br>Show the field ONLY if:<br>[bijkomstige_kosten] = '1'	Lesquelles?	notes<br>Custom alignment: LV
241	vrij_van_werk	Avez-vous besoin de vous absenter du travail pour permettre à votre enfant de suivre les sessions de thérapie?	radio, Required<br>1 Oui<br>0 Non
242	vrij_van_werk_uren<br><br>Show the field ONLY if:<br>[vrij_van_werk] = '1'	Combien d'heures par mois cela représenterait-il en moyenne?	text, Required
243	minder_gaan_werken	Un parent a-t-il diminué son temps de travail afin de mieux soutenir votre enfant ayant des difficultés de mouvements?	radio, Required<br>1 Oui<br>2 Non<br>3 Non, mais çela a été envisagé
244	lessen_missen	Arrive-t-il à votre enfant de manquer des cours pour suivre des séances de thérapie?	radio, Required<br>1 Oui<br>0 Non
245	lessen_missen_minuten<br><br>Show the field ONLY if:<br>[lessen_missen] = '1'	Combien de minutes par semaine manque-t-il/elle?	text, Required

246	voldoende_therapie	Avez-vous l'impression que votre enfant a bénéficié d'une thérapie suffisante pour l'aider dans ses difficultés de mouvements?	radio, Required<br>1 Oui<br>0 Non
247	voldoende_tips	Pensez-vous recevoir, vous en tant que parent, suffisamment de soutien et conseils pour aider votre enfant par rapport à ses problèmes de mouvements?	radio, Required<br>1 Oui<br>0 Non
248	finaniciele_ondersteuning	Recevez-vous une aide financière supplémentaire en raison du diagnostic du TDC?	radio, Required<br>0 Non<br>1 Oui, l'augmentation des allocations familiales<br>2 Oui, un budget d'assistance personnelle (BAP).<br>3 Oui, autre: {financiele_steun_andere}
249	financiele_steun_andere<br>Show the field ONLY if: [finaniciele_ondersteuning] = '3'		text
250	medicatie	Votre enfant reçoit-t-il/elle des médicaments en rapport avec ses problèmes de mouvements?	radio, Required<br>1 Oui<br>0 Non
251	medicatie_soort<br>Show the field ONLY if: [medicatie] = '1'	Veuillez préciser	text<br>Custom alignment: LV
252	bezorgd	Section Header: *Domaine 5/5 - Impact social et émotionnel sur l'enfant*<br>Êtes-vous préoccupé(e) par l'éventualité que les difficultés de mouvements de votre enfant aient un impact sur sa santé sociale et émotionnelle?	radio, Required<br>1 Oui<br>0 Non
253	bezorgdheden_voornaamste<br>Show the field ONLY if: [bezorgd]=1	Quelles sont vos principales préoccupations?	notes<br>Custom alignment: LV
254	vermijden_bew	Votre enfant se retire-t-il/elle ou évite de participer à des activités liées aux mouvements ?	radio (Matrix), Required<br>4 Toujours<br>3 Très souvent<br>2 Parfois<br>1 Rarement<br>0 Jamais
255	angstig_f	Votre enfant est-il/elle anxieux(se) d'apprendre ou de réaliser des activités liées aux mouvements ?	radio (Matrix), Required<br>4 Toujours<br>3 Très souvent<br>2 Parfois<br>1 Rarement<br>0 Jamais
256	angstig_bew	Votre enfant a-t-il/elle des difficultés à sociabiliser avec ses pairs?	radio (Matrix), Required<br>4 Toujours<br>3 Très souvent<br>2 Parfois<br>1 Rarement<br>0 Jamais
257	vrienden_m	Votre enfant a-t-il/elle des difficultés à se faire des ami(e)s?	radio (Matrix), Required<br>4 Toujours<br>3 Très souvent<br>2 Parfois<br>1 Rarement<br>0 Jamais

258	lichaamstaal	Votre enfant est-il/elle capable d'adapter son langage corporel à une situation sociale (contact visuel, orientation du corps) ?	radio (Matrix), Required<br>4 Toujours<br>3 Très souvent<br>2 Parfois<br>1 Rarement<br>0 Jamais
259	gesprek	Votre enfant parle-t-il/elle au bon moment dans une conversation?	radio (Matrix), Required<br>4 Toujours<br>3 Très souvent<br>2 Parfois<br>1 Rarement<br>0 Jamais
260	stil	Votre enfant reste-t-il/elle calme et à l'écoute lorsqu'une autre personne parle ?	radio (Matrix), Required<br>4 Toujours<br>3 Très souvent<br>2 Parfois<br>1 Rarement<br>0 Jamais
261	gelukkig	A quelle fréquence votre enfant se sent-il/elle heureux/se?	radio (Matrix), Required<br>4 Toujours<br>3 Très souvent<br>2 Parfois<br>1 Rarement<br>0 Jamais
262	verdietig	A quelle fréquence votre enfant se sent-il/elle triste?	radio (Matrix), Required<br>4 Toujours<br>3 Très souvent<br>2 Parfois<br>1 Rarement<br>0 Jamais
263	boos	A quelle fréquence votre enfant se sent-il/elle fâché(e) ou en colère?	radio (Matrix), Required<br>4 Toujours<br>3 Très souvent<br>2 Parfois<br>1 Rarement<br>0 Jamais
264	uitleg3	Choisissez pour chaque énoncé "Pas vrai", "Un peu vrai" ou "Très vrai". Répondez, s'il vous plaît, en vous basant sur le comportement de l'enfant au cours des six derniers mois.	descriptive
265	fysieke_klachten	Se plaint souvent de maux de tête ou de ventre ou de nausées	radio (Matrix), Required<br>0 Pas vrai<br>1 Un peu vrai<br>2 Très vrai
266	zorgen_kind	S'inquiète souvent, paraît souvent soucieux(se)	radio (Matrix), Required<br>0 Pas vrai<br>1 Un peu vrai<br>2 Très vrai
267	ongelukkig	Souvent malheureux(se), abattu(e) ou pleure souvent	radio (Matrix), Required<br>0 Pas vrai<br>1 Un peu vrai<br>2 Très vrai

268	nerveus	Anxieux(se) ou se cramponne aux adultes dans les situations nouvelles, perd facilement ses moyens	radio (Matrix), Required<br>0 Pas vrai<br>1 Un peu vrai<br>2 Très vrai
269	angstig	A de nombreuses peurs, facilement effrayé(e)	radio (Matrix), Required<br>0 Pas vrai<br>1 Un peu vrai<br>2 Très vrai
270	eenzaam	Plutôt solitaire, a tendance à jouer seul(e)	radio (Matrix), Required<br>0 Pas vrai<br>1 Un peu vrai<br>2 Très vrai
271	vriend	A au moins un(e) ami(e)	radio (Matrix), Required<br>0 Pas vrai<br>1 Un peu vrai<br>2 Très vrai
272	leuk_gevonden	Généralement aimé(e) des autres enfants	radio (Matrix), Required<br>0 Pas vrai<br>1 Un peu vrai<br>2 Très vrai
273	pesten	Harcelé(e) ou tyrannisé(e) par d'autres enfants	radio (Matrix), Required<br>0 Pas vrai<br>1 Un peu vrai<br>2 Très vrai
274	volwassen	S'entend mieux avec les adultes qu'avec les autres enfants	radio (Matrix), Required<br>0 Pas vrai<br>1 Un peu vrai<br>2 Très vrai
275	attent_gevoelens	Attentif(ve) aux autres, tient compte de ce qu'ils ressentent	radio (Matrix), Required<br>0 Pas vrai<br>1 Un peu vrai<br>2 Très vrai
276	delen	Partage facilement avec les autres enfants (friandises, jouets, crayons, etc.)	radio (Matrix), Required<br>0 Pas vrai<br>1 Un peu vrai<br>2 Très vrai
277	vriendelijk	Gentil(le) avec les enfants plus jeunes	radio (Matrix), Required<br>0 Pas vrai<br>1 Un peu vrai<br>2 Très vrai
278	helpen	Toujours prêt(e) à aider les autres (parents, professeurs, autres enfants)	radio (Matrix), Required<br>0 Pas vrai<br>1 Un peu vrai<br>2 Très vrai
279	behulpzaam	Aide volontiers quand quelqu'un s'est fait mal ou ne se sent pas bien	radio (Matrix), Required<br>0 Pas vrai<br>1 Un peu vrai<br>2 Très vrai
280	uitleg4	A quelle fréquence les difficultés de mouvements de votre enfant induisent les répercussions suivantes sur votre famille?	descriptive

281	bezorgd_gezin	Des soucis ou des préoccupations d'ordre émotionnel?	radio (Matrix), Required<br>4 Toujours<br>3 Très souvent<br>2 Parfois<br>1 Rarement<br>0 Jamais
282	tijd_jezelf	Réduction du temps alloué à vos besoins personnels ?	radio (Matrix), Required<br>4 Toujours<br>3 Très souvent<br>2 Parfois<br>1 Rarement<br>0 Jamais
283	soort_activiteiten	Réduction du type d'activités en famille?	radio (Matrix), Required<br>4 Toujours<br>3 Très souvent<br>2 Parfois<br>1 Rarement<br>0 Jamais
284	financiele_moeilijkh	Contraintes financières?	radio (Matrix), Required<br>4 Toujours<br>3 Très souvent<br>2 Parfois<br>1 Rarement<br>0 Jamais
285	bezorgd_toekomst	Préoccupations quant à son futur?	radio (Matrix), Required<br>4 Toujours<br>3 Très souvent<br>2 Parfois<br>1 Rarement<br>0 Jamais
286	invloed_gezinsleden	Les difficultés de mouvements de votre enfant affectent-elles la famille d'une autre manière ?	radio<br>1 Oui<br>0 Non
287	invloed_gezinsleden_uitleg<br>Show the field ONLY if: [invloed_gezinsleden] = '1'	Qui et de quelle manière?	notes<br>Custom alignment: LV
288	sterktes_kind	Quels sont les points forts de votre enfant ?	notes<br>Custom alignment: LV
289	impact_dcd_complete	Section Header: *Form Status*<br>Complete?	dropdown<br>0 Incomplete<br>1 Unverified<br>2 Complete

Instrument: **Demografische Informatie** (demografische_informatie) Enabled as survey Collapse

290	provinc	Dans quelle province habitez-vous?	radio, Required<br>1 Flandre orientale<br>2 Flandre occidentale<br>3 Brabant flamand<br>4 Anvers<br>5 Limbourg<br>6 Hainaut<br>7 Liège<br>8 Luxembourg<br>9 Namur<br>10 Brabant wallon<br><br>Custom alignment: LV
291	pc	Quel est votre code postal?	text, Required<br>Custom alignment: LV
292	income	Quel est le niveau de revenu de votre foyer ?	radio, Required<br>1 < 1962€/mois<br>2 1962 - 3924 €/mois<br>3 >3924 €/mois<br>4 Je préfère ne pas dire<br><br>Custom alignment: LV
293	mar_state	Quel est votre état civil ?	radio, Required<br>1 Célibataire<br>2 Marié(e)/Cohabitant(e) légal(e<br>3 Autre: {burgerlijke_staat_ander}<br><br>Custom alignment: LV
294	burgerlijke_staat_ander<br><br>Show the field ONLY if:<br>[mar_state] = '3'		text
295	diploma	Quel est le plus haut niveau d'études de la maman / parent 1?	radio, Required<br>1 Enseignement primaire<br>2 Enseignement secondaire<br>3 Enseignement supérieur, courte durée (bachelier)<br>4 Enseignement supérieur, longue durée (master, doctorat)<br><br>Custom alignment: LV
296	diploma_2	Quel est le plus haut niveau d'études du papa/ parent 2?	radio, Required<br>1 Enseignement primaire<br>2 Enseignement secondaire<br>3 Enseignement supérieur, courte durée (bachelier)<br>4 Enseignement supérieur, longue durée (master, doctorat)<br>5 Non applicable (famille monoparentale)<br><br>Custom alignment: LV
297	respondent	Qui remplit ce questionnaire?	radio, Required<br>1 Maman<br>2 Papa<br>3 Autre: {andere_resp}<br><br>Custom alignment: LV
298	andere_resp<br><br>Show the field ONLY if:<br>[respondent] = '3'		text<br>Custom alignment: LV

299	demografische_informatie_complete	Section Header: *Form Status*<br>Complete?	dropdown<br>0 Incomplete<br>1 Unverified<br>2 Complete
Instrument: **Slotvragen** (slotvragen) Enabled as survey			Collapse
300	meer_middelen_voor	S'il y avait plus de ressources de la part du gouvernement, où souhaiteriez-vous qu'elles aillent ? (par exemple, plus de soutien à l'école, plus de soutien financier, plus d'aide psychologique, ...)	notes<br>Custom alignment: LV
301	ondersteuning_prioriteit	Quel domaine de soutien présente la plus grande priorité pour vous en ce moment?	notes<br>Custom alignment: LV
302	noden_kind	Quel est le besoin actuel de votre enfant ?	notes<br>Custom alignment: LV
303	noden_ouder	Quel est votre besoin actuel à vous, en tant que parent ?	notes<br>Custom alignment: LV
304	opmerkingen	Y a-t-il d'autres commentaires ou préoccupations que vous désirez aborder?	notes<br>Custom alignment: LV
305	slotvragen_complete	Section Header: *Form Status*<br>Complete?	dropdown<br>0 Incomplete<br>1 Unverified<br>2 Complete
