# Supplementary material for: Understanding the impact of developmental coordination disorder on Belgian children and families: A national survey study
Source: PLoS One. 2025 Apr 22;20(4):e0320311. doi: 10.1371/journal.pone.0320311 (PMC12013903; doi:10.1371/journal.pone.0320311)
Supplement: S2. File — (PDF) [file pone.0320311.s002.pdf]

Diagnosis

	Absence of co-occurring conditions	Presence of co-occurring conditions
Mean age first concern	3.3±2.16y	3.4±2.29y
Mean age diagnosis	6.8±2.35y	6.9±2.37y

Functional impact

	Absence of co-occurring conditions	Presence of co-occurring conditions
Enjoyed participation in organized sport activities		
Yes	47.2	43.8
Sometimes	31.2	38.0
No	21.6%	18.2%
Engage in 60 minutes of daily moderate-to-vigorous physical activity, yes	82.9%	82.2%
Difficulties with toilet training, yes	88 (44.2%)	147 (50.3%)
Fecal incontinence after 4y, yes	40 (20.1%)	79 (26.7%)
Daytime urinary incontinence after 5y, yes	52 (26.1%)	85 (29.1%)
Bedwetting after 5y, yes	79 (39.7%)	117 (40.1%)

School and education

	Absence of co-occurring conditions	Presence of co-occurring conditions
Not feeling confident participating in occasional sport events at school	50 (25.1%)	88 (30.1%)
