# Supplementary material for: Understanding the impact of developmental coordination disorder on Belgian children and families: A national survey study
Source: PLoS One. 2025 Apr 22;20(4):e0320311. doi: 10.1371/journal.pone.0320311 (PMC12013903; doi:10.1371/journal.pone.0320311)
Supplement: S3 File — (PDF) [file pone.0320311.s003.pdf]

Inzicht in de impact van Developmental Coordination Disorder op Belgische kinderen en families: een nationaal onderzoek

Amy De Roubaix[1*], Griet Warlop[2], Dorine Van Dyck[3,4], Delphine Van Crombrugge[1], Silke Van den Abbeele[1], Melissa Licari[5], Hilde Van Waelvelde[1], Lynn Bar-On[1].

[1] Faculteit Geneeskunde en Gezondheidswetenschappen. Vakgroep Revalidatiewetenschappen, Universiteit Gent, België.

[2] Faculteit Geneeskunde en Gezondheidswetenschappen. Vakgroep Bewegings- en Sportwetenschappen, Universiteit Gent, België.

[3] Departement Neuropsychologie en Logopedie, Universitair Ziekenhuis Koningin Fabiola Kinderen (HUDERF) - Universitair Ziekenhuis van Brussel (H.U.B), Université libre de Bruxelles (ULB), Brussel, België.

[4] Departement Pediatrische Neurologie, Universitair Ziekenhuis Koningin Fabiola Kinderen (HUDERF) - Universitair Ziekenhuis Brussel (H.U.B), Université libre de Bruxelles (ULB), Brussel, België.

[5] Telethon Kids Institute, Universiteit van West-Australië, Perth, West-Australië, Australië

*Auteur bereikbaar voor vragen

E-mail: amy.deroubaix@ugent.be

Abstract

Achtergrond. Developmental Coordination Disorder (DCD) is een ondergewaardeerde en vaak gebagatelliseerde neurologische ontwikkelingsstoornis die vijf tot zes procent van de kinderen treft. Deze studie had als doel om de impact van DCD op kinderen en hun families in België in kaart te brengen.

Methode. De Australische 'Impact for DCD'-vragenlijst werd vertaald en aangepast aan de Belgische context. Ouders van 4 tot 18-jarige kinderen in België met bewegingsmoeilijkheden die overeenkomen met DCD werden uitgenodigd om de online vragenlijst in te vullen over diagnose, activiteiten, school, therapie en sociale en emotionele impact.

Resultaten. In totaal werden 491 kinderen geïncludeerd in de analyses. De eerste zorgen ontstonden voornamelijk thuis (61,4%) op de leeftijd van 3,3±2,25j, waarbij hulp werd gezocht op de leeftijd van 4,7±2,57j. De formele diagnose werd gesteld op de leeftijd van 6,9±2,36j, waarbij DCD (76,5%) en/of dyspraxie (38,4%) de meest voorkomende termen waren. DCD was over het algemeen onbekend, vooral op school. Bijna één op de vier kinderen (23,2%) dubbelde een jaar op school. Verminderde slaapkwaliteit (50,6%), vermoeidheid na schooltijd (76,0%), problemen met zindelijkheidstraining (47,9%) en spraakarticulatie (52,3%), evenals verhoogde emotionele problemen (52,7%) en problemen met leeftijdsgenoten (46,4%) kwamen veel voor. De meeste kinderen kregen therapie (89,2%), maar 59,1% van de ouders voelde zich niet voldoende ondersteund om hun kind te helpen. Bovendien nam 37,5% van de ouders regelmatig vrij van het werk om de therapie van hun kind te kunnen voorzien, terwijl 49,1% van de ouders hun werktijd had verminderd of dit overwoog (16,7%). Ouders uitten hun bezorgdheid over de toekomst en het welzijn van hun kind en pleitten met name voor begeleiding bij de ondersteuning van hun kind en meer bewustwording. Ouders maakten melding van belangrijke sterke punten van hun kinderen, waaronder empathie, creativiteit, cognitieve vaardigheden, doorzettingsvermogen en goede sociale en/of taalvaardigheden.

Conclusies. Deze resultaten benadrukken de significante impact van DCD vanuit het perspectief van ouders. De volgende stap is het onderzoeken van manieren om ouders te empoweren en te ondersteunen, het bewustzijn te vergroten en samen te werken met beleidsmakers om deze uitdagingen aan te pakken.

Verklaringen

Open source gepubliceerd met de steun van de Universitaire Stichting van België, het GE37 kinderrevalidatiefonds en het ENLIGHT Wetenschappelijk Onderzoeksnetwerk (RISE/SRN/ENtITY/1). De auteurs hebben geen concurrerende belangen te melden die relevant zijn voor de inhoud van dit artikel.

Inleiding

Developmental Coordination Disorder (DCD) is een neurologische ontwikkelingsstoornis (NOS) die 5-6% van de schoolgaande kinderen treft [1]. Personen met DCD ervaren significant meer problemen met het verwerven en uitvoeren van gecoördineerde motorische vaardigheden in vergelijking met hun leeftijdsgenoten en leermogelijkheden, wat een significante invloed heeft op verschillende aspecten van hun leven, zoals de productiviteit op school, activiteiten in het dagelijks leven en professionele activiteiten. De moeilijkheden zijn al op jonge leeftijd aanwezig en kunnen niet worden verklaard door een andere medische aandoening (bijv. cerebrale parese, spierdystrofie, verstandelijke beperking). De uitdagingen van DCD gaan vaak verder dan motorische vaardigheden en beïnvloeden verschillende domeinen van het functioneren. De moeilijkheden roepen gevoelens van falen op, wat resulteert in frustraties op korte termijn, een verminderd gevoel van eigenwaarde en risico's op depressie en angst op lange termijn [2]. Als gevolg hiervan vermijden mensen vaak fysieke activiteit en participeren ze minder, wat mogelijk leidt tot verminderde sociale vaardigheden, minder vriendschappen en meer gevoelens van isolatie [3]. DCD heeft niet alleen gevolgen voor het individu, maar ook voor het hele gezin: gezinnen ondernemen minder sociale activiteiten en voelen zich beperkt in hun dagelijks leven [4]. Ouders kunnen een verminderd welzijn ervaren, zich meer zorgen maken en moeten veel tijd en energie besteden om aan de behoeften van hun kind te voldoen, wat resulteert in aanzienlijke opvoedingsstress [5]. Belangrijk is ook dat ouders vaak hun professionele carrière moeten aanpassen, minder uren moeten gaan werken of helemaal moeten stoppen met werken, wat leidt tot financiële spanningen als gevolg van een lager inkomen en beperkte toegang tot financiële ondersteuning [4, 6].

Heterogeniteit in DCD is niet alleen duidelijk in de ernst en categorieën van beïnvloede domeinen, maar ook in het samen voorkomen met andere aandoeningen. Tot 70% van de kinderen met DCD heeft een of meer bijkomende aandoeningen, waaronder Autisme Spectrum Stoornis (ASS), ADHD (Attention-Deficit/Hyperactivity Disorder), specifieke leerstoornissen (bijv. dyslexie, dyscalculie) en spraakontwikkelingsdyspraxie [6-8]. Naast het feit dat er verschillende ondersteuningssystemen nodig zijn, is het cruciaal om te erkennen dat kinderen met bijkomende aandoeningen vaak een minder goede uitkomst hebben, waaronder een lagere kwaliteit van leven [9] en emotionele gezondheidsproblemen [10]. Hoewel DCD naar schatting minstens één kind per klas treft, is het een van de minst bekende aandoeningen bij kinderen en wordt het te vaak niet herkend [6]. In Duitsland en het Verenigd Koninkrijk was slechts 59% van de clinici in verschillende specialismen bekend met DCD [11]. In Canada toonde 100% van de kinderergotherapeuten kennis over DCD, in tegenstelling tot 41% van de kinderartsen en 22% van de huisartsen [12]. Het gebrek aan bekendheid bij clinici draagt bij aan de onderkenning van de aandoening, vertragingen in de diagnose en het minimaliseren van de zorgen van ouders, waardoor ouders zich geïsoleerd en niet gesteund voelen [13-15]. Er is ook een gebrek aan bekendheid van DCD in de onderwijssector, waar slechts 23% van de Canadese leerkrachten [16] en 35% van de Australische leerkrachten [17] bekend bleken te zijn met DCD. Het gebrek aan bewustzijn over DCD op scholen kan leiden tot een verkeerde interpretatie en bagatellisering van de waargenomen moeilijkheden, aangezien ten minste enkele van deze kinderen ten onrechte werden beschouwd als 'lui' of als kinderen die 'niet hard genoeg hun best doen' [18, 19]. Wat de algemene bekendheid betreft, rapporteerde een Canadees onderzoek dat slechts 6% van de ouders ooit van DCD had gehoord [16]. Ouders kunnen moeite hebben om de oorzaak van de moeilijkheden van hun kind te begrijpen en realiseren zich misschien niet dat er een onderliggende diagnostische aandoening zou kunnen zijn. Daarom kunnen ze gefrustreerd raken als hun kind

problemen heeft met het uitvoeren van basistaken [20]. Deze frustratie kan de gespannen gezinsdynamiek verergeren en bijdragen aan de emotionele problemen van het kind.

De "Impact for DCD"-beweging werd opgericht in een poging om de brede impact van DCD te begrijpen en te pleiten voor systemische verandering. Het team van Licari et al. (2021), dat in eerste instantie in Australië werd opgericht, ontwikkelde een uitgebreide enquête om de impact van DCD op kinderen en hun families in kaart te brengen en om gebieden te identificeren die het belangrijkst zijn voor families en die verandering vereisen [6]. Sindsdien hebben veel landen zich bij deze beweging aangesloten om de wereldwijde impact uitgebreid in kaart te brengen. Het is van cruciaal belang om in elk land studies uit te voeren, omdat de impact aanzienlijk kan verschillen afhankelijk van de gezondheidszorg en het onderwijssysteem, de toegang tot middelen, sociale ondersteuningsnetwerken, de cultuur en het algemene bewustzijn van de aandoening. Tot nu toe zijn er onderzoeksresultaten over de impact van DCD gepubliceerd in Australië [6, 21, 22], Canada [23-25] en de Verenigde Staten [26]. Deze studie wil bijdragen aan deze beweging door de impact van DCD in België in kaart te brengen, een land waar eigenlijk nog maar weinig onderzoek verricht is naar de impact van DCD met uitzondering van één kwalitatieve studie over vroege DCD-kenmerken [27]. Het identificeren van uitdagingen en verbeterpunten binnen de Belgische context kan helpen bij het bepalen van aandachtsgebieden en het prioriteren van ondersteuning voor gezinnen. De resultaten van deze studie kunnen beleidsbeslissingen met betrekking tot onderwijs, gezondheidszorg en sociale ondersteuning ondersteunen.

Methoden

Deze studie werd goedgekeurd door de Medisch Ethische Commissie van het Universitair Ziekenhuis Gent (België) (ONZ-2022-0203) en geregistreerd bij ClinicalTrials.Gov (NCT05499143). Na het lezen van het geïnformeerde toestemmingsformulier gaven alle deelnemers digitaal toestemming door de toestemmingsvraag bevestigend te beantwoorden voordat ze deelnamen.

Enquête en deelnemers

Volgens de richtlijnen voor het proces van crossculturele aanpassing van zelfrapportagemaatregelen [28] werd de Australische Impact for DCD-enquête [6] vertaald naar het Nederlands en Frans en cultureel aangepast aan de Belgische context. Dit proces omvatte vier tolken die de originele Engelse enquête in het Nederlands (n=2) en Frans (n=2) vertaalden. Vervolgens werd tijdens een vergadering een synthese gemaakt van de Nederlandse en Franse vertalingen, waarna vier verschillende tolken deze syntheses terug naar het Engels vertaalden. Er werd een vergadering gehouden om eventuele verschillen in interpretatie te bespreken die een aanpassing aan de Nederlandse en Franse versie vereisten. In elk stadium werd schriftelijk gerapporteerd. Vervolgens beoordeelde een uitgebreide commissie van deskundigen (waaronder acht vertalers, een ouder van een kind met DCD, een vertegenwoordiger van de Belgische non-profitorganisatie voor ouderondersteuning bij DCD "Dyspraxis vzw", vier professionals uit de gezondheidszorg en acht onderzoekers op het gebied van DCD) de Belgische enquête en besprak de enquête-items. Net als in de Australische enquête werden er vragen opgenomen over diagnose, therapie, activiteiten en participatie, onderwijs, sociale en emotionele gevolgen en de gevolgen voor het gezin. Er werden drie subschalen van de Strengths and Difficulties Questionnaire (SDQ) [29] gebruikt: emotionele uitdagingen, uitdagingen van leeftijdsgenoten en prosociaal gedrag. Bij gebrek aan Belgische normen voor kinderen van 4 tot 10 jaar werden Britse SDQ-normen gebruikt, terwijl

Belgische normen werden gebruikt voor kinderen van elf tot achttien jaar. De oorspronkelijke enquête werd aangevuld met verschillende nieuwe thema's, waaronder zindelijkheidstraining, spraakarticulatie en de sterke punten van de kinderen. De enquête bevatte zowel meerkeuzevragen als open vragen en het invullen duurde gemiddeld 45 minuten (S1 File).

De enquête werd gelanceerd op 17 augustus 2022 en bleef beschikbaar tot 17 december 2022. Het verzamelen van gegevens werd ondersteund door REDCap elektronische data capture tools, gehost aan de Universiteit van Gent [30]. Ouders van 4 tot 18-jarige kinderen woonachtig in België met bewegingsmoeilijkheden die overeenkomen met DCD (d.w.z. die niet verklaard konden worden door een andere medische aandoening) werden uitgenodigd om de online enquête in te vullen. De enquête richtte zich op de ervaringen van ouders, dus alle antwoorden werden door ouders gegeven. Het onderzoek werd gepromoot via sociale media, officiële communicatie van de universiteit en de non-profit organisatie voor ouderondersteuning Dyspraxis vzw. Uitnodigingen om deel te nemen werden via brieven en e-mails verstuurd naar een groot aantal Belgische zorgverleners in de pediatrie (bv. kinesitherapeuten, ergotherapeuten, kinderartsen, revalidatiecentra, logopedisten, psychomotorische therapeuten) en scholen voor buitengewoon onderwijs, zodat een landelijke dekking verzekerd was. Deelnemers werden uitgesloten als ze niet in België woonden, niet voldeden aan het leeftijdscriterium (4-18 jaar), geen bewegingsmoeilijkheden hadden die overeenkwamen met DCD, een andere medische aandoening hadden die de bewegingsmoeilijkheden verklaarde, of de vragenlijst niet volledig invulden.

Analyse

Er werden zowel kwantitatieve als kwalitatieve analyses uitgevoerd. Het verkennen van de reacties op open vragen leverde kwalitatieve inzichten op die de enquêtegegevens aanvulden en een rijker begrip van het onderwerp boden. Voor elk item werd het totale aantal deelnemers gerapporteerd die een bepaald antwoord hadden geselecteerd, samen met het percentage ten opzichte van de volledige steekproef of de relevante deelsteekproef als het vervolgvragen betrof. Kwantitatieve analyse uitgevoerd in JASP [31] omvatte een vergelijking tussen antwoorden van kinderen met en zonder bijkomende aandoeningen, die binair werden gecodeerd als aanwezig of afwezig. Vanwege de niet-normale verdeling van de numerieke gegevens werden Mann-Whitney U-tests gebruikt, waarbij effectgroottes werden gerapporteerd als Rank-Biserial r. Het significantieniveau werd vastgesteld op $p<0,05$. Voor categorische gegevens werden chi-kwadraattests uitgevoerd. Kwalitatieve inductieve thematische analyse werd uitgevoerd om de onderliggende concepten van open vragen te onderscheiden. Twee onderzoekers codeerden onafhankelijk van elkaar alle antwoorden met NVivo 14-software [32]. De codes werden vervolgens via een iteratief proces in thema's geordend. Eventuele discrepanties werden opgelost door middel van discussie. Om de betrouwbaarheid van de gegevensverzameling en -analyse te vergroten, werden de inclusie- en exclusiecriteria grondig nagevraagd in het eerste deel van de vragenlijst. Onderzoekerstriangulatie werd geïmplementeerd door alle open vragen dubbel te coderen en discussies aan te gaan binnen het multidisciplinaire onderzoeksteam om de resultaten te valideren. Bovendien werd een verhoogde reflexiviteit bevorderd om een grondig en onbevooroordeeld onderzoek te garanderen.

Resultaten

Deelnemers

Ouders van 1256 kinderen stemden in eerste instantie in met deelname. Uiteindelijk werden 477 families van 491 kinderen uitgesloten voor analyse, vanwege het uitsluiten van deelnemers waarvan het kind niet in België woonde (n=97), niet tussen de 4 en 18 jaar oud was (n=19), geen bewegingsmoeilijkheden vertoonde die overeenkwamen met DCD (n=22), een andere medische aandoening had die de bewegingsmoeilijkheden kon verklaren (n=329), of omdat de enquête niet werd voltooid (n=298). Kenmerken van de steekproef worden beschreven in **Tabel 1**. De studiesteekproef (n=491) heeft een gemiddelde leeftijd van 10,4 ± 3,37 jaar en bestaat voornamelijk uit te vroeg geboren mannelijke kinderen, met een leeftijd tussen de 7 en 12 jaar. De meerderheid zijn oudste kinderen, afkomstig uit gezinnen met een gemiddeld tot hoog inkomen, en woont voornamelijk in Vlaanderen. Minstens één bijkomende diagnose kwam voor bij 59,5% van de kinderen, waarbij ADHD de meest voorkomende bijkomende aandoening was (31,0%), gevolgd door ASS (22,8%) en specifieke leerstoornissen (21,8%).

Tabel 1. Kenmerken van de steekproef (n=491).

	N	%
**Geslacht**		
Mannelijk	373	76.0
Vrouwelijk	118	24.0
**Zwangerschapsduur**		
<28 weken	3	0.6
28 - 32 weken	11	2.2
32 - 37 weken	63	12.8
>37 weken	412	83.9
Ontbrekend	2	0.4
**Leeftijd**		
4-6 jaar	64	13.0
7-9 jaar	145	29.5
10-12 jaar	156	31.8
13-15 jaar	80	16.3
16-18 jaar	46	9.4
**Positie in de kinderrij**		
Oudste	199	40.5
Middelste	55	11.2
Jongste	169	34.4
Enig kind	68	13.8
**Maandelijks netto gezinsinkomen**		
Laag (< 1962 euro)	36	7.3
Gemiddeld (1962 – 3924 euro)	190	38.7
Hoog (> 3924 euro)	205	41.8
Zeg ik liever niet	60	12.2
**Woonplaats**		
Vlaanderen	377	76.8

Wallonië	99	20.2
Brussel Hoofdstedelijk Gewest	15	3.1
**Bijkomende aandoeningen**		
ADHD	152	31.0
Autisme Spectrum Stoornis	112	22.8
Specifieke leerstoornis	107	21.8
Spraak-taal ontwikkelingsstoornis	65	13.2
Dysgrafie	38	7.7
Angstoornis of depressive	24	4.9
Epilepsie	10	2.0
Gehoorproblemen	4	0.8
Ticstoornis	3	0.6
Centraal Visuele Inperking	3	0.6
Andere	19	3.9

Afkortingen: ADHD, Attention Deficit Hyperactivity Disorder; n, aantal.

Diagnose

Diagnostisch traject

Bezorgdheid over de bewegingen van het kind werd voor het eerst geuit door de ouders toen de kinderen gemiddeld 3,3±2,25j oud waren. Deze zorgen werden voornamelijk thuis gesignaleerd (n=243, 61,4%), gevolgd door meldingen van de kleuterschool (2,5 tot 5j) (n=156, 39,4%), basisscholen (6 tot 12j) (n=39, 9,8%), of kinderdagverblijven/onthaalmoeders (<2,5j) (n=38, 9,6%) (**Fig 1**).

Figuur 1. Percentage omgevingsfactoren waarover men zich voor het eerst zorgen maakte (n=491): de eerste zorgen ontstonden vooral thuis.

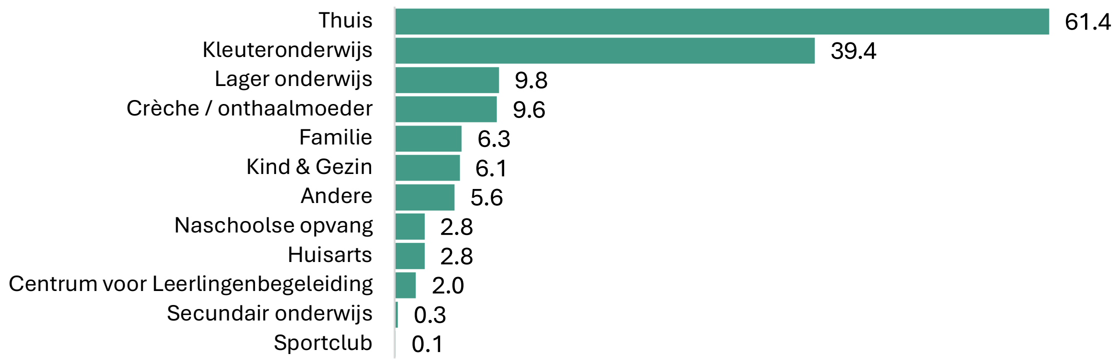

Respondenten konden meerdere antwoorden selecteren.

Ouders zochten hulp toen hun kinderen gemiddeld 4,7±2,57j oud waren. In totaal had 80,7% (n=396) van de kinderen een formele diagnose gekregen op de gemiddelde leeftijd van 6,9±2,36y. Er was geen significant verschil tussen kinderen met of zonder bijkomende aandoeningen in leeftijd van eerste zorg (U = 28254,0, p = 0,74, r = -0,017; S2 File) of diagnose (U = 16641,0, p=0,428, r = -0,048; S2 Bestand). De meest voorkomende diagnosetermen waren DCD (n=294, 76,5%) en/of dyspraxie (n=152, 38,4%) (Tabel 2). Deze werden meestal gediagnosticeerd door neurologen (n=178, 44,9%),

Centra voor Ontwikkelingsstoornissen (n=120, 30,3%), kinesitherapeuten (n=112, 28,3%) of psychiaters (n=52, 13,1%) (**Fig. 2**).

Figuur 2. Professionals (%) die formeel de diagnose bewegingsmoeilijkheden hebben gesteld bij het kind (n=396): Verschillende professionals en instellingen geven de diagnose Developmental Coordination Disorder.

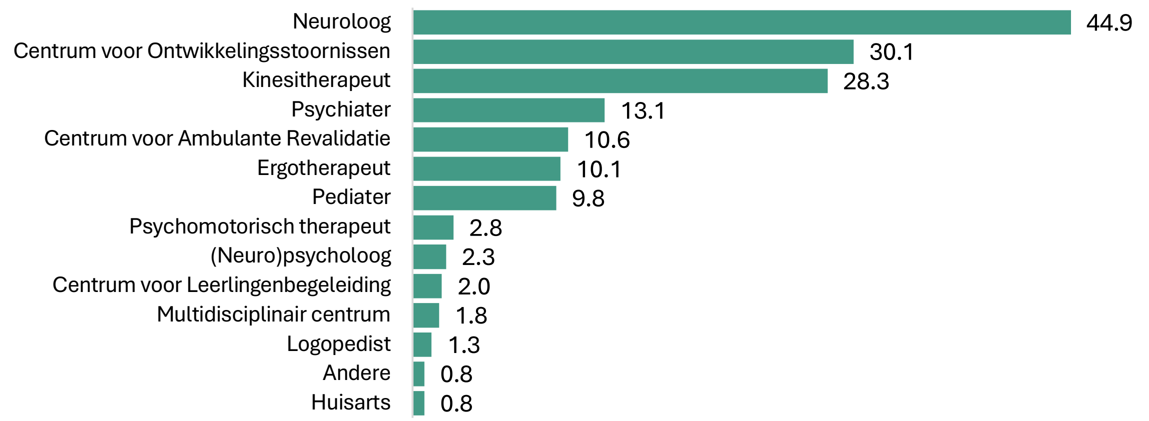

Respondenten konden meerdere antwoorden selecteren.

Figuur 3. Assessments uitgevoerd binnen het diagnostisch proces van bewegingsmoeilijkheden (%) (n=396): Motorische beoordelingen en ouderlijke anamnese kwamen vaak voor, terwijl consultaties van leerkrachten minder vaak voorkwamen.

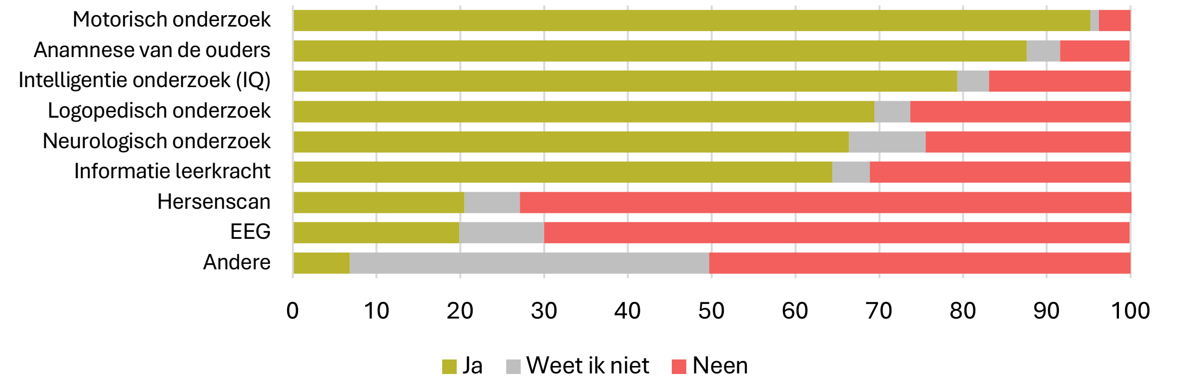

Respondenten konden meerdere antwoorden selecteren.
<u>Afkortingen:</u> IQ, intelligentiequotiënt; EEG, elektro-encefalogram.

Als een kinesitherapeut de bewegingsmoeilijkheden diagnosticeerde, gebeurde dit meestal in samenwerking met een arts (98 van de 112 gevallen, 77,5%). Tijdens het diagnostisch proces werden motorische testen (95,2%) en ouderlijke anamnese (87,6%) vaak uitgevoerd naast tal van andere

beoordelingen (**Fig 3**). Volgens de ouders werden leerkrachten in 64,4% van de gevallen geraadpleegd. Bij 95 kinderen (19,3%) werd tot op heden geen formele diagnose van bewegingsmoeilijkheden gesteld, maar werden hun bewegingsmoeilijkheden vaak omschreven als bijvoorbeeld 'een risico op / kenmerken / vermoeden van DCD', 'motorische coördinatieproblemen' of 'niet-vloeiende motoriek' (**Tabel 2**).

Tabel 2. Diagnoses en beschrijvingen van bewegingsmoeilijkheden.

	N	%
**Formele diagnose van bewegingsmoeilijkheden (n=396)**		
Developmental Coordination Disorder (DCD)*	294	74.2
Dyspraxie	152	38.4
Hypotonie	32	8.1
Hypermobiliteit	29	7.3
Trouble d'Acquisition de la Coordination (TAC)	8	2.0
Sensorische Integratie Stoornis	1	0.3
Minimal Brain Damage	1	0.3
Andere	1	0.3
**Beschrijving van bewegingsproblemen bij afwezigheid van een formele diagnose (n=95)**		
Risico's / kenmerken / vermoeden van DCD	57	60.0
Problemen met motorische coördinatie	46	48.4
Houterige motoriek	46	48.4
Onhandig	45	47.4
Vertraagde motorische ontwikkeling	30	31.6
Motorische planningsmoeilijkheden	25	26.3
Vertraagde motorische mijlpalen	16	16.8
Werd niet beschreven door een professional	16	16.8
Reflexintegratiestoornis	9	9.5
Andere	1	1.1

Respondenten konden meerdere antwoorden selecteren.
*De DCD-categorie omvatte de Engelse term (Developmental Coordination Disorder), de letterlijke vertaling in het Nederlands (coördinatie ontwikkelingstoornis) en de letterlijke vertaling in het Frans (trouble développemental de la coordination; TDC).
Afkortingen: DCD, Developmental Coordination Disorder; n, aantal.

Bewustzijn en impact van de diagnose DCD

De meeste ouders (n=232, 58,6%) hadden vóór de diagnose van hun kind nog nooit van DCD gehoord, maar waren het erover eens dat het krijgen van een diagnose goed was voor henzelf (n=362, 91,4%) en voor hun kind (n=339). Op de vraag naar de waarde van de diagnose gaven ouders aan dat een diagnose voor hun kinderen leidde tot beter zelfbegrip, meer begrip van zowel ouders als leerkrachten, meer ondersteuning op school en toegang tot therapie (n=339). In gevallen waarin de diagnose als niet helpend werd ervaren, merkten ouders echter een grotere kloof tussen hun kind en anderen en een negatieve invloed op het zelfvertrouwen van het kind (n=57). Voor de ouders (n=362) bracht de bevestiging van de diagnose erkenning en bevestiging van hun zorgen. Het hielp hen de situatie beter te begrijpen, vergemakkelijkte de acceptatie en stelde hen gerust dat de moeilijkheden van hun kind reëel waren en dat men niet overdreven had. Dit bracht verlichting na een periode van onzekerheid, stress, twijfel en frustratie.

De bevestiging van een diagnose stelde ouders in staat om de problemen van hun kind beter te verwoorden, wat leidde tot meer begrip en minder negatieve opmerkingen van anderen. Bovendien konden ze meer steun, begrip en geduld bieden. In de gevallen waarin de diagnose ouders niet hielp (n=34), kwam dit vooral door een gebrek aan begeleiding na de diagnose en de aanhoudende onzekerheid, omdat ze "alles zelf moesten uitzoeken". Hoewel de meeste artsen en kinesitherapeuten die door de familie werden bezocht, bekend waren met de term DCD, kende ongeveer één op de vier huisartsen DCD niet (**Fig 4**). Bovendien had minder dan de helft van de klasleerkrachten en een derde van de turnleerkrachten voorkennis van DCD. Tot slot had slechts 11 tot 13% van de vrienden en familie van de aandoening gehoord.

Figuur 4. Percentage specialisten, schoolinstellingen en vrienden/familie met wie de familie contact opnam die op de hoogte waren van de diagnose Developmental Coordination Disorder: de meeste professionals in de gezondheidszorg waren op de hoogte van DCD, maar de kennis was beperkt in onderwijs- en vrijetijdsinstellingen en bij familie en vrienden.

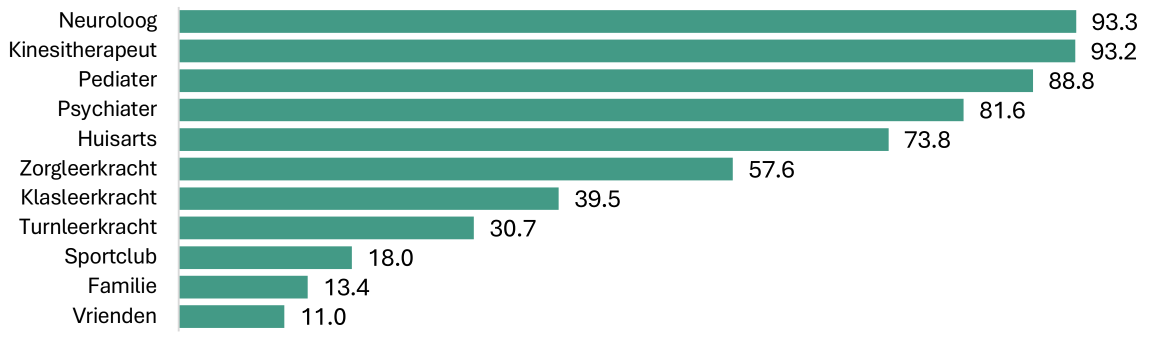

Functionele impact

Vrijetijds- en sportactiviteiten

Ouders meldden dat de meeste kinderen (n=372, 75,8%) deelnamen aan georganiseerde vrijetijdsactiviteiten. Minder dan de helft van de kinderen (n=222, 45,2%) vond deelname aan georganiseerde sportactiviteiten leuk, terwijl 35,2% (n=173) er soms plezier aan beleefde en 19,6% (n=96) er nooit plezier in had. Deze verhoudingen verschilden niet afhankelijk van de aanwezigheid van bijkomende aandoeningen ($\chi^2 = 0,3$, p = 0,596 en $\chi^2 = 3,0$, p = 0,224; S2 Bestand). Van de kinderen nam 82,5% (n=405) niet deel aan 60 minuten dagelijkse matige tot intense fysieke activiteit, zonder verschil tussen kinderen met of zonder bijkomende aandoeningen ($\chi^2 = 2,6$, p = 0,454; S2 Bestand). Een derde van de ouders (n=170, 34,6%) uitte echter bezorgdheid over de mogelijke nadelige effecten van verminderde fysieke activiteit op de gezondheid van hun kind en 55,6% (n=273) meldde een gebrek aan beschikbare vrijetijdsactiviteiten die waren afgestemd op de behoeften van hun kind.

Activiteiten van het dagelijks leven

Ouders meldden dat hun kinderen uitdagingen ondervonden bij verschillende activiteiten van het dagelijks leven, fijne en grove motoriek en mobiliteit (bvb. Aspecten zoals fietsen en gebruik van het openbaar vervoer) (**Fig 5**). Ongeveer de helft van de ouders gaf aan problemen te ondervinden met zindelijkheidstraining (n=235, 47,9%), waaronder fecale incontinentie (bruine strepen of encopresis) na 4j (n=118, 24,0%), urine-incontinentie overdag na 5j (n=137, 27,9%), en bedplassen (enuresis) na 5j (n=196, 39,9%). Geen van deze uitkomsten was significant verschillend in aanwezigheid van

bijkomende aandoeningen (χ^2 0,0-3,0, p=0,08-0,88, S2 Bestand). Kwalitatieve analyse (n=234) gaf aan dat vertraagde en/of langdurige zindelijkheidstraining een veelvoorkomende zorg was onder deze ouders. Ouders meldden dat problemen met zindelijkheidstraining vaak terugkwamen als reactie op stress of veranderingen in de omgeving, zoals vakanties of de overgang naar een nieuw schooljaar. Moeilijkheden met de motorische vaardigheden (bijv. uitkleden, richten, afvegen), gedragsproblemen (bijv. concentratie, uitstelgedrag, weigering om naar het toilet te gaan) en sensorische problemen (bijv. slecht lichaamsbesef, moeite met ontspannen, voorkeur voor natte doekjes) werden door de ouders vaak genoemd als factoren die bijdroegen aan deze problemen. Een moeder van een zesjarig kind beschreef de moeilijkheden van haar kind met zindelijkheidstraining als volgt: "Hij wist niet hoe hij het moest ophouden, hij plaste naast het toilet (doet hij nog steeds). Hij wist niet hoe hij moest mikken en hij weet nog steeds niet hoe hij zijn billen goed moet afvegen."

Figuur 5. Ouders gaven aan dat hun kinderen moeite hadden met verschillende activiteiten (%) (n=491).

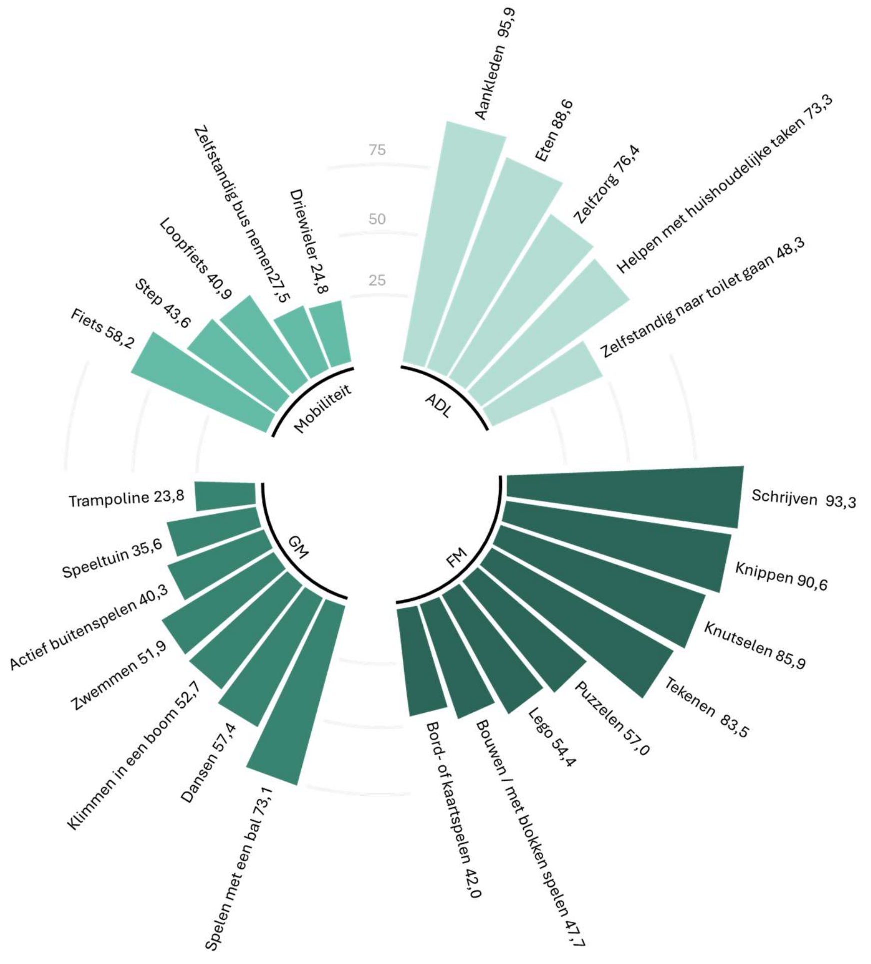

<u>Afkortingen:</u> ADL, activiteiten van het dagelijks leven; FM, fijne motorische activiteiten, GM, grove motorische activiteiten.

De helft van de kinderen rapporteerde een verminderde slaapkwaliteit (n=248, 50,6%), wat in sterkere mate werd waargenomen bij kinderen met bijkomende aandoeningen (40,0% vs. 57,9%, χ^2 = 15,2, p<0,001). Kwalitatieve analyses gaven aan dat ouders (n=248) vooral problemen noemden bij het in slaap vallen van hun kinderen, waarbij ze dit vaak toeschreven aan zorgen: "[Hij is] een echte denker die moeite heeft om de slaap te vatten. Zijn hoofd zit vaak vol en hij vindt geen rust". Slaapstoornissen werden vaak geassocieerd met parasomnie, zoals nachtmerries, slaapwandelen en slaappraten, met af en toe meldingen van slaapgerelateerde angst.

Spraak articulatie

Ongeveer de helft van de ouders (n=257, 52,3%) rapporteerde zorgen over de spraakarticulatie van hun kinderen, met meer zorgen bij kinderen met bijkomende aandoeningen (44,5% versus 57,7%; χ^2 = 8,3, p = 0,004). Ouders (n=250; 50,9%) rapporteerden problemen met betrekking tot onduidelijke articulatie, waaronder fonetische en fonologische fouten, zoals problemen met de uitspraak van specifieke klanken, het omdraaien van lettergrepen en woordvervorming of verkeerde uitspraak. Daarnaast werden vaak suprasegmentale problemen genoemd, zoals te snel spreken, vloeiendheidsproblemen zoals stotteren en veranderingen in stemvolume. Deze problemen werden soms in verband gebracht met gevoelens van onzekerheid: "Ze heeft de neiging om onduidelijk, stil en mompelend te spreken. Dit is meestal omdat ze zich onzeker voelt. Ze kan echter wel duidelijk articuleren, bijvoorbeeld als ze aan het lezen is." Bovendien werd er melding gemaakt van mondmotorische uitdagingen, waaronder hypotonie en problemen met tongpositionering, samen met kwijlen en de neiging om de mond open te houden.

School en onderwijs

Schooltraject en plezier

De meeste kinderen volgden regulier onderwijs, waarbij 78,8% (n=387) naar reguliere scholen ging en 3,7% (n=18) naar alternatieve methodescholen (bijv. Steiner, Montessori, Freinet). Bijzonder onderwijs werd gevolgd door 13,0% (n=64) en een minderheid volgde inclusief regulier onderwijs (n=15, 3,1%). Slechts zeven kinderen (1,4%) volgden andere vormen van onderwijs (thuisonderwijs, privéonderwijs, geen onderwijs of onderwijs op maat voor hoogbegaafde kinderen). Bijna één op de vier kinderen moest minstens één jaar dubbelen (n=114, 23,2%). Dit aandeel was significant hoger bij kinderen met bijkomende aandoeningen (16,5% versus 27,8%; χ^2 = 8,5, p = 0,003). Kwalitatieve analyse (n=114) gaf aan dat kinderen vaak het laatste jaar van de kleuterschool (meestal rond 5 jaar) dubbelden, voornamelijk omdat ze “te jong” werden geacht of niet over de nodige motorische vaardigheden beschikten voor een succesvolle overgang naar de basisschool, terwijl bij oudere kinderen algemene achterstanden in schoolse vaardigheden (lezen, taal, rekenen) redenen waren om een jaar over te doen. Ouders hadden gemengde gevoelens over het dubbelen van een schooljaar: "Hij had een leerkracht die heel weinig begrip had [voor zijn problemen] en die hem ook vertelde dat hij dom was en niets kon. Zijn zelfvertrouwen was helemaal weg, dus [brachten we hem naar een] nieuwe school [...] om hem wat gemoedsrust te geven".

Volgens de ouders vond één op de vijf kinderen (n=93, 18,9%) het helemaal niet leuk om naar school te gaan. Kwalitatieve analyse (n=188) gaf aan dat kinderen meer stress ervaren om goed te presteren op school, terwijl ze ervaren dat ze trager zijn en 'anders dan anderen': "Hij geeft zijn maximum om het minimum te bereiken". Verder meldden ouders dat hun kinderen werden gepest, dat ze moeite hadden om aansluiting te vinden bij leeftijdsgenoten, dat ze geen begrip kregen, en dat ze meer

vermoeid waren. Ouders uitten hun bezorgdheid over de uitputting van hun kind aan het einde van de schooldag, waarbij ze opmerkten dat het langer duurt om taken af te ronden, dat leren moeilijker wordt en dat er weinig tijd is voor ontspanning. De meerderheid van de ouders (n=373, 76,0%) rapporteerde een grotere vermoeidheid bij hun kind in vergelijking met leeftijdsgenootjes aan het einde van de schooldag en dit percentage was zelfs nog hoger bij kinderen met bijkomende aandoeningen (70,0% versus 79,4%; $\chi^2 = 4{,}6$, p = 0,03).

Communicatie en ondersteuning op school

De meerderheid van de leerkrachten werd aan het begin van het schooljaar geïnformeerd over de bewegingsproblemen van het kind (n=410, 83.5%) en overleg met de ouders werd ofwel alleen met de klasleerkracht (n=129, 26.3%) of met een multidisciplinair team (n=261, 53.2%) gehouden om de behoeften van het kind in de klas te bespreken. Aan het begin van het schooljaar was er bij ongeveer de helft van de kinderen (n=237, 48,3%) contact tussen de kinesitherapeut en de klasleerkracht. Volgens de ouders werden voor 59,9% (n=294) van de kinderen aanpassingen doorgevoerd door de leerkrachten, vaak inclusief extra tijd voor beoordelingen of examens (n=248, 50,5%). Extra ondersteuning werd geboden door de zorgleerkracht (n=147, 29,9%) en/of een externe ondersteuner (n=229, 46,6%). Met betrekking tot turnlessen waren 39,4% (n=190) van de ouders zich bewust dat hun kind enige vorm van ondersteuning kreeg. Verder voelde 28,0% (n=138) van de kinderen zich niet zeker om deel te nemen aan occasionele sportevenementen op school (bvb. sportdag), waarbij geen significant verschil werd waargenomen op basis van de aanwezigheid van bijkomende aandoeningen ($\chi^2 = 3{,}9$, p = 0,149; S2 Bestand).

Therapie en interventie

De meeste kinderen kregen therapie (n=438, 89,2%), waarbij kinesitherapie, logopedie en psychologie het vaakst werden gemeld (**Tabel 3**). Ongeveer één op de drie kinderen (n=143, 29,1%) miste af en toe lessen vanwege therapie. Hoewel meer dan de helft van de ouders vond dat hun kind voldoende therapie kreeg om de bewegingsproblemen aan te pakken (n=281, 57,2%), gaven zij ook aan dat zij als ouders niet voldoende ondersteund werden om hun kind te helpen met deze uitdagingen (n=290, 59,1%). Eén op de tien kinderen (n=45, 9,2%) gebruikte medicatie, voornamelijk om de focus en aandacht te verbeteren in verband met een bijkomende ADHD-diagnose.

Tabel 3. Overzicht van therapiegeschiedenis, sector van therapiebezoek en huidig therapiebezoek per maand.

**Ooit therapie gevolgd (%)**				**Sector therapiebezoek (%)**				**Huidige therapie per maand (uren)**		
	Ja	Nee	Ont-brekend	Privé	CAR	Andere	Ont-brekend	Gemiddelde ± SD	Bereik	N*
**Kinesitherapie**	78.0	8.1	13.8	69.2	15.1	1.8	13.8	3.6 ± 2.1	[0.5 - 12]	142/196
**Logopedie**	59.9	22.4	17.7	62.9	19.4	4.1	13.6	3.8 ± 2.0	[0.5 - 10]	88/133
**Psychologie**	34.0	43.0	23.0	58.1	22.8	4.8	14.4	1.9 ± 1.2	[0.16 - 5]	39/62
**Ergotherapie**	29.9	46.2	23.8	38.1	40.8	8.8	12.2	4.3 ± 3.6	[0.5 -25)	53/83
**Neuropsychologie**	15.1	59.9	25.1	58.1	16.2	10.8	14.9	2.0 ±1.3	[0.5 - 4.0]	15/28
**Psychomotorische therapie**	10.2	81.9	7.9	76.0	4.0	2.0	18.0	2.8 ± 1.2	[1.0 – 4.0]	8/11
**Andere**	14.3	29.9	55.8	65.7	7.1	18.6	8.6	3.1 ± 3.4	[0.25 - 12]	19/24

*Omdat de antwoorden op deze specifieke vragen onvolledig waren, werd het aantal respondenten uitgedrukt in verhouding tot het totale aantal respondenten dat momenteel die therapie volgt.
Afkortingen: CAR, Centrum Ambulante Revalidatie.

Sociaal-emotionele impact

Op de SDQ vertoonde ongeveer de helft van de kinderen verhoogde niveaus van door de ouders gerapporteerde emotionele uitdagingen (n=259, 52,7%) en problemen met leeftijdsgenoten (n=228, 46,4%), terwijl de meerderheid pro-sociaal gedrag vertoonde binnen het typische bereik (n=365, 74,3%) (**Fig. 6**). Daarnaast meldde de helft van de ouders dat hun kind moeite had om vrienden te maken (n=221, 45,0%). Al deze variabelen kwamen significant vaker voor bij kinderen met een bijkomende aandoening, met meer emotionele uitdagingen (57,8% versus 68,5%, U = 24197,0, p = 0,001, r = -0,168), meer problemen met leeftijdsgenoten (48.7% versus 66,8%; U = 22311,0, p < 0,001, r= -0,233), meer problemen met het maken van vrienden (33,0% versus 53,3%; χ^2 = 19,7, p < 0,001), en lagere niveaus van pro-sociaal gedrag (22,1% versus 28,1%; U = 33293,5, p = 0,006, r = 0,144).

Figuur 6. Verdeling van prestaties (%) op drie subtests van de Strengths and Difficulties Questionnaire (n=491): Ongeveer de helft van de kinderen ervaart een klinische range van emotionele problemen en problemen met leeftijdsgenoten, hoewel de meerderheid adequaat pro-sociaal gedrag vertoont.

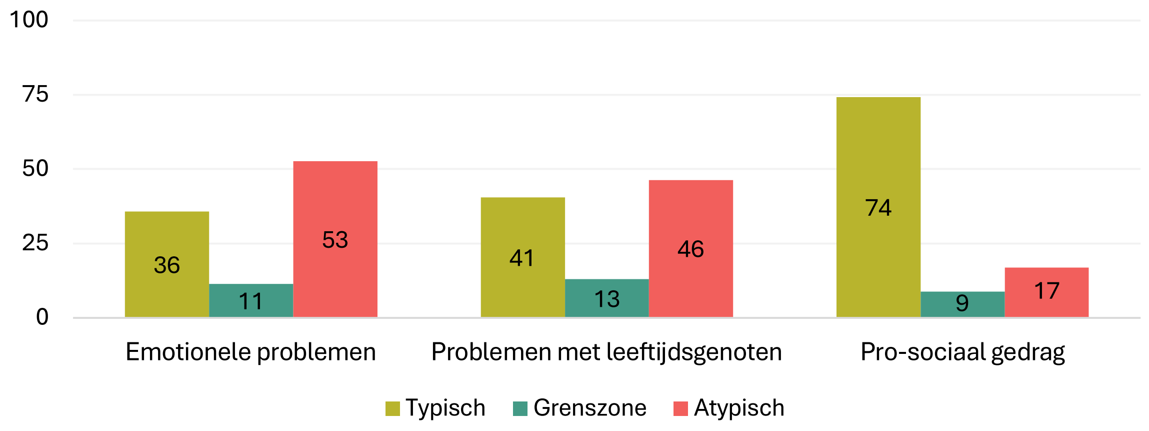

Impact op het gezin

Meer dan de helft van de ouders uitte zorgen over de toekomst van hun kind, ervoer emotionele zorgen, vond beperkte tijd voor hun eigen persoonlijke behoeften en meldde dat het soort gezinsactiviteiten beperkt was (**Fig 7**). Daarnaast gaven ze aan zich zorgen te maken over de negatieve invloed van motorische problemen op het studiesucces van hun kind (n=329, 67%) en toekomstige professionele activiteiten (n=318, 64,8%). Ouders hadden ook te maken met financiële lasten (**Fig 7**). Maandelijkse uitgaven voor therapie werden gerapporteerd bij 154 (31,4%) kinderen. Gemiddeld bedroegen deze uitgaven €144,21 per maand (tussen €0-771). De meerderheid van de ouders ontving geen extra financiële steun voor het volgen van therapie buiten de verplichte ziektekostenverzekering (n=358, 72,9%), hoewel 22,8% (n=112) een verhoogde kinderbijslag ontving. Veel ouders (n=292, 59,5%) meldden extra uitgaven in verband met de bewegingsmoeilijkheden van hun kind, zoals aangepast schoolmateriaal, kleding en privélessen om specifieke motorische vaardigheden aan te leren. Bovendien nam 37,5% (n=184) regelmatig vrij van het werk om de therapie van hun kind te kunnen verzorgen, terwijl 49,1% (n=241) hun werkuren hadden verminderd of dit overwogen (16,7%, n=82).

Figuur 7. De veelzijdige impact van bewegingsmoeilijkheden op gezinnen: ouders maken zich de meeste zorgen over de toekomst en het emotionele welzijn van hun kind en rapporteren beperkte tijd voor hun eigen persoonlijke behoeften (n=491) (%).

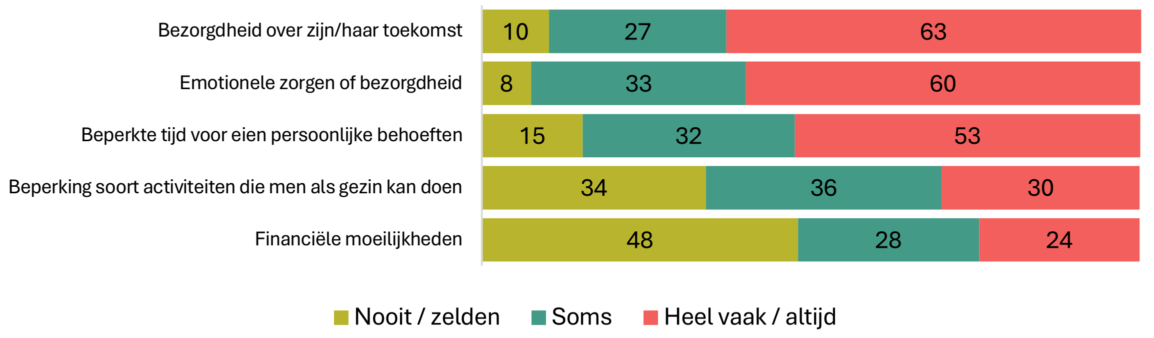

Kwalitatieve analyse (n=471) toonde aan dat de topprioriteit van ouders is om iets te doen aan het beperkte bewustzijn en begrip van DCD, wat er vaak toe leidt dat anderen hun zorgen als overdreven ervaren. Ze hebben behoefte aan duidelijkere begeleiding bij de ondersteuning van hun kind met DCD en pleiten voor minder administratieve rompslomp en hulp bij het navigeren door het complexe ondersteuningssysteem. Bovendien voelen ze een gebrek aan ondersteuning, waardoor velen van hen uitgeput raken en moeite hebben om genoeg tijd te vinden om hun kind effectief te helpen.

Sterke punten van kinderen met DCD

De identificatie van gerapporteerde sterke punten werd uitgevoerd door middel van kwalitatieve analyse (**Fig 8**). Ouders (n=476) rapporteerden dat hun kinderen zeer empathisch en zorgzaam waren en een sterk rechtvaardigheidsgevoel hadden, terwijl creativiteit werd gezien in probleemoplossende vaardigheden, artistieke expressie en een goed gevoel voor humor. Verder benadrukten ze de cognitieve vaardigheden van hun kinderen, waaronder intelligentie, een sterk geheugen en leergierigheid. Doorzettingsvermogen, veerkracht en optimisme werden ook beschreven. Eén ouder lichtte deze eigenschap toe door te zeggen: "Ze doet haar uiterste best om vooruit te komen. Ze doet er alles aan om te doen wat er van haar gevraagd wordt en als ze het [de] eerste keer niet goed doet, probeert ze het opnieuw tot ze slaagt." Een subgroep van ouders benadrukte de taalvaardigheid van hun kinderen, waaronder sterke verbale vaardigheden, meertaligheid en leesvaardigheid. Tot slot werden goede sociale vaardigheden genoemd. Een moeder vatte samen: "Onze zoon is ongelooflijk creatief, out of the box denkend maar vooral heel sociaal, grappig, ruimdenkend, heeft een heel groot hart, is heel empathisch en kan perfect partijen verzoenen en een moeilijke situatie ontwapenen."

Discussie

Dit onderzoek, dat betrekking heeft op de ervaringen van 491 Belgische kinderen met hun ouders, benadrukt de wijdverspreide impact op zowel het kind als het gezin. Gemiddeld kregen kinderen twee jaar na hun eerste zoektocht naar hulp een diagnose. Eén op de vijf kinderen kreeg echter (nog) geen formele diagnose voor hun bewegingsmoeilijkheden. Ondanks inspanningen zoals

Figuur 8. Door ouders gerapporteerde sterke punten bij kinderen met Developmental Coordination Disorder (n=476).

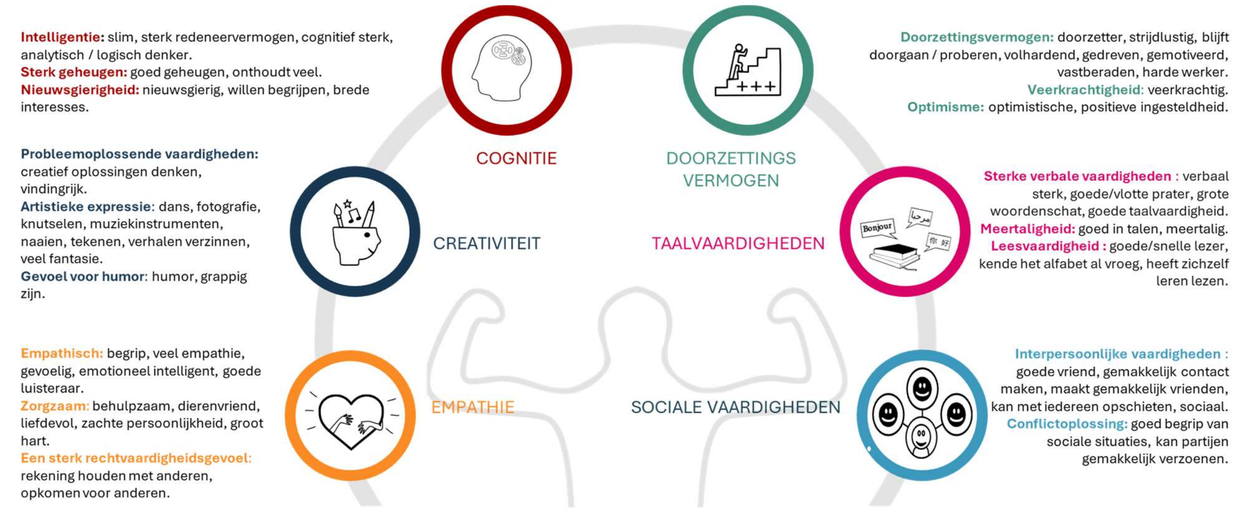

multidisciplinair overleg op school en toegang tot therapie, geven ouders aan dat er een gebrek is aan adequate ondersteuning, aangepaste vrijetijdsactiviteiten en algemene bewustwording, wat invloed heeft op het welzijn van het kind en henzelf. Het herkennen en aanpakken van deze uitdagingen en tegelijkertijd de sterke kanten van deze kinderen erkennen, is essentieel om een betere toekomst te garanderen. Het is belangrijk om te erkennen dat in dit onderzoek een controlegroep ontbreekt, waardoor we deze ervaringen niet kunnen vergelijken met die van kinderen met een typische ontwikkeling.

Diagnostisch traject en ondersteuning

Veel hulpverleners in België gebruiken de correcte diagnostische term "Developmental Coordination Disorder", hoewel beschrijvende termen zoals "dyspraxie" nog steeds in gebruik zijn. De internationale richtlijnen voor de diagnose van DCD zijn goed bekend in België, wat mogelijk de hogere naleving van de juiste diagnostische terminologie verklaart en de iets oudere leeftijd bij diagnose (6,9j) in vergelijking met Australische (5,3j) en Amerikaanse resultaten (4,9j) [6, 26]. Internationale richtlijnen [33] raden specifiek aan om voorzichtig te zijn met het diagnosticeren van kinderen voor de leeftijd van vijf jaar, wat waarschijnlijk ook van invloed is op de timing van diagnoses. Interessant genoeg had de aanwezigheid van bijkomende aandoeningen geen significante invloed op de leeftijd van bezorgdheid of diagnose. Dergelijke aandoeningen kunnen echter de aandacht afleiden van motorische problemen, waardoor de herkenning mogelijk vertraagd wordt, terwijl omgekeerd multidisciplinaire onderzoeken voor kinderen met meervoudige diagnoses de identificatie van motorische problemen kunnen versnellen. Desalniettemin is de kloof van twee jaar tussen het zoeken naar hulp en de bevestiging van de diagnose aanzienlijk, met onduidelijke toegang tot ondersteuning gedurende deze periode. Het merendeel van de gezinnen gaf aan veel baat te hebben bij het krijgen van een diagnose, wat de noodzaak van een juiste en tijdige diagnose benadrukt. Verder onderzoek is nodig om de impact van een vroege diagnose op de stress bij ouders en de prognose voor het kind te onderzoeken. In Australië en Canada [6, 24] speelden ouders ook een centrale rol in het vroegtijdig identificeren van zorgen, maar meer dan de helft had nog nooit van de aandoening gehoord, waardoor de eerste zoektocht naar hulp waarschijnlijk werd uitgesteld. Een

grotere bekendheid van DCD onder ouders kan helpen bij een vroegtijdige herkenning. Tot slot voelden ouders zich onvoldoende ondersteund om hun kind na de diagnose te helpen. Onze bevindingen onderstrepen daarom dat er meer inspanningen moeten worden geleverd om ouders pre- en postdiagnostische ondersteuning te bieden.

Interessant genoeg werden er vaker zorgen geuit in Belgische kleuterscholen (39,4%) dan in Canada (8,0%). Dit verschil kan mogelijk worden verklaard door het langere kleuteronderwijs in België (drieënhalf jaar) in vergelijking met Canada, waar kleuteronderwijs slechts één jaar duurt [24]. In tegenstelling tot internationale richtlijnen [33] werden leerkrachten niet vaak betrokken bij het diagnostisch proces. Het integreren van leerkrachten in zowel het diagnostische als het ondersteuningsproces zou een tijdige diagnose kunnen vergemakkelijken en het bewustzijn en de ondersteuning voor kinderen met DCD op scholen kunnen vergroten. Dit is nodig omdat veel kinderen in ons onderzoek minstens één schooljaar moesten dubbelen, een praktijk waarvan de effectiviteit ter discussie staat. De impact van het dubbelen op de academische vooruitgang zou beperkt kunnen zijn [34], en hoewel er op korte termijn verbeteringen in het emotioneel welzijn worden waargenomen, wijzen de resultaten op lange termijn op mogelijke negatieve effecten op de sociale acceptatie [35]. Tingle, Schoeneberger en Algozzine [36] pleiten voor diversifiëren in plaats van dubbelen, wat bij minder dan de helft van de kinderen in dit onderzoek gebeurde. Toch werd er bij ongeveer de helft van de kinderen wel overlegd met ouders en/of therapeuten, en werden er aanpassingen in de klas gedaan, wat duidt op inspanningen om in te spelen op verschillende leerbehoeften. Er was echter minder communicatie tussen ouders en gymleraren. Ondanks hun training in motorische ontwikkeling waren de meeste turnleerkrachten in dit onderzoek niet bekend met DCD. Uit eerdere literatuur blijkt echter dat turnleerkrachten de helft van de kinderen correct herkenden wanneer hen werd gevraagd om kinderen te identificeren die waarschijnlijk slecht zouden presteren op een motorische test. Dit onderstreept hun potentieel als een nog onbenutte bron voor het opsporen van kenmerken van DCD op school [37].

Wijdverspreide uitdagingen

In dit onderzoek onderzochten we uitdagingen bij kinderen met DCD en bevestigden we verhoogde sociale en emotionele problemen [38, 39], slaapstoornissen [40-42], verminderde fysieke activiteit [43], vermoeidheid [44], problemen met zindelijkheidstraining [27, 45], spraakarticulatieproblemen [46, 47] en een aanzienlijke impact op het gezin [4-6].

De hoge percentages van sociale en emotionele uitdagingen onderstrepen het belang van het aanpakken van deze aspecten, aangezien mensen met DCD een verhoogd risico lopen op het ontwikkelen van depressie en angst [48]. De resultaten benadrukken bovendien de noodzaak van meer bewustzijn over DCD binnen de geestelijke gezondheidszorg [49], omdat de mogelijke aanwezigheid van onderliggende DCD vaak over het hoofd wordt gezien wanneer mensen hulp zoeken [50]. Hoewel dit een veelbelovend interventiedoel lijkt, zijn er tot nu toe geen onderzoeken gedaan naar de effectiviteit van psychotherapie voor mensen met DCD. De oorzaak en de aard van de gerapporteerde lagere slaapkwaliteit in deze studie vereist verder onderzoek, aangezien de gerapporteerde prevalentie van 50,6% in deze studie hoger is dan wat wordt gezien bij kinderen met een typische ontwikkeling (3-36%) [51, 52]. Hoewel slaapproblemen bij mensen met ASS en ADHD in verband zijn gebracht met factoren zoals melatonine regulatie disfunctie, obstructieve slaapapneu en circadiane ritme slaapstoornis [53], zou het ook gerelateerd kunnen zijn aan een lager emotioneel welbevinden wat leidt tot meer zorgen vóór het slapen [54]. Verbazingwekkend genoeg voldeed 82,5% van de kinderen in onze onderzoeksgroep niet aan de aanbevolen 60 minuten

dagelijkse matige tot hoge fysieke activiteit, wat meer dan vier keer zo hoog is als de 19,6% die werd gerapporteerd onder kinderen van zes tot negen jaar in de Europese WHO-regio [55]. Deze bevindingen ondersteunen internationale richtlijnen die pleiten voor meer nadruk op fysieke activiteit voor mensen met DCD. Ondanks wereldwijde trends die wijzen op een afname van fysieke activiteit bij kinderen [55], kunnen neurodivergente kinderen extra ondersteuning nodig hebben om te genieten van beweging. De verminderde fysieke activiteit kan leiden tot verhoogde vermoeidheid bij deze kinderen, terwijl vermoeidheid ook kan leiden tot verminderde fysieke activiteit. Aangezien bijna elk aspect van het dagelijks leven motorische coördinatie vereist, wat vooral voor kinderen met DCD een uitdaging is, is het niet verwonderlijk dat de meeste ouders een verhoogde vermoeidheid bij hun kinderen rapporteren. Tot op heden zijn problemen met zindelijkheidstraining bekend bij ASS [56] en ADHD [57], maar ze blijven grotendeels onopgemerkt bij DCD. De prevalentie van urine-incontinentie overdag (27,9%) en bedplassen (39,9%) na 5 jaar in deze studie is aanzienlijk hoger dan de prevalentie van respectievelijk 10% [58] en 21% [59]. Naar het toilet gaan vereist niet alleen een reeks gecoördineerde motorische vaardigheden, zoals het openen en sluiten van ritssluitingen, mikken op het toilet en de billen schoonvegen, maar ook andere vaardigheden, zoals goede planning en organisatie (bijvoorbeeld toiletpauzes plannen tijdens de pauze, zorgen voor voldoende toiletpapier te nemen als dat buiten de toiletruimte hangt), ruimtelijke oriëntatie (het toilet kunnen vinden) en het omgaan met zintuiglijke prikkels (aandrang voelen, omgaan met bepaalde sensaties). Het opsporen van deze zindelijkheidsproblemen is cruciaal, omdat ze vaak samengaan met psychosociale problemen en problemen met leeftijdsgenoten [60] en geassocieerd worden met een verminderde respons op de behandeling [61]. Ondanks dat slechts bij 13,2% van de kinderen sprake was van een officiële diagnose van een taal-spraakontwikkelingsstoornis, werden problemen met spraakarticulatie (52,3%) gemeld bij bijna vier keer zoveel kinderen. Interessant is dat diagnoses van spraaktaalproblemen minder vaak voorkwamen in de Belgische steekproef in vergelijking met andere landen, waar dit tussen de 23% en 40% lag [6, 24, 26]. Articulatie is slechts één aspect van het complexe proces van spraakproductie, dat veel coördinatie vereist van de spieren die de uitademing en het verfijnen van de geluiden die we maken (zoals de tong, mond en kaak) controleren. Spraakproductie kan beïnvloed worden door zelfvertrouwen, maar ook door mondmotorische coördinatieproblemen die vaak worden vastgesteld bij kinderen met DCD [46, 47]. Hoewel de ernst van de problemen met spraakarticulatie in dit onderzoek niet is beoordeeld, is het essentieel te erkennen dat deze problemen een aanzienlijke en onafhankelijke invloed kunnen hebben op het emotionele welzijn en de algehele kwaliteit van leven. De bovengenoemde uitdagingen benadrukken het belang van het implementeren van holistische en gepersonaliseerde benaderingen.

De bevindingen van het onderzoek bevestigen verder dat de impact verder kan reiken dan het individu en invloed kan hebben op de dagelijkse activiteiten en het welzijn van de ouders, waardoor ouders aandringen op meer bewustzijn over DCD en begeleiding om hun kind effectief te ondersteunen. De meeste interventiestudies hebben zich echter voornamelijk gericht op het 'behandelen' van het kind met DCD. Tot op heden zijn er nog geen formele DCD-ouderondersteuningsprogramma's (zoals psycho-educatie) onderzocht, hoewel dergelijke programma's nuttig zijn gebleken bij andere ontwikkelingsstoornissen [62, 63]. Slechts één onderzoek vergeleek Cognitive Orientation to Daily Occupational Performance (CO-OP) training bij kinderen met DCD, met en zonder ouderbegeleiding, maar toonde geen statistische verschillen tussen de groepen wat betreft verbetering in de ADL en motorische prestaties van kinderen [64]. Deze studie richtte zich echter op een kleine groep betrokken ouders die gecoacht werden in CO-OP-strategieën, in plaats van psycho-educatie te volgen, en evalueerde niet de impact op de ouders zelf. In België spelen kinesitherapeuten en ergotherapeuten een cruciale rol in de ondersteuning van

het kind, de school en de ouders, terwijl psychologen ook een cruciale rol zouden kunnen spelen in dit proces. Dit is vooral belangrijk gezien de impact op het welzijn van zowel het kind als zijn familie. Veel kinderen in het onderzoek volgden sessies met een psycholoog, maar het is onduidelijk hoeveel ouders ook gebruik maakten van deze diensten. Verder onderzoek is nodig om te onderzoeken of gerichte ouderlijke coaching en psycho-educatie de stress binnen het gezin kunnen verlichten en zowel de resultaten voor het kind als het welzijn van de ouders kunnen verbeteren.

Door ouders gerapporteerde sterke punten bij kinderen

Dit onderzoek is het eerste grootschalige onderzoek naar door ouders gerapporteerde sterke punten bij kinderen met DCD. Er zijn overeenkomsten met sterke punten gerapporteerd bij andere ontwikkelingstoornissen zoals creativiteit, empathie en probleemoplossende vaardigheden [65, 66]. De hoge prevalentie van bijkomende aandoeningen kan deze bevindingen hebben beïnvloed. Verder onderzoek zal moeten uitwijzen of deze sterke punten specifiek gerelateerd zijn aan DCD, aan andere ontwikkelingstoornissen of aan neurodivergentie in het algemeen. Het erkennen van positieve eigenschappen bij neurodivergente personen is essentieel binnen een positieve psychologische benadering [67], omdat dit stigma kan verminderen, inclusie kan bevorderen en personen kan ondersteunen door in te spelen op hun sterke punten. Voor mensen met DCD die gevoelig zijn voor een laag zelfbeeld, kan het identificeren van hun sterke punten en het verbeteren van het gebruik ervan een waardevolle strategie zijn om het mentale welzijn te verbeteren.

Limitaties en sterktes van de studie

In dit onderzoek ontbrak een vergelijkingsgroep van typisch ontwikkelende kinderen of kinderen met andere ontwikkelingstoornissen, waardoor directe vergelijkingen niet mogelijk waren. Het werpt echter licht op specifieke gebieden die meer aandacht vragen bij veel van deze kinderen. Hoewel de onderzoeksopzet geen verificatie van de diagnose toeliet, konden door deze aanpak alle kinderen deelnemen, ongeacht of ze een formele diagnose hadden. Door de lengte van de enquête en de vereiste lees- en schrijfvaardigheid in het Nederlands of Frans, konden gezinnen die andere talen spreken of een lager lees- en schrijfniveau hebben, mogelijk niet deelnemen. Gezien de uitgebreide gegevens die al zijn verzameld, zullen verdere analyses zich richten op mogelijke beïnvloedende factoren zoals geslacht, leeftijd, sociaaleconomische status en geografische regio's van België.

Conclusie

Deze onderzoeksresultaten benadrukken de wijdverspreide impact van DCD vanuit het perspectief van ouders op meerdere domeinen. Het is belangrijk om kinderen niet alleen in motorische vaardigheden te ondersteunen, maar ook in andere ontwikkelingsdomeinen (zoals continentie, slaap en spraak) en contexten (zoals school en vrije tijd), met bijzondere aandacht voor emotioneel welzijn. Ouders melden bovendien verschillende sterke punten die het zelfvertrouwen en de ontwikkeling van kinderen kunnen bevorderen. De resultaten tonen ook aan dat ouders onvoldoende ondersteund worden, wat hun eigen mentale gezondheid negatief beïnvloedt. Er is een dringende behoefte aan meer bewustzijn bij scholen, de algemene bevolking en zorgprofessionals, zodat ze de kenmerken van DCD herkennen, de uitdagingen begrijpen en de juiste ondersteuning bieden. De volgende stap is onderzoeken hoe ouders beter ondersteund kunnen worden, het bewustzijn breder verspreid kan worden en hoe we samen met beleidsmakers effectieve strategieën kunnen ontwikkelen.

Erkenningen

We willen Bieke Samijn bedanken voor haar hulp bij het interpreteren van de resultaten in verband met zindelijkheid en Evelien D'Haeseleer voor haar hulp bij het interpreteren van de problemen met spraakarticulatie. We willen M. Van Vyve, M. Derom, H. Huyghe, S. Valles en F. Devroe bedanken voor hun hulp bij de vertaling van de enquête. Tot slot bedanken we L. D., G. Dewitte, B. De Mey, J. Ockerman, F. Deconinck, K. Klingels, M. Goetschalckx, S. Velghe en C. Johnson voor hun deelname aan de expertcommissie en de leden van Dyspraxis voor het constructief meedenken bij het vormgeven van de vragenlijst. Tot slot willen we Jason H. Spinks bedanken voor zijn waardevolle bijdrage aan de vertaling van dit manuscript naar het Frans, waardoor de verspreiding van deze resultaten is verbeterd.

Referenties

1. American Psychiatric Assocation. Diagnostic and Statistical Manual of Mental Disorders (5th text revision edition)(DSM-5-TR). Washington, 2022.
2. Zwicker JG, Harris SR, Klassen AF. Quality of life domains affected in children with developmental coordination disorder: a systematic review. Child Care Health Dev. 2013;39(4):562-80.
3. Missiuna C, Moll S, King S, King G, Law M. A trajectory of troubles: Parents' impressions of the impact of developmental coordination disorder. Phys Occup Ther Pediatr. 2007;27(1):81-101.
4. Cleaton MAM, Lorgelly PK, Kirby A. Developmental coordination disorder: the impact on the family. Qual Life Res. 2018;28(4).
5. Jijon AM, Leonard HC. Parenting stress in parents of children with developmental coordination disorder. Res Dev Disabil. 2020;104:103695.
6. Licari MK, Alvares GA, Bernie C, Elliott C, Evans KL, McIntyre S, et al. The unmet clinical needs of children with developmental coordination disorder. Pediatr Res. 2021;90(4):826-31.
7. Miller LT, Missiuna CA, Macnab JJ, Malloy-Miller T, Polatajko HJ. Clinical description of children with developmental coordination disorder. CJOT. 2001;68(1):5-15.
8. Farmer M, Echenne B, Bentourkia M. Study of clinical characteristics in young subjects with developmental coordination disorder. Brain Dev. 2016;38(6):538-47.
9. Flapper BC, Schoemaker MM. Developmental coordination disorder in children with specific language impairment: co-morbidity and impact on quality of life. Res Dev Disabil. 2013;34(2):756-63.
10. Missiuna C, Cairney J, Pollock N, Campbell W, Russell DJ, Macdonald K, et al. Psychological distress in children with developmental coordination disorder and attention-deficit hyperactivity disorder. Res Dev Disabil. 2014;35(5):1198-207.
11. Meachon EJ, Melching H, Alpers GW. The Overlooked Disorder: (Un)awareness of Developmental Coordination Disorder Across Clinical Professions. Adv Neurodev Disord. 2023;8:253-61.
12. Karkling M, Paul A, Zwicker JG. Occupational therapists' awareness of guidelines for assessment and diagnosis of developmental coordination disorder: Mesure selon laquelle les ergotherapeutes connaissent les lignes directrices relatives a l'evaluation et au diagnostic du trouble du developpement de la coordination. CJOT. 2017;84(3):148-57.
13. Maciver D, Owen C, Flannery K, Forsyth K, Howden S, Shepherd C, Rush R. Services for children with developmental co-ordination disorder: the experiences of parents. Child Care Health Dev. 2011;37(3):422-9.
14. Ahern K. Developmental Coordination Disorder: Validation of a Qualitative Analysis Using Statistical Factor Analysis. Int J Qual Methods. 2002;1(3):70-82.

15. Soriano CA, Hill EL, Crane L. Surveying parental experiences of receiving a diagnosis of developmental coordination disorder (DCD). Res Dev Disabil. 2015;43-44:11-20.
16. Wilson BN, Neil K, Kamps PH, Babcock S. Awareness and knowledge of developmental co-ordination disorder among physicians, teachers and parents. Child Care Health Dev. 2013;39(2):296-300.
17. Hunt J, Zwicker JG, Godecke E, Raynor A. Awareness and knowledge of developmental coordination disorder: A survey of caregivers, teachers, allied health professionals and medical professionals in Australia. Child Care Health Dev. 2021;47(2):174-83.
18. Khairati F, Stewart N, Zwicker JG. How developmental coordination disorder affects daily life: The adolescent perspective. Res Dev Disabil. 2024;144:104640.
19. Rodger S, Mandich A. Getting the run around: accesing services for children with developmental co-ordination disorder. Child Care Health Dev. 2005;31(4):449-57.
20. Novak C, Lingam R, Coad J, Emond A. 'Providing more scaffolding': Parenting a child with developmental co-ordination disorder, a hidden disability. Child Care Health Dev. 2012;38(6):829-35.
21. Mancini VO, Licari MK, Alvares GA, McQueen MC, McIntyre S, Reynolds JE, et al. Psychosocial wellbeing, parental concerns, and familial impact of children with developmental coordination disorder. Res Dev Disabil. 2024;145:104659.
22. Reynolds JE, Alvares GA, Williams J, Froude E, Elliott C, McIntyre S, et al. Investigating the impact of developmental coordination difficulties across home, school, and community settings: Findings from the Australian Impact for DCD survey. Res Dev Disabil. 2024;147:104712.
23. Klein ES, Cheung C, Garces A, Barbic S, Zwicker JG. Caregiver burden and mental health: Parent perspectives when raising a child with developmental coordination disorder. Res Dev Disabil. 2024;144:104656.
24. Klein ES, Licari M, Barbic S, Zwicker JG. Diagnostic services for developmental coordination disorder: Gaps and opportunities identified by parents. Child Care Health Dev. 2024;50(1):e13230.
25. Klein ES, Licari M, Barbic S, Zwicker JG. Success or failure? Are we meeting the needs of children with developmental coordination disorder? CJOT. 2023;91(2):00084174231197618.
26. Tamplain P, Miller HL, Peavy D, Cermak S, Williams J, Licari M. The impact for DCD – USA study: The current state of Developmental Coordination Disorder (DCD) in the United States of America. Res Dev Disabil. 2024;145:104658.
27. De Roubaix A, Van de Velde D, Van Waelvelde H. Parental report of early features of developmental coordination disorder: A qualitative study. Res Dev Disabil. 2023;143:104636.
28. Beaton DE, Bombardier C, Guillemin F, Ferraz MB. Guidelines for the process of cross-cultural adaptation of self-report measures. Spine. 2000;25(24):3186-91.
29. Goodman R. The Strengths and Difficulties Questionnaire: a research note. J Child Psychol Psyc. 1997;38(5):581-6.
30. Harris PA, Taylor R, Minor BL, Elliott V, Fernandez M, O'Neal L, et al. The REDCap consortium: Building an international community of software platform partners. J Biomed Inform. 2019; 95:103208.
31. Team J. JASP (version 0.14. 1). 2020.
32. Lumivero. Nvivo Software 14.24.4 (49). 2024.
33. Blank R, Barnett AL, Cairney J, Green D, Kirby A, Polatajko H, et al. International clinical practice recommendations on the definition, diagnosis, assessment, intervention, and psychosocial aspects of Developmental Coordination Disorder. DMCN. 2019;61(3):242-85.
34. Goos M, Pipa J, Peixoto F. Effectiveness of grade retention: A systematic review and meta-analysis. Educ Res Rev. 2021;34:100401.
35. Wu W, West SG, Hughes JN. Effect of grade retention in first grade on psychosocial outcomes. J Educ Psychol. 2010;102(1):135-52.

36. Tingle LR, Schoeneberger J, Algozzine B. Does grade retention make a difference? The Clearing House: A Journal of Educational Strategies, Issues and Ideas. 2012;85(5):179-85.
37. Piek JP, Edwards K. The identification of children with developmental coordination disorder by class and physical education teachers. Br J Educ Psychol. 1997;67(1):55-67.
38. De Roubaix A, Roeyers H, Van Waelvelde H, Bar-On L. Social responsiveness in children with developmental coordination disorder. BJPT. 2024;28(1):100591.
39. Lingam R, Jongmans MJ, Ellis M, Hunt LP, Golding J, Emond A. Mental health difficulties in children with developmental coordination disorder. Pediatrics. 2012;129(4):e882-91.
40. Barnett AL, Wiggs L. Sleep behaviour in children with developmental co-ordination disorder. Child Care Health Dev. 2012;38(3):403-11.
41. Chenier-Leduc G, Beliveau MJ, Dubois-Comtois K, Butler B, Berthiaume C, Pennestri MH. Sleep Difficulties in Preschoolers with Psychiatric Diagnoses. Int J Environ Res Public Health. 2019;16(22):11.
42. Wiggs K, Elmore AL, Nigg JT, Nikolas MA. Pre-and perinatal risk for attention-deficit hyperactivity disorder: Does neuropsychological weakness explain the link? J Abnorm Child Psychol. 2016;44:1473-85.
43. Rivilis I, Hay J, Cairney J, Klentrou P, Liu J, Faught BE. Physical activity and fitness in children with developmental coordination disorder: a systematic review. Res Dev Disabil. 2011;32(3):894-910.
44. Wiggs L, Sparrowhawk M, Barnett AL. Parent Report and Actigraphically Defined Sleep in Children with and without Developmental Coordination Disorder; Links with Fatigue and Sleepiness. Front pediatr. 2016;4:81.
45. Summers J, Larkin D, Dewey D. Activities of daily living in children with developmental coordination disorder: dressing, personal hygiene, and eating skills. Hum Mov Sci. 2008;27(2):215-29.
46. Lingam R, Golding J, Jongmans MJ, Hunt LP, Ellis M, Emond A. The association between developmental coordination disorder and other developmental traits. Pediatrics. 2010;126(5):e1109-18.
47. Archibald LM, Alloway TP. Comparing language profiles: Children with specific language impairment and developmental coordination disorder. Int J Lang Commun Disord. 2008;43(2):165-80.
48. Omer S, Jijon AM, Leonard HC. Research Review: Internalising symptoms in developmental coordination disorder: a systematic review and meta-analysis. J Child Psychol Psychiatry. 2018;60(6).
49. Meachon EJ, Zemp M, Alpers GW. Developmental Coordination Disorder (DCD): Relevance for Clinical Psychologists in Europe. Clin Psychol Eur. 2022;4(2):e4165.
50. Verlinden S, De Wijngaert P, Van den Eynde J. Developmental coordination disorder in adults: A case series of a condition that is underdiagnosed by adult psychiatrists. Psychiatry Research Case Reports. 2023;2(2):100148.
51. Vélez-Galarraga R, Guillen-Grima F, Crespo-Eguílaz N, Sánchez-Carpintero R. Prevalence of sleep disorders and their relationship with core symptoms of inattention and hyperactivity in children with attention-deficit/hyperactivity disorder. EJPN. 2016;20(6):925-37.
52. Meltzer LJ, Crabtree VM. Pediatric sleep problems: A clinician's guide to behavioral interventions: American Psychological Association; 2016.
53. Al Lihabi A. A literature review of sleep problems and neurodevelopment disorders. Front Psychiatry. 2023;14:1122344.
54. Bagley EJ, Kelly RJ, Buckhalt JA, El-Sheikh M. What keeps low-SES children from sleeping well: the role of presleep worries and sleep environment. Sleep Med. 2015;16(4):496-502.
55. Whiting S, Buoncristiano M, Gelius P, Abu-Omar K, Pattison M, Hyska J, et al. Physical activity, screen time, and sleep duration of children aged 6–9 years in 25 countries: an analysis within the WHO European childhood obesity surveillance initiative (COSI) 2015–2017. Obes facts. 2021;14(1):32-44.
56. Leader G, Francis K, Mannion A, Chen J. Toileting problems in children and adolescents with parent-reported diagnoses of autism spectrum disorder. J DEV PHYS DISABIL. 2018;30:307-27.

57. McKeown C, Hisle-Gorman E, Eide M, Gorman GH, Nylund CM. Association of constipation and fecal incontinence with attention-deficit/hyperactivity disorder. Pediatrics. 2013;132(5):e1210-5.
58. Nieuwhof-Leppink AJ, Schroeder RPJ, van de Putte EM, de Jong T, Schappin R. Daytime urinary incontinence in children and adolescents. Lancet Child Adolesc Health. 2019;3(7):492-501.
59. Butler RJ, Heron J. The prevalence of infrequent bedwetting and nocturnal enuresis in childhood. A large British cohort. Scand J Urol Nephrol. 2008;42(3):257-64.
60. Von Gontard A, Lettgen B, Olbing H, Heiken-Löwenau C, Gaebel E, Schmitz I. Behavioural problems in children with urge incontinence and voiding postponement: a comparison of a paediatric and child psychiatric sample. Br J Urol. 1998;81:100-6.
61. O'Kelly F, t'Hoen L, Silay S, Lammers R, Sforza S, Bindi E, et al. Neuropsychiatric developmental disorders in children are associated with an impaired response to treatment in bladder bowel dysfunction: a prospective multi-institutional European observational study. J Urol. 2023;210(6):899-907.
62. Tonge B, Brereton A, Kiomall M, Mackinnon A, King N, Rinehart N. Effects on parental mental health of an education and skills training program for parents of young children with autism: A randomized controlled trial. JAACAP. 2006;45(5):561-9.
63. Nussey C, Pistrang N, Murphy T. How does psychoeducation help? A review of the effects of providing information about Tourette syndrome and attention-deficit/hyperactivity disorder. Child Care Health Dev. 2013;39(5):617-27.
64. Araujo CRS, Cardoso AA, Polatajko HJ, de Castro Magalhães L. Efficacy of the Cognitive Orientation to daily Occupational Performance (CO-OP) approach with and without parental coaching on activity and participation for children with developmental coordination disorder: A randomized clinical trial. Res Dev Disabil. 2021;110:103862.
65. Maw KJ, Beattie G, Burns EJ. Cognitive strengths in neurodevelopmental disorders, conditions and differences: A critical review. Neuropsychologia. 2024;197:108850.
66. Schippers LM, Horstman LI, Pereira RR, Zinkstok J, Mostert JC, Greven CU, Hoogman M. A qualitative and quantitative study of self-reported positive characteristics of individuals with ADHD. Front Psychiatry. 2022;13:922788.
67. Seligman LD, Ollendick TH, Langley AK, Baldacci HB. The utility of measures of child and adolescent anxiety: a meta-analytic review of the Revised Children's Manifest Anxiety Scale, the State-Trait Anxiety Inventory for Children, and the Child Behavior Checklist. JCCAP. 2004;33(3):557-65.

Ondersteunende informatie

S1. File. Volledige vragenlijst in Nederlands en Frans.

S2. File. Percentage verdeling van niet significante variabelen die de aan- en afwezigheid van bijkomende aandoening vergelijkt.
