# Supplementary material for: Understanding the impact of developmental coordination disorder on Belgian children and families: A national survey study
Source: PLoS One. 2025 Apr 22;20(4):e0320311. doi: 10.1371/journal.pone.0320311 (PMC12013903; doi:10.1371/journal.pone.0320311)
Supplement: S4 File — (PDF) [file pone.0320311.s004.pdf]

Comprendre l'impact du trouble développemental de la coordination sur les enfants et les familles belges : une enquête nationale

Amy De Roubaix[(1*)], Griet Warlop[2], Dorine Van Dyck[3,4], Delphine Van Crombrugge[1], Silke Van den Abbeele[1], Melissa Licari[5], Hilde Van Waelvelde[1], Lynn Bar-On[1].

[1] Faculté de médecine et des sciences de la santé. Département des sciences de la réadaptation, Université de Gand, Gand, Belgique
[2] Faculté de médecine et des sciences de la santé. Département des sciences du mouvement et du sport, Université de Gand, Belgique
[3] Département de neuropsychologie et d'orthophonie, Hôpital universitaire pour enfants Reine Fabiola (HUDERF) - Hôpital universitaire de Bruxelles (H.U.B), Université libre de Bruxelles (ULB), Bruxelles, Belgique
[4] Département de neurologie pédiatrique, Hôpital universitaire pour enfants Reine Fabiola (HUDERF) - Hôpital universitaire de Bruxelles (H.U.B), Université libre de Bruxelles (ULB), Bruxelles, Belgique
[5] Telethon Kids Institute, Université d'Australie occidentale, Perth, Australie occidentale, Australie

*Auteur correspondant
Courrier électronique : amy.deroubaix@ugent.be

Résumé

Contexte. Le trouble développemental de la coordination (DCD) est une affection neurodéveloppementale méconnue et souvent banalisée qui touche cinq à six pourcents des enfants. Cette étude avait pour but de cartographier l'impact du DCD sur les enfants et leurs familles en Belgique.
Méthodes. Le questionnaire australien "Impact for DCD" a été traduit et adapté au contexte belge. Les parents d'enfants âgés de 4 à 18 ans vivant en Belgique et présentant des difficultés de mouvement correspondant au DCD ont été invités à répondre à l'enquête en ligne portant sur le diagnostic, les activités, l'école, la thérapie et l'impact social et émotionnel.
Résultats. Un total de 491 enfants ont été inclus dans les analyses. Les premières inquiétudes sont apparues principalement à la maison (61,4 %) à l'âge de 3,3±2,25 ans, avec une demande d'aide à l'âge de 4,7±2,57 ans. Le diagnostic formel a été posé à l'âge de 6,9±2,36 ans, les termes DCD (76,5%) et/ou dyspraxie (38,4%) étant les plus fréquemment utilisés. Le DCD était généralement méconnu, en particulier en milieu scolaire. Près d'un enfant sur quatre (23,2 %) avait redoublé une année scolaire. La qualité réduite du sommeil (50,6 %), la fatigue après l'école (76,0 %), les difficultés d'apprentissage de la propreté (47,9 %) et d'articulation de la parole (52,3 %), ainsi que les problèmes émotionnels importants (52,7 %) et les problèmes liés aux pairs (46,4 %), étaient prévalents. La plupart des enfants ont bénéficié d'une thérapie (89,2 %), mais 59,1 % des parents ne se sentaient pas suffisamment soutenus pour aider leur enfant. En outre, 37,5 % des parents prenaient régulièrement des congés pour faciliter la participation de leur enfant à la thérapie, tandis que 49,1 % avaient réduit leur temps de travail ou envisageaient de le faire (16,7 %). Les parents ont exprimé des inquiétudes quant à l'avenir et au bien-être de leur enfant, en demandant notamment des conseils sur le soutien à apporter à leur enfant et une plus grande sensibilisation. Les parents ont fait état de points forts importants chez leurs enfants, notamment l'empathie, la créativité, les capacités cognitives, la persévérance et de bonnes aptitudes sociales et/ou linguistiques.

Conclusions. Ces résultats mettent en évidence l'impact significatif du DCD du point de vue des parents. La prochaine étape consistera à explorer les moyens d’habiliter et de soutenir les parents, de les sensibiliser et de collaborer avec les décideurs politiques pour relever ces défis.

Déclarations

Publié en open source avec le soutien de la Fondation Universitaire de Belgique, du Legacy GE37 Children Rehabilitation Fund et du réseau de recherche scientifique ENLIGHT (RISE/SRN/ENtITY/1). Les auteurs n'ont pas d'intérêts concurrents à déclarer en rapport avec le contenu de cet article.

Introduction

Le trouble développemental de la coordination (DCD) est un trouble neurodéveloppemental (NDC) qui touche 5 à 6 % des enfants d'âge scolaire [1]. Les personnes atteintes de ce trouble éprouvent beaucoup plus de difficultés à acquérir et à exécuter des compétences motrices coordonnées par rapport à leurs pairs et aux possibilités d'apprentissage, ce qui a des répercussions importantes sur divers aspects de leur vie, tels que la productivité scolaire, les activités de la vie quotidienne et les activités professionnelles. Les difficultés sont présentes dès le plus jeune âge et ne peuvent être expliquées par une autre condition médicale (par exemple, la paralysie cérébrale, la dystrophie musculaire, la déficience intellectuelle). Les défis associés au DCD dépassent souvent les compétences motrices et affectent divers domaines de fonctionnement. Les difficultés évoquent un sentiment d'échec, ce qui entraîne des frustrations à court terme, une diminution de l'estime de soi et des risques de dépression et d'anxiété à long terme [2]. En conséquence, les individus évitent souvent l'activité physique et réduisent leur engagement, ce qui peut conduire à des compétences sociales diminuées, à moins d'amitiés et à un sentiment accru d'isolement [3]. Le DCD a des répercussions non seulement sur l'individu, mais aussi sur l'ensemble de la famille, qui s'engage moins dans des activités sociales et se sent limitée dans sa vie quotidienne [4]. Les parents peuvent ressentir une diminution de leur bien-être, des inquiétudes accrues et des exigences accrues en termes de temps et d'énergie pour répondre aux besoins de leur enfant, ce qui se traduit par un stress parental important [5]. Il est important de noter que les parents doivent souvent adapter leur carrière, réduire leur temps de travail ou cesser complètement de travailler, ce qui entraîne des difficultés financières en raison de la baisse de leurs revenus et de l'accès limité à un soutien financier [4, 6].

L'hétérogénéité du DCD est évidente non seulement dans la sévérité et les catégories de domaines touchés, mais aussi dans la cooccurrence avec d'autres conditions. Jusqu'à 70 % des enfants atteints de DCD souffriraient d'une ou de plusieurs autres pathologies, dont les troubles du spectre autistique (TSA), le trouble déficitaire de l'attention/hyperactivité (TDAH), les troubles spécifiques de l'apprentissage (par exemple, la dyslexie, la dyscalculie) et l'apraxie de la parole chez l'enfant [6-8]. Outre le fait qu'ils ont besoin de divers systèmes de soutien, il est essentiel de reconnaître que les enfants souffrant de troubles concomitants ont souvent des résultats encore plus médiocres, notamment une qualité de vie inférieure [9] et des problèmes de santé émotionnelle [10]. Alors que l'on estime qu'au moins un enfant dans chaque classe est atteint de DCD, cette pathologie fait partie des maladies infantiles les moins connues et est trop souvent méconnue [6]. En Allemagne et au Royaume-Uni, seuls 59 % des cliniciens de diverses spécialités connaissaient le DCD [11]. Au Canada, 100 % des ergothérapeutes pédiatriques ont démontré qu'ils connaissaient le DCD, contre 41 % des pédiatres et 22 % des médecins généralistes [12]. Le manque de sensibilisation des cliniciens s'ajoute à la sous-reconnaissance de la maladie, aux retards de diagnostic et à la minimisation des inquiétudes des parents, qui se sentent isolés et sans soutien [13-15]. Le secteur de l'éducation n'est pas non plus suffisamment sensibilisé au DCD, puisque seuls 23 % des enseignants canadiens [16] et 35 % des enseignants australiens [17] sont familiarisés avec le DCD. Le manque de sensibilisation au DCD dans les écoles peut entraîner une mauvaise interprétation et une banalisation des difficultés perçues, car au moins certains de ces enfants ont été considérés à tort comme "paresseux" et "ne faisant pas assez d'efforts [18, 19]. En ce qui concerne la sensibilisation du grand public, une étude canadienne a montré que seuls 6 % des parents avaient déjà entendu parler de DCD [16]. Les parents peuvent avoir du mal à comprendre la cause des difficultés de leur enfant et ne pas se rendre compte qu'il pourrait y avoir une maladie sous-jacente pouvant être diagnostiquée. Par conséquent, ils peuvent ressentir de la frustration lorsque leur

enfant éprouve des difficultés à accomplir des tâches élémentaires [20]. Cette frustration peut exacerber une dynamique familiale tendue et contribuer aux difficultés émotionnelles de l'enfant.

Le mouvement "Impact for DCD" a été créé dans le but de comprendre de manière exhaustive l'impact étendu du DCD et de plaider en faveur d'un changement systémique. Lancée initialement en Australie, l'équipe de Licari et al. (2021) a mis au point une vaste enquête visant à cartographier l'impact du DCD sur les enfants et leurs familles, et à identifier les domaines les plus importants pour les familles qui nécessitent des changements [6]. Depuis, de nombreux pays se sont joints à cet effort afin d'évaluer de manière exhaustive l'impact mondial. Il est essentiel de mener des études dans chaque pays, car l'impact peut varier considérablement en fonction du système de santé et d'éducation, de l'accès aux ressources, des réseaux de soutien social, de la culture et de la sensibilisation générale à la maladie. Jusqu'à présent, les résultats des recherches sur l'impact du DCD ont été publiés en Australie [6, 21, 22], au Canada [23-25] et aux États-Unis d'Amérique [26]. Cette étude vise à contribuer à ce mouvement en cartographiant l'impact du DCD en Belgique, qui a été largement inexplorée à l'exception d'une étude qualitative sur les caractéristiques précoces de la DCD [27]. L'identification des défis et des domaines d'amélioration dans le contexte belge pourrait aider à définir les domaines d'intervention et à prioriser le soutien aux familles. Les résultats de cette étude peuvent informer les décisions politiques relatives à l'éducation, aux soins de santé et aux services d'aide sociale.

Méthodes

Cette étude a été approuvée par le comité d'éthique médicale de l'hôpital universitaire de Gand (Belgique) (ONZ-2022-0203) et enregistrée sur ClinicalTrials.Gov (NCT05499143). Après avoir lu le formulaire de consentement éclairé, tous les participants ont donné leur consentement numérique en répondant positivement à la question de consentement avant de participer.

Enquête et participants

Conformément aux directives relatives au processus d'adaptation interculturelle des mesures d'auto-évaluation [28], l'enquête australienne Impact for DCD [6] a été traduite en néerlandais et en français et adaptée culturellement au contexte belge. Quatre interprètes ont traduit l'enquête originale en anglais vers le néerlandais (n=2) et le français (n=2). Ensuite, une synthèse des traductions néerlandaise et française a été réalisée au cours d'une réunion, après quoi quatre interprètes différents ont retraduit ces synthèses en anglais. Une réunion a été organisée pour discuter des différences d'interprétation qui nécessitaient une adaptation à la version néerlandaise et française. Des rapports écrits ont été fournis à chaque étape. Ensuite, un comité d'experts élargi (comprenant huit traducteurs, un parent d'un enfant atteint de DCD, un représentant de l'organisation belge sans but lucratif de soutien parental pour le DCD "Dyspraxis", quatre professionnels paramédicaux et huit chercheurs dans le domaine du DCD) a examiné l'enquête belge et discuté des questions de l'enquête. Comme dans l'enquête australienne, les questions portaient sur le diagnostic, la thérapie, les activités et la participation, l'éducation, l'impact social et émotionnel et l'impact sur la famille. Trois sous-échelles du Strengths and Difficulties Questionnaire (SDQ) [29] ont été utilisées : défis émotionnels, défis liés aux pairs et comportement prosocial. En l'absence de normes belges pour les enfants de 4 à 10 ans, les normes britanniques du SDQ ont été utilisées, tandis que les normes belges ont été utilisées pour les enfants de 11 à 18 ans. L'enquête originale a été complétée par plusieurs nouveaux thèmes, notamment l'apprentissage de la propreté, l'articulation de la parole et les points forts des enfants. L'enquête comprenait à la fois des

questions à choix multiples et des questions ouvertes et prenait en moyenne 45 minutes à remplir (fichier S1).

L'enquête a été lancée le 17 août 2022 et est restée disponible jusqu'au 17 décembre 2022. La collecte des données s'est appuyée sur les outils de saisie électronique REDCap hébergés à l'Université de Gand [30]. Les parents d'enfants âgés de 4 à 18 ans vivant en Belgique et présentant des difficultés de mouvement compatibles avec le DCD (c'est-à-dire ne pouvant être expliquées par une autre condition médicale) ont été invités à répondre à l'enquête en ligne. L'enquête s'est concentrée sur les expériences des parents, et toutes les réponses ont donc été fournies par les parents. L'étude a été promue par le biais de plateformes de médias sociaux, de la communication officielle de l'université et de l'organisation de soutien parental à but non lucratif Dyspraxis. Des invitations à participer ont été envoyées par courrier et par courriel à un large éventail de prestataires de soins de santé pédiatriques belges (par exemple, physiothérapeutes, ergothérapeutes, pédiatres, centres de rééducation, orthophonistes, psychomotriciens) et à des écoles d'enseignement spécialisé, afin d'assurer une couverture nationale. Les participants ont été exclus s'ils ne vivaient pas en Belgique, ne répondaient pas au critère d'âge (4-18 ans), n'avaient pas de difficultés de mouvement compatibles avec le DCD, avaient une autre condition médicale expliquant les difficultés de mouvement, ou n’avaient pas complètement rempli le questionnaire.

Analyse

Des analyses quantitatives et qualitatives ont été réalisées. L'étude des réponses ouvertes a permis d'obtenir des informations qualitatives qui ont complété les données de l'enquête, offrant ainsi une compréhension plus riche du sujet. Pour chaque question, le nombre total de participants ayant choisi une réponse particulière a été indiqué, ainsi que le pourcentage par rapport à l'ensemble de l'échantillon ou au sous-échantillon concerné lorsque des questions de suivi ont été posées. L'analyse quantitative effectuée dans JASP [31] comprenait une comparaison entre les réponses des enfants présentant ou non des troubles concomitants, qui ont été codés de manière binaire comme étant présents ou absents. En raison de la distribution non normale des données numériques, des tests Mann-Whitney U ont été utilisés, les tailles d'effet étant rapportées sous forme de r de rang-bisérien. Le niveau de signification a été fixé à $p<0,05$. Pour les données catégorielles, des tests du chi-carré ont été effectués. Une analyse thématique qualitative inductive a été réalisée pour discerner les concepts sous-jacents des questions ouvertes. Deux chercheurs ont codé indépendamment toutes les réponses à l'aide du logiciel NVivo 14 [32]. Les codes ont ensuite été organisés en thèmes par le biais d'un processus itératif. Toute divergence a été résolue en discutant. Pour renforcer la fiabilité de la collecte et de l'analyse des données, les critères d'inclusion et d'exclusion ont été abordés en détail dans la première partie du questionnaire. La triangulation des enquêteurs a été mise en œuvre par un double codage de toutes les questions ouvertes et par des discussions au sein de l'équipe de recherche pluridisciplinaire pour valider les résultats. En outre, une réflexivité accrue a été encouragée pour garantir une enquête approfondie et ouverte d'esprit.

Résultats

Les participants

Les parents de 1256 enfants ont initialement consenti à participer. Au total, 477 familles de 491 enfants ont été retenues pour l'analyse après exclusion des participants dont l'enfant ne vivait pas en Belgique (n=97), n'était pas âgé de 4 à 18 ans (n=19), ne présentait pas de difficultés de mouvement compatibles avec le DCD (n=22), avait une autre condition médicale pouvant expliquer

les difficultés de mouvement (n=329), ou parce que l'enquête n'a pas été complétée (n=298). Les caractéristiques de l'échantillon sont décrites dans le **Tableau 1**. L'échantillon de l'étude (n=491), dont l'âge moyen est de 10,4±3,37 ans, se compose principalement d'enfants de sexe masculin nés à terme, âgés de 7 à 12 ans, la majorité étant les enfants aînés, issus de familles à revenus moyens à élevés, résidant principalement en Flandre. Au moins un diagnostic supplémentaire était présent chez 59,5 % des enfants, le TDAH étant le trouble concomitant le plus répandu (31,0 %), suivi par les TSA (22,8 %) et les troubles spécifiques de l'apprentissage (21,8 %).

Tableau 1. Caractéristiques de l'échantillon (n=491).

	N	%
**Le sexe**		
Homme	373	76.0
Femme	118	24.0
**Âge gestationnel**		
<28 semaines	3	0.6
28 - 32 semaines	11	2.2
32 - 37 semaines	63	12.8
>37 semaines	412	83.9
Inconnu	2	0.4
**L'âge**		
4-6 ans	64	13.0
7-9 ans	145	29.5
10-12 ans	156	31.8
13-15 ans	80	16.3
16-18 ans	46	9.4
**Position dans la famille**		
Aîné	199	40.5
Moyen	55	11.2
Le plus jeune	169	34.4
Enfant unique	68	13.8
**Revenu familial net mensuel**		
Bas (< 1962 euros)	36	7.3
Moyen (1962 - 3924 euros)	190	38.7
Élevé (> 3924 euros)	205	41.8

Je préfère ne pas partager	60	12.2
**Région de résidence**		
Flandre	377	76.8
Wallonie	99	20.2
Région de Bruxelles-Capitale	15	3.1
**Affections concomitantes**		
TDAH	152	31.0
Troubles du spectre autistique	112	22.8
Trouble spécifique de l'apprentissage	107	21.8
Troubles de la parole et du langage	65	13.2
Dysgraphie	38	7.7
Anxiété ou dépression	24	4.9
Epilepsie	10	2.0
Problèmes de l'ouïe	4	0.8
Trouble du tic	3	0.6
Déficience visuelle cérébrale	3	0.6
Autres	19	3.9

Abréviations : TDAH, trouble déficitaire de l'attention avec hyperactivité ; n, nombre.

Diagnostic

Trajectoire diagnostique

Les inquiétudes concernant les mouvements de l'enfant ont été soulevées pour la première fois par les parents lorsque les enfants avaient en moyenne 3,3±2,25 ans. Ces inquiétudes ont été identifiées principalement à la maison (n=243, 61.4%), suivies par les rapports des écoles maternelles (2.5 à 5 ans) (n=156, 39.4%), des écoles primaires (6 à 12 ans) (n=39, 9.8%), ou des garderies (<2.5 ans) (n=38, 9.6%) (**Fig 1**). Les parents ont demandé de l'aide lorsque leurs enfants avaient un âge moyen de 4,7±2,57 ans. Au total, 80,7% (n=396) des enfants ont reçu un diagnostic formel à l'âge moyen de 6,9±2,36 ans. Il n'y avait pas de différence significative entre les enfants avec ou sans troubles concomitants en ce qui concerne l'âge de la première préoccupation (U = 28254,0, p = 0,74, r = -0,017 ; fichier S2) ou le diagnostic (U = 16641,0, p = 0,428, r = -0,048 ; fichier S2). Les termes diagnostiques les plus courants étaient DCD (n=294, 76,5%) et/ou dyspraxie (n=152, 38,4%) (**Tableau 2**), qui ont été principalement diagnostiqués par des neurologues (n=178, 44,9%), des centres de troubles du développement (n=120, 30,3%), des physiothérapeutes (n=112, 28,3%), ou des psychiatres (n=52, 13,1%) (**Fig 2**). Si un physiothérapeute a diagnostiqué les difficultés de mouvement, il a généralement collaboré avec un médecin (98 cas sur 112, 77,5%). Au cours du processus de diagnostic, des évaluations motrices (95,2%) et l'anamnèse parentale (87,6%) ont été fréquemment effectuées, ainsi que de nombreuses autres évaluations (**Fig 3**). Selon les parents, les enseignants ont été consultés dans 64,4% des cas. Chez 95 enfants (19,3 %), aucun diagnostic formel de difficultés de mouvement n'a été posé à ce jour, mais leurs difficultés de mouvement ont

plutôt été décrites comme, par exemple, "un risque / des caractéristiques de DCD", "des problèmes de coordination motrice" ou "des compétences motrices non fluentes" (**Tableau 2**).

Figure 1. Pourcentage des environnements qui ont suscité les premières inquiétudes (n=491) : les premières inquiétudes sont apparues principalement à la maison.

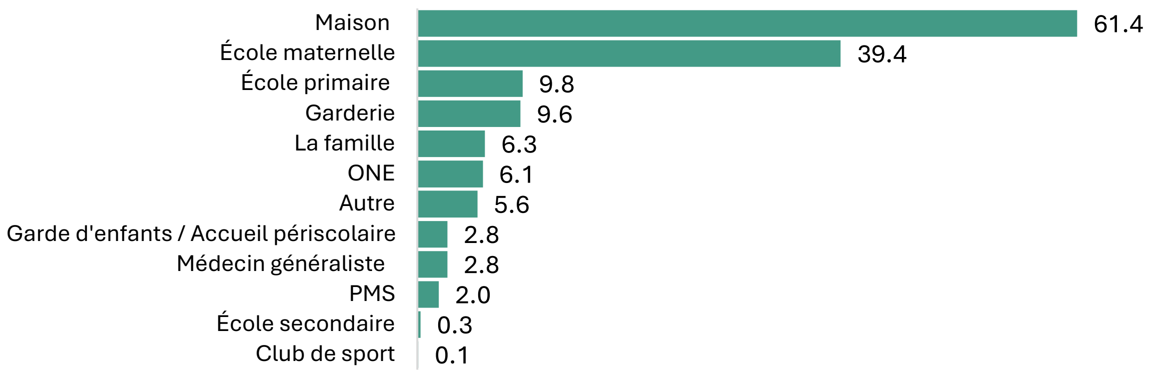

Les répondants pouvaient choisir plusieurs réponses.
Abréviations : ONE : Office de la Naissance et de l'Enfance (Il s'agit de l'agence gouvernementale flamande en Belgique responsable des services d'aide à l'enfance et à la famille) ; PMS : Centre Psycho-Médico-Social.

Figure 2. Professionnels (%) ayant formellement diagnostiqué des difficultés de mouvement chez l'enfant (n=396) : Divers professionnels et institutions posent le diagnostic de trouble de la coordination du développement.

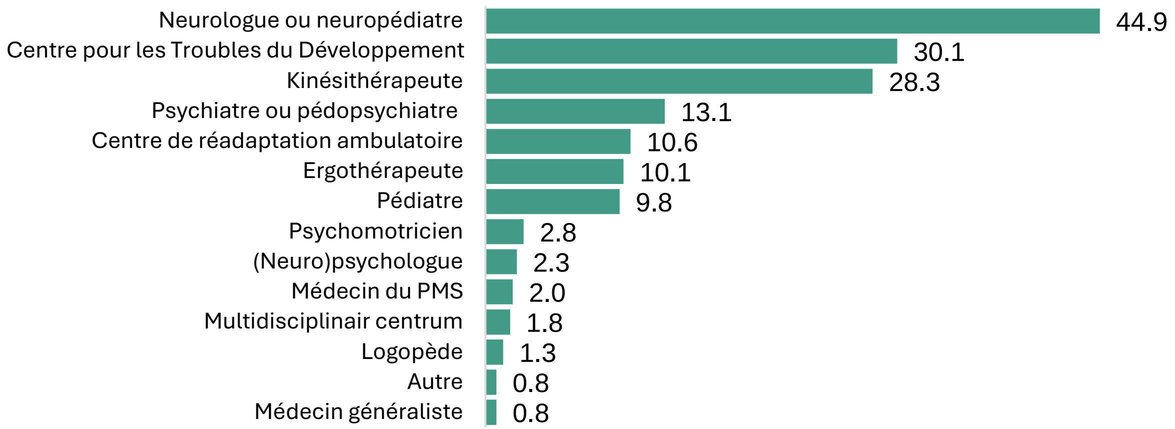

Les répondants pouvaient choisir plusieurs réponses.
Abréviations : PMS : Centre Psycho-Médico-Social.

Figure 3. Évaluations réalisées dans le cadre du processus de diagnostic des difficultés de mouvement (%) (n=396) : Les évaluations motrices et l'anamnèse parentale étaient courantes, tandis que les consultations avec les enseignants étaient moins fréquentes.

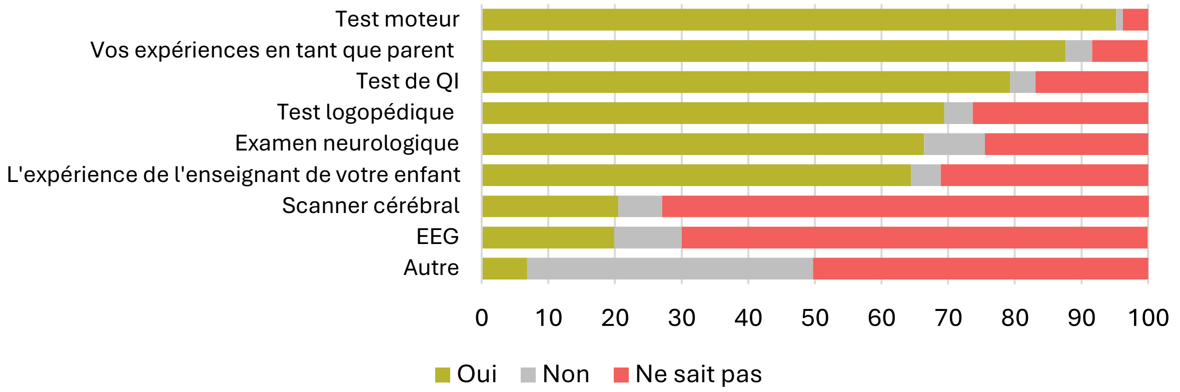

Les répondants pouvaient choisir plusieurs réponses.
Abréviations : QI, quotient intellectuel ; EEG, électro-encéphalogramme.

Tableau 2. Diagnostics et descriptions des difficultés de mouvement.

	N	%
**Diagnostic formel de difficultés de mouvement (n=396)**		
Trouble de la coordination du développement (DCD)*	294	74.2
Dyspraxie	152	38.4
Hypotonie	32	8.1
Hypermobilité	29	7.3
Trouble d'Acquisition de la Coordination (TAC)	8	2.0
Trouble de l'intégration sensorielle	1	0.3
Minimal Brain Damage	1	0.3
Autres	1	0.3
**Description des difficultés de mouvement en l'absence d'un diagnostic formel (n=95)**		
Risques / caractéristiques de DCD	57	60.0
Problèmes de coordination motrice	46	48.4
Motricité déficiente	46	48.4
Maladresse	45	47.4
Retard du développement moteur	30	31.6
Difficultés de planification motrice	25	26.3

Retard des étapes motrices	16	16.8
Non décrit par le professionnel	16	16.8
Trouble de l'intégration des réflexes	9	9.5
Autres	1	1.1

Les répondants pouvaient choisir plusieurs réponses.
*La catégorie DCD comprenait le terme anglais (Developmental Coordination Disorder), la traduction littérale en néerlandais (coördinatie ontwikkelingstoornis) et la traduction littérale en français (trouble développemental de la coordination; TDC).
Abréviations : DCD, Developmental Coordination Disorder (trouble de la coordination du développement) ; n, nombre.

Figure 4. Pourcentage de spécialistes, de milieux scolaires et d'amis/familles contactés par la famille qui connaissaient le diagnostic de trouble de la coordination du développement : La plupart des professionnels de la santé connaissaient le trouble de la coordination du développement, mais les connaissances étaient limitées dans les milieux éducatifs et de loisirs, ainsi que parmi la famille et les amis.

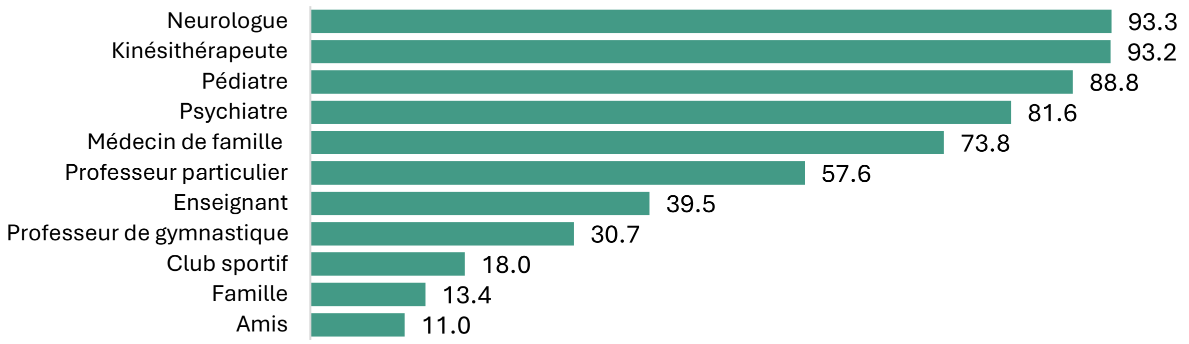

Sensibilisation et impact d'un diagnostic de DCD

La plupart des parents (n=232, 58,6 %) n'avaient pas entendu parler du DCD avant le diagnostic de leur enfant, mais ont reconnu que recevoir un diagnostic était bénéfique à la fois pour eux-mêmes (n=362, 91,4 %) et pour leur enfant (n=339). Lorsqu'on leur a demandé de préciser la valeur du diagnostic, les parents ont indiqué que pour leurs enfants, un diagnostic se traduisait par une meilleure compréhension de soi et une meilleure compréhension de la part des parents et des enseignants, un plus grand soutien à l'école et un accès à la thérapie (n=339). Toutefois, dans les cas où le diagnostic a été jugé inutile, les parents ont perçu un écart plus important entre leur enfant et les autres et un impact négatif sur la confiance en soi de l'enfant (n=57). Pour les parents (n=362), la confirmation du diagnostic impliquait la reconnaissance, la validation et la clarification de leurs préoccupations, favorisant l'acceptation et les rassurant sur le fait qu'ils n'imaginaient pas ou n'exagéraient pas les difficultés perçues, atténuant ainsi une période d'incertitude, de stress, de doute et de frustration. La confirmation du diagnostic a permis aux parents d'exprimer plus efficacement les difficultés de leur enfant, ce qui leur a permis de mieux les comprendre et d'éviter les remarques négatives de leur entourage. En outre, ils ont pu offrir un meilleur soutien, davantage de compréhension et de la patience. Cependant, les cas où les parents ne se sont pas sentis soutenus par le diagnostic tournaient principalement autour d'un manque d'assistance après le diagnostic et d'une incertitude permanente car ils devaient "tout comprendre par eux-mêmes"

(n=34). Alors que la plupart des médecins et des kinésithérapeutes consultés par la famille se sont montrés familiers avec le terme DCD, environ un médecin généraliste sur quatre ne connaissait pas le DCD (**Fig 4**). En outre, moins de la moitié des professeurs de classe et un tiers des professeurs d'éducation physique avaient une connaissance préalable du DCD. Enfin, 11 à 13 % des amis et de la famille avaient entendu parler de cette maladie.

Impact fonctionnel

Loisirs et activités sportives

Les parents ont déclaré que la plupart des enfants (n=372, 75,8%) participaient à des activités de loisirs organisées. Moins de la moitié des enfants (n=222, 45,2%) aiment participer à des activités sportives organisées, tandis que 35,2% (n=173) les apprécient parfois et 19,6% (n=96) pas du tout. Ces proportions ne diffèrent pas en fonction de la présence de pathologies concomitantes (χ^2 = 0,3, p = 0,596 et χ^2 = 3,0, p = 0,224 ; Fichier S2). Parmi les enfants, 82,5 % (n=405) ne pratiquaient pas 60 minutes d'activité physique modérée à vigoureuse par jour, sans différence entre les enfants avec ou sans troubles concomitants (χ^2 = 2,6, *p* = 0,454 ; fichier S2). Cependant, un tiers des parents (n=170, 34,6%) ont exprimé des inquiétudes concernant les effets négatifs potentiels d'une activité physique réduite sur la santé de leur enfant et 55,6% (n=273) ont rapporté un manque d'activités de loisirs disponibles adaptées aux besoins de leur enfant.

Activités de la vie quotidienne

Les parents ont déclaré que leurs enfants rencontraient des difficultés dans diverses activités de la vie quotidienne, dans la motricité fine et globale et dans la mobilité (englobant des aspects tels que la pratique du vélo et l'utilisation des transports en commun) (**Fig 5**). Environ la moitié des parents ont déclaré avoir rencontré des difficultés dans l'apprentissage de la propreté (n=235, 47,9%), y compris l'incontinence fécale (souillure ou encoprésie) après 4 ans (n=118, 24,0%), l'incontinence urinaire diurne après 5 ans (n=137, 27,9%), et l'énurésie après 5 ans (n=196, 39,9%). Aucun de ces résultats n'était significativement différent en présence d'affections concomitantes (χ^2 0,0-3,0, *p=0*,08-0,88, fichier S2). L'analyse qualitative (n=234) a révélé que l'apprentissage tardif et/ou prolongé de la propreté était une préoccupation commune à ces parents. Les parents ont indiqué que les problèmes liés à l'apprentissage de la propreté réapparaissaient en réponse au stress ou aux changements environnementaux, tels que les vacances ou le passage à une nouvelle année scolaire. Les difficultés motrices (par exemple, se déshabiller, viser, s'essuyer), les problèmes de comportement (par exemple, concentration, procrastination, refus d'aller aux toilettes) et les problèmes sensoriels (par exemple, mauvaise conscience du corps, difficulté à se détendre, préférence pour les lingettes humides) ont été couramment cités par les parents comme des facteurs contribuant à ces difficultés. La mère d'un enfant de six ans a décrit les difficultés de son enfant avec l'apprentissage de la propreté en ces termes : "Il ne savait pas comment aller aux toilettes : "Il ne savait pas comment se retenir, il faisait pipi à côté des toilettes (il le fait encore). Il ne savait pas comment viser et il ne sait toujours pas comment s'essuyer les fesses correctement. La moitié des enfants ont été signalés comme ayant une qualité de sommeil réduite (n=248, 50,6%), ce qui a été observé dans une plus large mesure chez les enfants souffrant de troubles concomitants (40,0% contre 57,9%, χ^2 = 15,2, p<0,001). Les analyses qualitatives ont indiqué que les parents (n=248) citaient principalement des difficultés d'endormissement de leurs enfants, attribuant souvent cela à des inquiétudes : "C'est un vrai penseur qui a du mal à trouver le sommeil. Il a souvent la tête pleine et ne trouve pas le repos". Les troubles du sommeil étaient fréquemment associés à des parasomnies, telles que des cauchemars, du somnambulisme et des discussions pendant le sommeil, avec parfois des rapports d'anxiété liée au sommeil.

Figure 5. Les parents ont déclaré que leurs enfants rencontraient des difficultés dans diverses activités (%) (n=491).

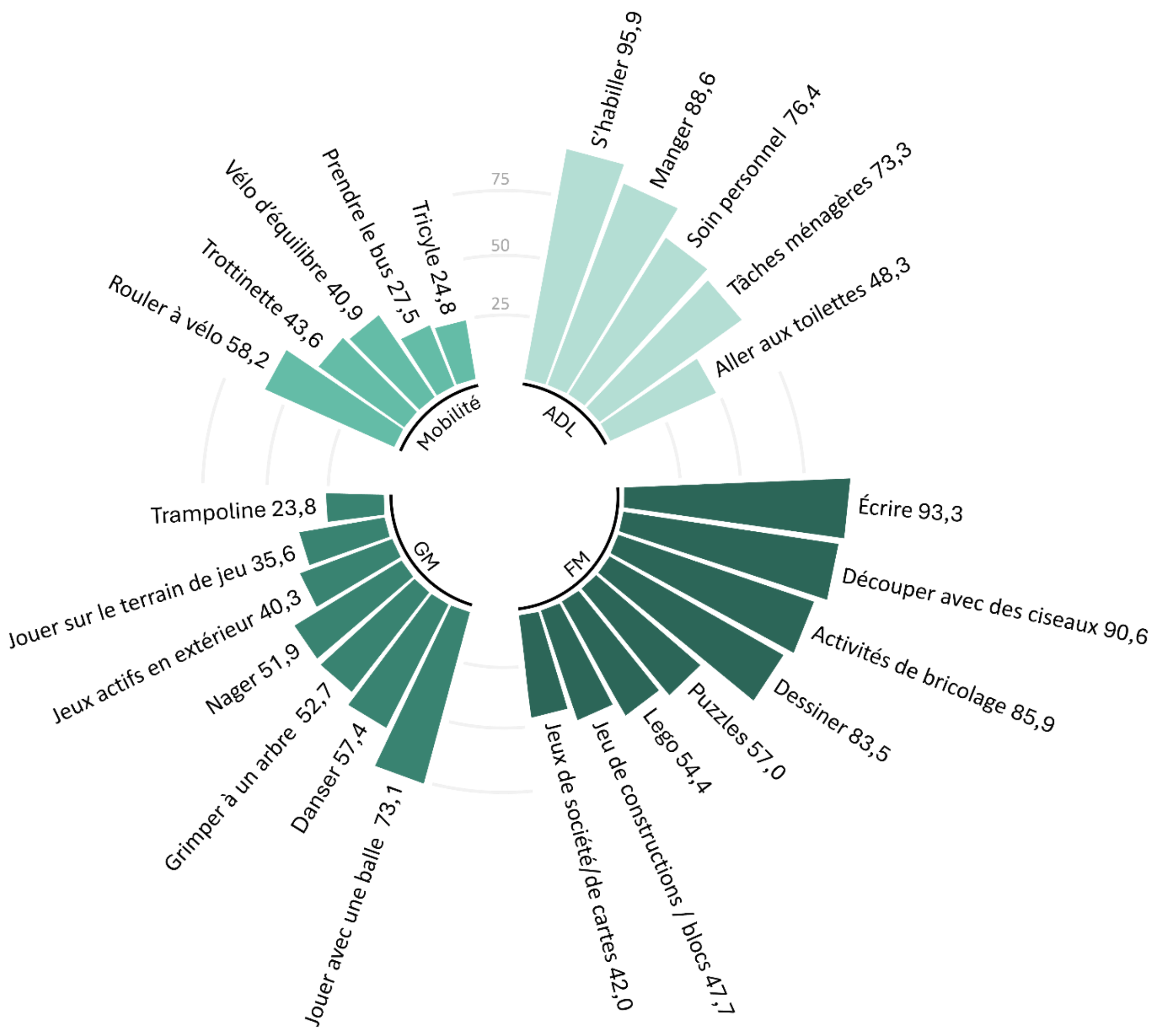

Abréviations : ADL, activités de la vie quotidienne ; FM, activités de motricité fine, GM, activités de motricité globale.

Articulation de la parole

Environ la moitié des parents (n=257, 52,3 %) ont fait part de leurs préoccupations concernant l'articulation de la parole de leurs enfants, avec plus d'inquiétudes chez les enfants souffrant de pathologies concomitantes (44,5 % contre 57,7 % ; $\chi^2 = 8,3$, p = 0,004). Les parents (n=250 ; 50,9%) ont signalé des problèmes liés à une articulation peu claire, y compris des erreurs phonétiques et phonologiques, telles que des difficultés de prononciation de sons spécifiques, des inversions de syllabes, des déformations de mots ou des erreurs de prononciation. En outre, des problèmes suprasegmentaux, comme le fait de parler trop vite, des problèmes de fluidité comme le bégaiement, ainsi que des altérations du volume de la voix, ont été fréquemment mentionnés. Ces problèmes sont parfois associés à un sentiment d'insécurité : "Elle a tendance à parler de manière imprécise, silencieuse et à marmonner. C'est généralement parce qu'elle ne se sent pas sûre d'elle. Cependant, elle peut articuler clairement, par exemple lorsqu'elle lit. En outre, des problèmes de

motricité orale, notamment une hypotonie et des problèmes de positionnement de la langue, ont été signalés, ainsi que de la bave et une tendance à garder la bouche ouverte.

École et éducation

Trajectoire scolaire et plaisir

La plupart des enfants ont suivi un enseignement normal, 78,8% (n=387) fréquentant des écoles ordinaires et 3,7% (n=18) des écoles didactiques alternatives (par exemple, Steiner, Montessori, Freinet). L'enseignement spécialisé était fréquenté par 13,0 % (n=64) et une minorité suivait un enseignement ordinaire inclusif (n=15, 3,1 %). Seuls sept enfants (1,4 %) ont suivi d'autres types d'enseignement (enseignement à domicile, enseignement privé, pas d'enseignement ou enseignement adapté aux enfants surdoués). Près d'un enfant sur quatre a dû redoubler au moins une année scolaire (n=114, 23,2%). Cette proportion était significativement plus importante chez les enfants présentant des troubles concomitants (16,5 % contre 27,8 % ; $\chi^2 = 8,5$, p = 0,003). L'analyse qualitative (n=114) a montré que les enfants redoublaient souvent la dernière année d'école maternelle (généralement vers 5 ans), principalement parce qu'ils étaient considérés comme immatures ou ne possédaient pas les capacités motrices nécessaires pour une transition réussie vers l'école primaire. Chez les enfants plus âgés, des retards généraux dans les compétences scolaires (lecture, langage, mathématiques) étaient à l'origine du redoublement d'une année d'école. Les parents avaient des sentiments mitigés quant au redoublement : "Il avait un professeur qui ne comprenait pas grand-chose [à ses problèmes] et qui lui disait qu'il était stupide et qu'il ne pouvait rien faire. Il n'avait plus du tout confiance en lui, alors [nous l'avons emmené dans] une nouvelle école [...] pour qu'il ait l'esprit tranquille."

Selon les parents, un enfant sur cinq (n=93, 18,9%) n'aimait pas du tout aller à l'école. L'analyse qualitative (n=188) a montré que les enfants subissaient un stress accru pour obtenir de bons résultats à l'école, tout en ayant l'impression d'être plus lents et "différents des autres" : "Il donne son maximum pour obtenir le minimum". En outre, les parents ont signalé des cas de harcèlement, des difficultés à établir des liens avec leurs pairs, un manque de compréhension et une fatigue accrue chez leurs enfants. Les parents s'inquiètent de l'épuisement de leur enfant à la fin de la journée scolaire, notant que les tâches prennent plus de temps à accomplir, que l'apprentissage devient plus difficile et qu'il y a peu de temps pour se détendre. La majorité des parents (n=373, 76,0%) ont rapporté une plus grande fatigue chez leur enfant par rapport à leurs pairs à la fin de la journée scolaire et cette proportion était encore plus élevée chez les enfants souffrant de troubles concomitants (70,0% contre 79,4% ; $\chi^2 = 4,6$, p = 0,03).

Communication et soutien à l'école

La majorité des enseignants ont été informés des difficultés de mouvement de l'enfant au début de l'année scolaire (n=410, 83,5%) et des réunions de parents ont été organisées soit uniquement avec l'enseignant de la classe (n=129, 26,3%), soit avec une équipe multidisciplinaire (n=261, 53,2%) pour discuter des besoins de l'enfant dans la classe. Au début de l'année scolaire, un contact entre le thérapeute de l'enfant et les enseignants a été établi pour environ la moitié des enfants (n=237, 48,3%). Selon les parents, des adaptations ont été mises en place par les enseignants pour 59,9% (n=294) des enfants, incluant souvent du temps supplémentaire pour les évaluations ou les examens (n=248, 50,5%). Un soutien supplémentaire a été fourni par l'enseignant de soutien pédagogique de l'école (n=147, 29,9%) et/ou un enseignant de soutien externe (n=229, 46,6%). En ce qui concerne les cours d'éducation physique, 39,4 % (n=190) des parents ont été informés que leur enfant bénéficiait d'une forme de soutien sur le site. En outre, 28,0% (n=138) des enfants ne se sentaient pas en confiance pour participer à des événements sportifs occasionnels à l'école, sans

qu'aucune différence significative n'ait été observée en fonction de la présence de troubles concomitants (χ^2 = 3,9, p = 0,149 ; fichier S2).

Thérapie et intervention

La plupart des enfants ont suivi une thérapie (n=438, 89,2%), la physiothérapie, l'orthophonie et la psychologie étant les plus fréquemment mentionnées (**Tableau 3**). Environ un enfant sur trois (n=143, 29,1%) a occasionnellement manqué des cours pour suivre une thérapie. Si plus de la moitié des parents estiment que leur enfant a bénéficié d'une thérapie suffisante pour traiter ses difficultés de mouvement (n=281, 57,2%), ils ont également déclaré qu'en tant que parents, ils ne se sentaient pas suffisamment soutenus pour aider leur enfant à surmonter ces difficultés (n=290, 59,1%). Un enfant sur dix (n=45, 9,2%) a pris des médicaments, principalement pour améliorer la concentration et l'attention en raison d'un diagnostic concomitant de TDAH.

Tableau 3. Aperçu des antécédents de suivi thérapeutique, du secteur de suivi thérapeutique et du suivi thérapeutique actuel par mois.

	Antécédents de suivi thérapeutique (%)			Secteur de fréquentation de la thérapie (%)				Participation à une thérapie en cours par mois (heures)		
	Oui	Non	Disparu	Privé	RCA	Autres	Disparu	Moyenne ± SD	Gamme	N*
**Physiothérapie**	78.0	8.1	13.8	69.2	15.1	1.8	13.8	3.6 ± 2.1	[0.5 - 12]	142/196
**Orthophonie**	59.9	22.4	17.7	62.9	19.4	4.1	13.6	3.8 ± 2.0	[0.5 - 10]	88/133
**Psychologie**	34.0	43.0	23.0	58.1	22.8	4.8	14.4	1.9 ± 1.2	[0.16 - 5]	39/62
**Ergothérapie**	29.9	46.2	23.8	38.1	40.8	8.8	12.2	4.3 ± 3.6	[0.5 -25)	53/83
**Neuropsychologie**	15.1	59.9	25.1	58.1	16.2	10.8	14.9	2.0 ±1.3	[0.5 - 4.0]	15/28
**Thérapie psychomotrice**	10.2	81.9	7.9	76.0	4.0	2.0	18.0	2.8 ± 1.2	[1.0 - 4.0]	8/11
**Autres**	14.3	29.9	55.8	65.7	7.1	18.6	8.6	3.1 ± 3.4	[0.25 - 12]	19/24

*En raison des réponses incomplètes à ces questions spécifiques, le nombre de répondants a été exprimé par rapport au nombre total de répondants qui suivent actuellement cette thérapie.
Abréviations : CAR, Centre Ambulatory Rehabilitation ; PT, physiothérapie ; OT, ergothérapie, SLT, orthophonie.

Impact socio-émotionnel

Sur le SDQ, environ la moitié des enfants présentaient des niveaux élevés de difficultés émotionnelles rapportées par les parents (n=259, 52,7%) et de problèmes liés aux pairs (n=228, 46,4%), tandis que la majorité d'entre eux affichaient un comportement pro-social dans la fourchette normale (n=365, 74,3%) (**Fig 6**). En outre, la moitié des parents ont déclaré que leur enfant avait des difficultés à se faire des amis (n=221, 45,0%). Toutes ces variables étaient significativement plus fréquentes chez les enfants souffrant de troubles concomitants, avec plus de difficultés émotionnelles (57,8% contre 68,5%, U = 24197,0, p = 0,001, *r* = -0,168), plus de problèmes liés aux pairs (48,7% contre 66.8% ; U = 22311.0, p < 0.001, *r*= -0.233), plus de difficultés à se faire des amis (33.0% contre 53.3% ; χ^2 = 19.7, p < 0.001), et des niveaux inférieurs de comportement pro-social (22.1% contre 28.1% ; U = 33293.5, p = 0.006, *r* = 0.144).

Figure 6. Distribution des performances (%) sur trois sous-tests du Questionnaire sur les forces et les difficultés (n=491) : Environ la moitié des enfants sont confrontés à des problèmes émotionnels et à des difficultés avec leurs pairs, bien que la majorité d'entre eux aient un comportement pro-social adéquat.

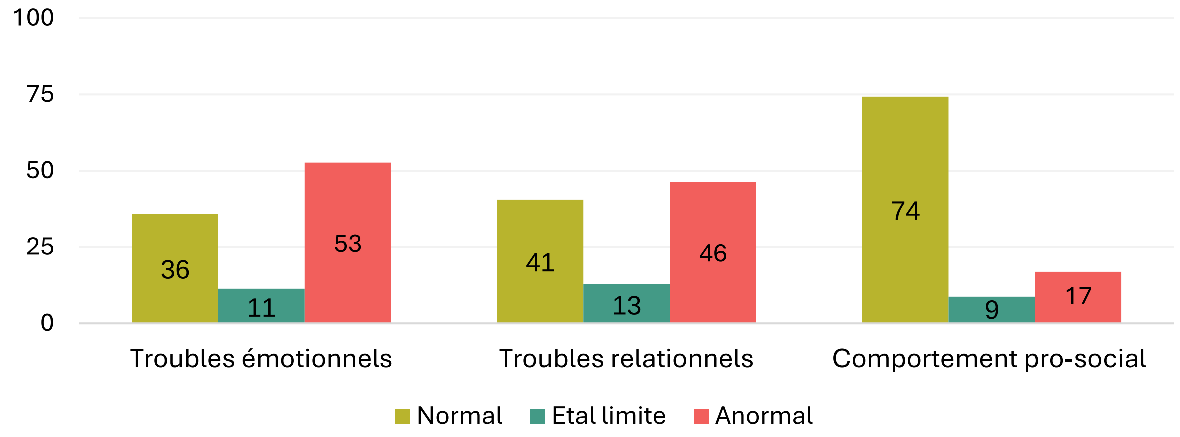

Impact sur la famille

Plus de la moitié des parents ont exprimé des inquiétudes quant à l'avenir de leur enfant, ont éprouvé des soucis émotionnels, ont trouvé peu de temps pour leurs besoins personnels et ont déclaré que le type d'activités familiales était limité (**Fig 7**). En outre, ils se sont inquiétés de l'impact négatif des problèmes moteurs sur la réussite scolaire de leur enfant (n=329, 67%) et sur ses futures activités professionnelles (n=318, 64,8%). Les parents ont également dû faire face à une charge financière (**Fig 7**). Des dépenses mensuelles pour la thérapie ont été rapportées pour 154 enfants (31,4%). En moyenne, ces dépenses s'élevaient à 144,21 € par mois (fourchette de 0 à 771 €). La majorité des parents n'ont reçu aucune aide financière supplémentaire pour la thérapie en dehors de la couverture de l'assurance maladie (n=358, 72,9%), bien que 22,8% (n=112) aient bénéficié d'une augmentation des allocations familiales. De nombreux parents (n=292, 59,5%) ont fait état de dépenses supplémentaires liées aux difficultés de mouvement de leur enfant, telles que du matériel scolaire adapté, des vêtements et des cours privés pour l'apprentissage d'habiletés motrices spécifiques. En outre, 37,5% (n=184) prenaient régulièrement des congés pour faciliter la participation de leur enfant à la thérapie, tandis que 49,1% (n=241) avaient réduit leur temps de travail ou envisageaient de le faire (16,7%, n=82).

L'analyse qualitative (n=471) a révélé que la principale priorité des parents est de remédier au manque de connaissance et de compréhension de la maladie, qui fait que les autres perçoivent souvent leurs inquiétudes comme exagérées. Ils expriment le besoin d'une orientation plus claire sur le soutien à apporter à leur enfant atteint de DCD, ainsi qu'un plaidoyer en faveur d'une réduction des charges administratives et d'une aide pour naviguer dans le système de soutien complexe. En outre, ils ressentent un manque de soutien, ce qui fait que nombre d'entre eux sont épuisés et peinent à trouver suffisamment de temps pour aider efficacement leur enfant.

Points forts des enfants atteints de DCD

L'identification des points forts déclarés a été réalisée par le biais d'une analyse qualitative (**Fig 8**). Les parents (n=476) ont déclaré que leurs enfants étaient très empathiques, attentionnés et avaient

un sens aigu de la justice, tandis que la créativité était observée dans les capacités de résolution de problèmes, l'expression artistique et le sens de l'humour. En outre, ils ont souligné les capacités cognitives de leurs enfants, qui englobent l'intelligence, une mémoire forte et la curiosité. La persévérance, la résilience et l'optimisme ont également été décrits. L'un des parents a expliqué cette caractéristique en déclarant : "Elle fait tout ce qu'elle peut pour avancer. Elle fait tout ce qu'elle peut pour faire ce qu'on lui demande, et si elle ne réussit pas du premier coup, elle essaie encore jusqu'à ce qu'elle réussisse". Un sous-ensemble de parents a mis l'accent sur les compétences linguistiques de leurs enfants, y compris de solides compétences verbales, le multilinguisme et la maîtrise de la lecture. Enfin, de bonnes compétences sociales ont été mentionnées. Une mère résume la situation : "Notre fils est incroyablement créatif, il sort des sentiers battus, mais il est surtout très social, drôle, ouvert d'esprit, il a un très grand cœur, il est très empathique et peut parfaitement réconcilier les parties et désarmer une situation difficile."

Figure 7. L'impact multiforme des difficultés de déplacement sur les familles : les parents s'inquiètent surtout de l'avenir et du bien-être émotionnel de leur enfant et perçoivent un manque de temps pour leurs besoins personnels (n=491) (%).

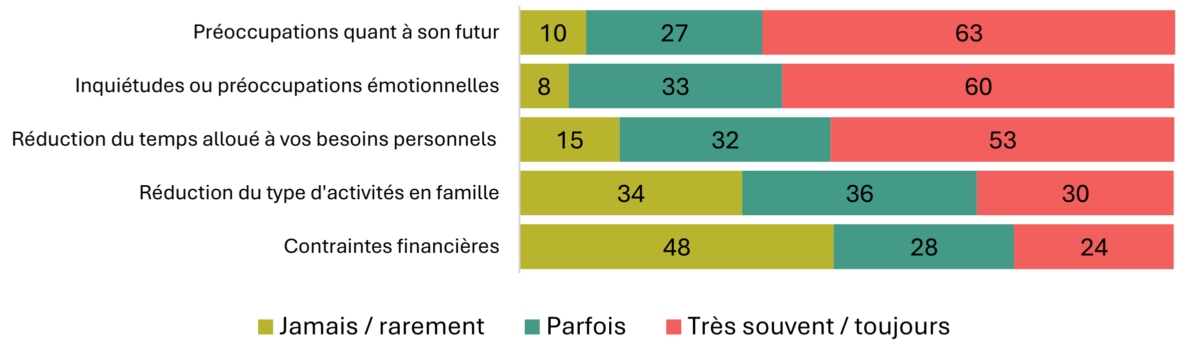

Figure 8. Points forts déclarés par les parents chez les enfants souffrant d'un trouble de la coordination du développement (n=476).

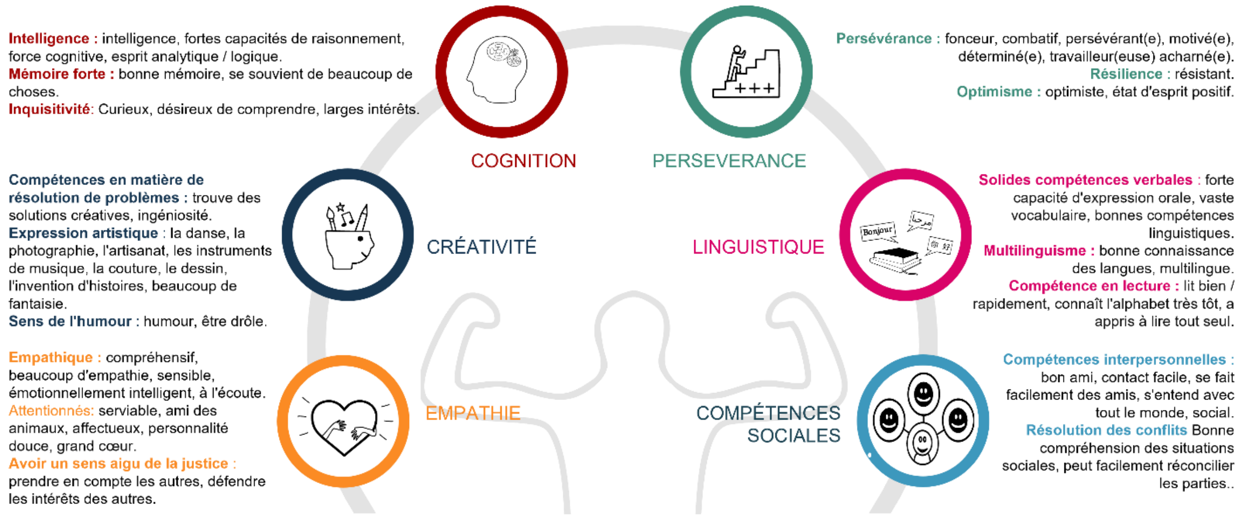

Discussion

Cette enquête, qui porte sur 491 expériences parentales d'enfants belges, met en évidence l'impact considérable de la maladie sur l'enfant et la famille. En moyenne, les enfants ont reçu un diagnostic deux ans après leur première recherche d'aide. Cependant, un enfant sur cinq n'a pas (encore) reçu de diagnostic formel pour ses difficultés de mouvement. Malgré des efforts tels que les réunions de soutien scolaire et l'accès à la thérapie, les parents indiquent un manque de soutien adéquat, d'activités de loisirs adaptées et de sensibilisation générale, ce qui affecte le bien-être de l'enfant et le leur. Il est essentiel de reconnaître et de relever ces défis tout en reconnaissant les points forts de ces enfants pour leur assurer un meilleur avenir. Il est important de reconnaître que cette étude ne comporte pas de groupe de contrôle, ce qui limite notre capacité à comparer ces expériences avec celles d'enfants au développement normal.

Trajectoire diagnostique et soutien

De nombreux professionnels de la santé en Belgique utilisent le terme diagnostique correct de "trouble de la coordination du développement", bien que des termes descriptifs tels que "dyspraxie" soient encore utilisés. Les directives internationales de diagnostic du DCD sont bien connues en Belgique, ce qui explique peut-être la plus grande adhésion à la terminologie diagnostique correcte et l'âge légèrement plus élevé au moment du diagnostic (6,9 ans) par rapport aux résultats australiens (5,3 ans) et américains (4,9 ans) [6, 26]. Les directives internationales [33] recommandent spécifiquement de ne pas diagnostiquer les enfants avant l'âge de cinq ans, ce qui a probablement influencé le moment où les diagnostics ont été posés. Il est intéressant de noter que la présence de pathologies concomitantes n'a pas eu d'impact significatif sur l'âge des préoccupations ou du diagnostic. Cependant, ces troubles peuvent détourner l'attention des difficultés motrices, retardant ainsi leur identification, alors qu'à l'inverse, les évaluations multidisciplinaires pour les enfants présentant des difficultés multiples peuvent accélérer l'identification des difficultés motrices. Néanmoins, l'écart de deux ans entre la demande d'aide et la confirmation du diagnostic est important, et l'accès au soutien n'est pas clair pendant cette période. La majorité des familles ont déclaré que l'obtention d'un diagnostic avait été très bénéfique, soulignant la nécessité d'un diagnostic correct et opportun. Des recherches supplémentaires sont nécessaires pour explorer l'impact d'un diagnostic précoce sur le stress parental et les résultats pour l'enfant. En Australie et au Canada [6, 24], les parents ont également joué un rôle essentiel dans l'identification précoce des problèmes, mais plus de la moitié d'entre eux n'avaient jamais entendu parler de la maladie auparavant, ce qui a probablement retardé la première recherche d'aide. Une plus grande sensibilisation des parents au DCD peut contribuer à un dépistage précoce. Enfin, les parents ne se sentent pas suffisamment soutenus pour aider leur enfant après le diagnostic. Nos résultats soulignent donc la nécessité d'intensifier les efforts pour fournir aux parents un soutien avant et après le diagnostic.

Il est intéressant de noter que les préoccupations ont été plus fréquemment exprimées dans les établissements préscolaires belges (39,4 %) qu'au Canada (8,0 %), ce qui pourrait s'expliquer par la durée prolongée de l'éducation préscolaire (trois ans et demi en Belgique contre un an au Canada) [24]. Contrairement aux lignes directrices internationales [33], les enseignants n'ont pas souvent été inclus dans le processus de diagnostic. L'intégration des enseignants dans le processus de diagnostic et de soutien pourrait faciliter un diagnostic opportun et améliorer la sensibilisation et le soutien aux enfants atteints de TCD dans les écoles. Cela est nécessaire, car de nombreux enfants de notre étude ont dû redoubler au moins une année scolaire, une pratique dont l'efficacité est sujette à débat. L'impact du redoublement sur les progrès scolaires pourrait être limité [34] et bien

que l'on observe des améliorations à court terme du bien-être émotionnel, les résultats à long terme suggèrent des effets négatifs potentiels sur l'acceptation sociale [35]. Par ailleurs, Tingle, Schoeneberger et Algozzine [36] préconisent un enseignement de rattrapage plutôt qu'un maintien en classe, ce qui a été le cas pour moins de la moitié des enfants inclus dans cette étude. Cependant, des réunions impliquant les parents et/ou les thérapeutes, ainsi que des adaptations pour l'enfant en classe, ont eu lieu pour environ la moitié des enfants, ce qui démontre les efforts déployés pour répondre aux divers besoins d'apprentissage. En revanche, la communication entre les parents et les enseignants d'éducation physique était plus limitée. Malgré leur formation en développement moteur, la majorité des professeurs d'éducation physique interrogés dans le cadre de cette étude n'étaient pas familiarisés avec le DCD. Pourtant, la littérature antérieure indique que lorsqu'on leur demande d'identifier les enfants susceptibles d'obtenir de mauvais résultats à un test de motricité, les professeurs d'éducation physique identifient correctement la moitié des enfants, ce qui souligne leur potentiel en tant que ressource inexploitée pour la détection des caractéristiques du DCD à l'école [37].

Des défis généralisés

Dans cette enquête, nous avons examiné les difficultés perçues par les enfants atteints de DCD et confirmé des difficultés sociales et émotionnelles accrues [38, 39], des troubles du sommeil [40-42], une diminution de l'activité physique [43], de la fatigue [44], des problèmes d'apprentissage de la propreté [27, 45], des difficultés d'articulation de la parole [46, 47] et un impact considérable sur la famille [4-6].

Les niveaux élevés de difficultés sociales et émotionnelles soulignent l'importance d'aborder ces aspects, étant donné que les personnes atteintes de DCD courent un risque accru de développer une dépression et de l'anxiété [48]. Les résultats soulignent en outre la nécessité d'une sensibilisation accrue au DCD dans le secteur de la santé mentale [49], car la présence potentielle d'un DCD sous-jacent est souvent négligée lorsque les personnes recherchent de l'aide [50]. Bien qu'il s'agisse d'une cible d'intervention prometteuse, aucune étude n'a été réalisée à ce jour sur l'efficacité de la psychothérapie pour les personnes atteintes de DCD. La cause et la nature de la baisse de la qualité du sommeil signalée dans cette étude doivent faire l'objet de recherches plus approfondies, car la prévalence signalée de 50,6 % dans cette étude est plus élevée que celle observée chez les enfants au développement normal (3-36 %) [51, 52]. Alors que chez les personnes atteintes de TSA et de TDAH, les problèmes de sommeil ont été associés à des facteurs tels que le dysfonctionnement de la régulation de la mélatonine, l'apnée obstructive du sommeil et le trouble du rythme circadien du sommeil [53], ils pourraient également être liés à un moindre bien-être émotionnel entraînant une augmentation des inquiétudes avant le sommeil [54]. Il est étonnant de constater que 82,5 % des enfants de notre échantillon d'étude n'ont pas atteint les 60 minutes d'activité physique quotidienne modérée à vigoureuse recommandées, ce qui est quatre fois plus élevé que les 19,6 % rapportés chez les enfants âgés de six à neuf ans dans la région européenne de l'OMS [55]. Ces résultats vont dans le sens des lignes directrices internationales qui préconisent de mettre davantage l'accent sur la condition physique des personnes atteintes de troubles du développement. Malgré les tendances mondiales indiquant une baisse de l'activité physique chez les enfants [55], les enfants neurodivergents peuvent avoir besoin d'un soutien supplémentaire pour apprécier le mouvement. La diminution de l'activité physique peut entraîner une fatigue accrue chez ces enfants, alors que la fatigue peut également entraîner une diminution de l'activité physique. En outre, étant donné que presque tous les aspects de la vie quotidienne exigent une coordination motrice, ce qui est particulièrement difficile pour les enfants atteints de DCD, il n'est pas surprenant que la plupart des parents fassent état d'une fatigue accrue chez leurs enfants. À ce jour, les

difficultés liées à l'apprentissage de la propreté sont bien connues dans le TSA [56] et le TDAH [57], mais restent largement méconnues dans le DCD. La prévalence de l'incontinence urinaire diurne (27,9 %) et de l'énurésie (39,9 %) rapportée après 5 ans dans cette étude dépasse largement la prévalence dans la population, qui est respectivement de 10 % [58] et de 21 % [59]. L'apprentissage de la propreté implique non seulement une série d'habiletés motrices coordonnées telles qu'ouvrir la tirette, l'orientation vers les toilettes et l'essuyage, mais nécessite également, entre autres, une planification et une organisation efficaces (par exemple, programmer les pauses toilettes pendant la récréation, assurer l'accès au papier toilette s'il n'est pas disponible), une orientation spatiale (localiser les toilettes) et une gestion des stimuli sensoriels (ressentir l'envie, faire face à certaines sensations). Il est essentiel de détecter ces problèmes d'apprentissage de la propreté, car ils sont souvent liés à des difficultés psychosociales et à des problèmes avec les pairs [60] et sont associés à une mauvaise réponse au traitement [61]. Dans notre échantillon, les difficultés d'articulation (52,3 %) ont été signalées chez près de quatre fois plus d'enfants que ceux chez qui a diagnostiqué des troubles du langage (13,2 %). Il est intéressant de noter que les diagnostics de troubles du langage étaient moins fréquents dans l'échantillon belge que dans d'autres pays (23 à 40 %) [6, 24, 26]. L'articulation n'est qu'un aspect du processus complexe de production de la parole qui nécessite une coordination importante des muscles contrôlant l'expiration et le réglage fin des sons que nous produisons (c'est-à-dire la langue, la bouche et la mâchoire). La production de la parole peut être influencée par la confiance en soi, mais aussi par les difficultés de coordination motrice orale déjà identifiées chez les enfants atteints de DCD [46, 47]. Bien que cette étude n'ait pas évalué la gravité des problèmes d'articulation de la parole, il est essentiel de reconnaître qu'ils peuvent affecter de manière significative et indépendante le bien-être émotionnel et la qualité de vie en général. Les défis décrits ci-dessus soulignent l'importance de mettre en œuvre des approches holistiques et personnalisées.

Les résultats de l'étude confirment en outre que l'impact peut aller au-delà de l'individu et se répercuter sur les activités quotidiennes et le bien-être des parents, ce qui incite ces derniers à réclamer une meilleure connaissance des troubles de l'apprentissage et des conseils pour soutenir efficacement leur enfant. Cependant, la plupart des études d'intervention se sont principalement concentrées sur le "traitement" de l'enfant atteint de DCD. À ce jour, aucun programme formel de soutien aux parents pour DCD (comprenant par exemple une psychoéducation) n'a été étudié, bien qu'il ait été prouvé qu'il était bénéfique dans d'autres NDC [62, 63]. Une seule étude a comparé la formation à l'orientation cognitive vers les performances professionnelles quotidiennes (CO-OP) chez des enfants atteints de DCD, avec et sans accompagnement parental, mais elle n'a révélé aucune différence statistique entre les groupes en termes d'amélioration des performances professionnelles et motrices des enfants [64]. Cependant, cette étude s'est concentrée sur un petit groupe de parents engagés qui ont bénéficié d'un coaching sur les stratégies CO-OP plutôt que d'une psychoéducation et n'a pas mesuré l'impact sur les parents eux-mêmes. En Belgique, les kinésithérapeutes et les ergothérapeutes jouent un rôle essentiel dans le soutien à l'enfant, à l'école et aux parents, tandis que les psychologues pourraient également jouer un rôle crucial dans ce processus. Les psychologues pourraient également jouer un rôle crucial dans ce processus. Ceci est particulièrement important étant donné l'impact sur le bien-être de l'enfant et de sa famille. De nombreux enfants ayant participé à l'enquête ont suivi des séances avec un psychologue, mais le nombre de parents qui ont également bénéficié de ces services n'est pas clair. Des recherches supplémentaires sont nécessaires pour déterminer si un coaching parental et une psychoéducation ciblés peuvent atténuer le stress familial et améliorer les résultats de l'enfant ainsi que le bien-être des parents.

Points forts des enfants selon les parents

Cette étude représente la première enquête à grande échelle sur les forces rapportées par les parents chez les enfants atteints de DCD. Il existe des similitudes avec les points forts signalés dans d'autres NDC, tels que la créativité, l'empathie et la capacité à résoudre des problèmes [65, 66]. La forte prévalence de troubles concomitants peut avoir influencé ces résultats. D'autres recherches devront préciser si ces points forts sont liés spécifiquement au DCD, aux NDC cooccurrents ou à la neurodivergence en général. Reconnaître les traits positifs des personnes neurodivergentes est essentiel dans le cadre d'une approche de psychologie positive [67], car cela peut réduire la stigmatisation, promouvoir l'inclusion et soutenir les personnes en utilisant leurs attributs positifs. Pour les personnes atteintes de DCD qui sont susceptibles d'avoir une faible estime d'elles-mêmes, l'identification de leurs points forts et l'amélioration de leur utilisation peuvent constituer une stratégie précieuse pour stimuler le bien-être mental.

Limites et points forts de l'étude

Cette étude ne comportait pas de groupe de comparaison composé d'enfants au développement normal ou d'enfants atteints d'autres NDC, ce qui empêche les comparaisons directes. Toutefois, elle met en lumière les domaines spécifiques nécessitant une attention particulière au sein d'une vaste cohorte d'enfants. Bien que la conception de l'étude n'ait pas permis de vérifier le diagnostic, cette approche a permis d'inclure tous les enfants, qu'ils aient ou non reçu un diagnostic formel. En raison de la longueur de l'enquête et de la nécessité de savoir lire et écrire en néerlandais ou en français, les familles qui parlent d'autres langues ou qui ont un niveau d'alphabétisation plus faible auraient pu être empêchées de participer. Compte tenu des nombreuses données déjà communiquées, d'autres comparaisons en fonction de variables d'influence potentielles telles que le sexe, l'âge, le statut socio-économique et les régions géographiques de la Belgique feront l'objet d'une analyse plus approfondie.

Conclusion

Les résultats de cette étude mettent clairement en évidence l'impact généralisé et significatif du DCD, du point de vue des parents, dans de multiples domaines. Nous devons soutenir les enfants non seulement dans leurs capacités motrices, mais aussi dans d'autres domaines de développement (par exemple, la continence, le sommeil, l'articulation de la parole) et dans d'autres contextes (par exemple, l'école et les activités de loisirs), en mettant particulièrement l'accent sur le bien-être émotionnel. En outre, les parents font état de plusieurs points forts qui peuvent être utilisés pour renforcer la confiance en soi et le développement global des enfants. Les résultats soulignent que les parents ne sont pas suffisamment soutenus et que leur propre santé mentale est également affectée. En outre, il est urgent de sensibiliser les écoles, la population en général et les professionnels de la santé. Cela est essentiel pour s'assurer qu'ils peuvent reconnaître les caractéristiques du trouble déficitaire de l'attention, comprendre les défis qui y sont associés et fournir un soutien approprié. La prochaine étape cruciale consiste à étudier comment mieux responsabiliser et soutenir les parents, à sensibiliser plus largement et à engager des discussions avec les décideurs politiques et les parties prenantes afin de concevoir ensemble des stratégies efficaces pour surmonter ces défis.

Remerciements

Nous tenons à remercier Bieke Samijn pour son aide dans l'interprétation des variables relatives à l'apprentissage de la propreté et Evelien D'Haeseleer pour son aide dans l'interprétation des difficultés d'articulation de la parole. Nous remercions M. Van Vyve, M. Derom, H. Huyghe, S. Valles et F. Devroe pour leur aide dans la traduction de l'enquête. Enfin, nous remercions L. D., G. Dewitte, B. De Mey, J. Ockerman, F. Deconinck, K. Klingels, M. Goetschalckx, S. Velghe et C. Johnson pour leur participation au comité d'experts, ainsi que les membres de Dyspraxis pour leur contribution constructive à l'élaboration du questionnaire. Enfin, nous exprimons notre gratitude à Jason H. Spinks pour sa précieuse contribution à la traduction de ce manuscrit en français, ce qui a permis d'améliorer la diffusion de ces résultats.

Références

1. American Psychiatric Assocation. Manuel diagnostique et statistique des troubles mentaux (5e édition révisée) (DSM-5-TR). Washington, 2022.
2. Zwicker JG, Harris SR, Klassen AF. Quality of life domains affected in children with developmental coordination disorder : a systematic review. Child Care Health Dev. 2013;39(4):562-80.
3. Missiuna C, Moll S, King S, King G, Law M. A trajectory of troubles : Parents' impressions of the impact of developmental coordination disorder. Phys Occup Ther Pediatr. 2007;27(1):81-101.
4. Cleaton MAM, Lorgelly PK, Kirby A. Trouble de la coordination du développement : l'impact sur la famille. Qual Life Res. 2018;28(4).
5. Jijon AM, Leonard HC. Parenting stress in parents of children with developmental coordination disorder. Res Dev Disabil. 2020;104:103695.
6. Licari MK, Alvares GA, Bernie C, Elliott C, Evans KL, McIntyre S, et al. The unmet clinical needs of children with developmental coordination disorder. Pediatr Res. 2021;90(4):826-31.
7. Miller LT, Missiuna CA, Macnab JJ, Malloy-Miller T, Polatajko HJ. Description clinique des enfants présentant un trouble de la coordination du développement. CJOT. 2001;68(1):5-15.
8. Farmer M, Echenne B, Bentourkia M. Etude des caractéristiques cliniques de jeunes sujets présentant un trouble de la coordination du développement. Brain Dev. 2016;38(6):538-47.
9. Flapper BC, Schoemaker MM. Developmental coordination disorder in children with specific language impairment : co-morbidity and impact on quality of life. Res Dev Disabil. 2013;34(2):756-63.
10. Missiuna C, Cairney J, Pollock N, Campbell W, Russell DJ, Macdonald K, et al. Psychological distress in children with developmental coordination disorder and attention-deficit hyperactivity disorder. Res Dev Disabil. 2014;35(5):1198-207.
11. Meachon EJ, Melching H, Alpers GW. The Overlooked Disorder : (Un)awareness of Developmental Coordination Disorder Across Clinical Professions. Adv Neurodev Disord. 2023;8:253-61.
12. Karkling M, Paul A, Zwicker JG. Occupational therapists' awareness of guidelines for assessment and diagnosis of developmental coordination disorder : Mesure selon laquelle les ergotherapeutes connaissent les lignes directrices relatives a l'evaluation et au diagnostic du trouble du developpement de la coordination. CJOT. 2017;84(3):148-57.
13. Maciver D, Owen C, Flannery K, Forsyth K, Howden S, Shepherd C, Rush R. Services for children with developmental co-ordination disorder : the experiences of parents. Child Care Health Dev. 2011;37(3):422-9.
14. Ahern K. Developmental Coordination Disorder : Validation of a Qualitative Analysis Using Statistical Factor Analysis. Int J Qual Methods. 2002;1(3):70-82.

15. Soriano CA, Hill EL, Crane L. Surveying parental experiences of receiving a diagnosis of developmental coordination disorder (DCD). Res Dev Disabil. 2015;43-44:11-20.
16. Wilson BN, Neil K, Kamps PH, Babcock S. Awareness and knowledge of developmental co-ordination disorder among physicians, teachers and parents. Child Care Health Dev. 2013;39(2):296-300.
17. Hunt J, Zwicker JG, Godecke E, Raynor A. Awareness and knowledge of developmental coordination disorder : A survey of caregivers, teachers, allied health professionals and medical professionals in Australia. Child Care Health Dev. 2021;47(2):174-83.
18. Khairati F, Stewart N, Zwicker JG. How developmental coordination disorder affects daily life : The adolescent perspective. Res Dev Disabil. 2024;144:104640.
19. Rodger S, Mandich A. Getting the run around : accesing services for children with developmental co-ordination disorder. Child Care Health Dev. 2005;31(4):449-57.
20. Novak C, Lingam R, Coad J, Emond A. "Providing more scaffolding" : Parenting a child with developmental co-ordination disorder, a hidden disability. Child Care Health Dev. 2012;38(6):829-35.
21. Mancini VO, Licari MK, Alvares GA, McQueen MC, McIntyre S, Reynolds JE, et al. Psychosocial wellbeing, parental concerns, and familial impact of children with developmental coordination disorder. Res Dev Disabil. 2024;145:104659.
22. Reynolds JE, Alvares GA, Williams J, Froude E, Elliott C, McIntyre S, et al. Investigating the impact of developmental coordination difficulties across home, school, and community settings : Findings from the Australian Impact for DCD survey. Res Dev Disabil. 2024;147:104712.
23. Klein ES, Cheung C, Garces A, Barbic S, Zwicker JG. Caregiver burden and mental health : Parent perspectives when raising a child with developmental coordination disorder. Res Dev Disabil. 2024;144:104656.
24. Klein ES, Licari M, Barbic S, Zwicker JG. Diagnostic services for developmental coordination disorder : Lacunes et opportunités identifiées par les parents. Child Care Health Dev. 2024;50(1):e13230.
25. Klein ES, Licari M, Barbic S, Zwicker JG. Succès ou échec ? Are we meeting the needs of children with developmental coordination disorder ? CJOT. 2023;91(2):00084174231197618.
26. Tamplain P, Miller HL, Peavy D, Cermak S, Williams J, Licari M. The impact for DCD - USA study : The current state of Developmental Coordination Disorder (DCD) in the United States of America. Res Dev Disabil. 2024;145:104658.
27. De Roubaix A, Van de Velde D, Van Waelvelde H. Parental report of early features of developmental coordination disorder : A qualitative study. Res Dev Disabil. 2023;143:104636.
28. Beaton DE, Bombardier C, Guillemin F, Ferraz MB. Guidelines for the process of cross-cultural adaptation of self-report measures (Lignes directrices pour le processus d'adaptation interculturelle des mesures d'auto-évaluation). Spine. 2000;25(24):3186-91.
29. Goodman R. The Strengths and Difficulties Questionnaire : a research note. J Child Psychol Psyc. 1997;38(5):581-6.
30. Harris PA, Taylor R, Minor BL, Elliott V, Fernandez M, O'Neal L, et al. The REDCap consortium : Building an international community of software platform partners. J Biomed Inform. 2019 ; 95:103208.
31. Équipe J. JASP (version 0.14. 1). 2020.
32. Lumivero. Nvivo Software 14.24.4 (49). 2024.
33. Blank R, Barnett AL, Cairney J, Green D, Kirby A, Polatajko H, et al. International clinical practice recommendations on the definition, diagnosis, assessment, intervention, and psychosocial aspects of Developmental Coordination Disorder. DMCN. 2019;61(3):242-85.
34. Goos M, Pipa J, Peixoto F. Effectiveness of grade retention : A systematic review and meta-analysis. Educ Res Rev. 2021;34:100401.
35. Wu W, West SG, Hughes JN. Effect of grade retention in first grade on psychosocial outcomes. J Educ Psychol. 2010;102(1):135-52.

36. Tingle LR, Schoeneberger J, Algozzine B. Does grade retention make a difference ? The Clearing House : A Journal of Educational Strategies, Issues and Ideas. 2012;85(5):179-85.
37. Piek JP, Edwards K. The identification of children with developmental coordination disorder by class and physical education teachers. Br J Educ Psychol. 1997;67(1):55-67.
38. De Roubaix A, Roeyers H, Van Waelvelde H, Bar-On L. Social responsiveness in children with developmental coordination disorder. BJPT. 2024;28(1):100591.
39. Lingam R, Jongmans MJ, Ellis M, Hunt LP, Golding J, Emond A. Mental health difficulties in children with developmental coordination disorder. Pediatrics. 2012;129(4):e882-91.
40. Barnett AL, Wiggs L. Sleep behaviour in children with developmental co-ordination disorder. Child Care Health Dev. 2012;38(3):403-11.
41. Chenier-Leduc G, Beliveau MJ, Dubois-Comtois K, Butler B, Berthiaume C, Pennestri MH. Sleep Difficulties in Preschoolers with Psychiatric Diagnoses (Difficultés de sommeil chez les enfants d'âge préscolaire ayant un diagnostic psychiatrique). Int J Environ Res Public Health. 2019;16(22):11.
42. Wiggs K, Elmore AL, Nigg JT, Nikolas MA. Pre-and perinatal risk for attention-deficit hyperactivity disorder : Does neuropsychological weakness explain the link ? J Abnorm Child Psychol. 2016;44:1473-85.
43. Rivilis I, Hay J, Cairney J, Klentrou P, Liu J, Faught BE. Physical activity and fitness in children with developmental coordination disorder : a systematic review. Res Dev Disabil. 2011;32(3):894-910.
44. Wiggs L, Sparrowhawk M, Barnett AL. Parent Report and Actigraphically Defined Sleep in Children with and without Developmental Coordination Disorder ; Links with Fatigue and Sleepiness. Front pediatr. 2016;4:81.
45. Summers J, Larkin D, Dewey D. Activities of daily living in children with developmental coordination disorder : dressing, personal hygiene, and eating skills. Hum Mov Sci. 2008;27(2):215-29.
46. Lingam R, Golding J, Jongmans MJ, Hunt LP, Ellis M, Emond A. The association between developmental coordination disorder and other developmental traits. Pediatrics. 2010;126(5):e1109-18.
47. Archibald LM, Alloway TP. Comparing language profiles : Children with specific language impairment and developmental coordination disorder. Int J Lang Commun Disord. 2008;43(2):165-80.
48. Omer S, Jijon AM, Leonard HC. Research Review : Internalising symptoms in developmental coordination disorder : a systematic review and meta-analysis. J Child Psychol Psychiatry. 2018;60(6).
49. Meachon EJ, Zemp M, Alpers GW. Developmental Coordination Disorder (DCD) : Relevance for Clinical Psychologists in Europe. Clin Psychol Eur. 2022;4(2):e4165.
50. Verlinden S, De Wijngaert P, Van den Eynde J. Developmental coordination disorder in adults : A case series of a condition that is underdiagnosed by adult psychiatrists. Psychiatry Research Case Reports. 2023;2(2):100148.
51. Vélez-Galarraga R, Guillen-Grima F, Crespo-Eguílaz N, Sánchez-Carpintero R. Prevalence of sleep disorders and their relationship with core symptoms of inattention and hyperactivity in children with attention-deficit/hyperactivity disorder. EJPN. 2016;20(6):925-37.
52. Meltzer LJ, Crabtree VM. Pediatric sleep problems : A clinician's guide to behavioral interventions : American Psychological Association ; 2016.
53. Al Lihabi A. A literature review of sleep problems and neurodevelopment disorders. Front Psychiatry. 2023;14:1122344.
54. Bagley EJ, Kelly RJ, Buckhalt JA, El-Sheikh M. What keeps low-SES children from sleeping well : the role of presleep worries and sleep environment. Sleep Med. 2015;16(4):496-502.
55. Whiting S, Buoncristiano M, Gelius P, Abu-Omar K, Pattison M, Hyska J, et al. Physical activity, screen time, and sleep duration of children aged 6-9 years in 25 countries : an analysis within the WHO European childhood obesity surveillance initiative (COSI) 2015-2017. Obes facts. 2021;14(1):32-44.
56. Leader G, Francis K, Mannion A, Chen J. Toileting problems in children and adolescents with parent-reported diagnostics of autism spectrum disorder. J DEV PHYS DISABIL. 2018;30:307-27.

57. McKeown C, Hisle-Gorman E, Eide M, Gorman GH, Nylund CM. Association of constipation and fecal incontinence with attention-deficit/hyperactivity disorder. Pediatrics. 2013;132(5):e1210-5.
58. Nieuwhof-Leppink AJ, Schroeder RPJ, van de Putte EM, de Jong T, Schappin R. Daytime urinary incontinence in children and adolescents. Lancet Child Adolesc Health. 2019;3(7):492-501.
59. Butler RJ, Heron J. The prevalence of infrequent bedwetting and nocturnal enuresis in childhood. A large British cohort. Scand J Urol Nephrol. 2008;42(3):257-64.
60. Von Gontard A, Lettgen B, Olbing H, Heiken-Löwenau C, Gaebel E, Schmitz I. Behavioural problems in children with urge incontinence and voiding postponement : a comparison of a paediatric and child psychiatric sample. Br J Urol. 1998;81:100-6.
61. O'Kelly F, t'Hoen L, Silay S, Lammers R, Sforza S, Bindi E, et al. Neuropsychiatric developmental disorders in children are associated with an impaired response to treatment in bladder bowel dysfunction : a prospective multi-institutional European observational study. J Urol. 2023;210(6):899-907.
62. Tonge B, Brereton A, Kiomall M, Mackinnon A, King N, Rinehart N. Effects on parental mental health of an education and skills training program for parents of young children with autism : A randomized controlled trial. JAACAP. 2006;45(5):561-9.
63. Nussey C, Pistrang N, Murphy T. How does psychoeducation help ? A review of the effects of providing information about Tourette syndrome and attention-deficit/hyperactivity disorder. Child Care Health Dev. 2013;39(5):617-27.
64. Araujo CRS, Cardoso AA, Polatajko HJ, de Castro Magalhães L. Efficacité de l'approche Cognitive Orientation to daily Occupational Performance (CO-OP) avec et sans coaching parental sur l'activité et la participation pour les enfants souffrant de troubles de la coordination : A randomized clinical trial. Res Dev Disabil. 2021;110:103862.
65. Maw KJ, Beattie G, Burns EJ. Cognitive strengths in neurodevelopmental disorders, conditions and differences : A critical review. Neuropsychologia. 2024;197:108850.
66. Schippers LM, Horstman LI, Pereira RR, Zinkstok J, Mostert JC, Greven CU, Hoogman M. A qualitative and quantitative study of self-reported positive characteristics of individuals with ADHD. Front Psychiatry. 2022;13:922788.
67. Seligman LD, Ollendick TH, Langley AK, Baldacci HB. The utility of measures of child and adolescent anxiety : a meta-analytic review of the Revised Children's Manifest Anxiety Scale, the State-Trait Anxiety Inventory for Children, and the Child Behavior Checklist. JCCAP. 2004;33(3):557-65.

Informations complémentaires

S1. File. Enquête complète néerlandais/français.

S2. File. Distribution en pourcentage des variables non significatives comparant entre la présence et l'absence de conditions cooccurrentes.
